# Characterization of RNP Networks of PUM1 and PUM2 Post-Transcriptional Regulators in TCam-2 Cells, a Human Male Germ Cell Model

**DOI:** 10.3390/cells9040984

**Published:** 2020-04-16

**Authors:** Maciej J. Smialek, Erkut Ilaslan, Marcin P. Sajek, Aleksandra Swiercz, Damian M. Janecki, Kamila Kusz-Zamelczyk, Tomasz Wozniak, Maciej Kotecki, Luiza Handschuh, Marek Figlerowicz, Jadwiga Jaruzelska

**Affiliations:** 1 Institute of Human Genetics, Polish Academy of Sciences, Strzeszynska 32, 60-479 Poznan, Poland; erkut.ilaslan@igcz.poznan.pl (E.I.); marcin.sajek@igcz.poznan.pl (M.P.S.); kamila.kusz-zamelczyk@igcz.poznan.pl (K.K.-Z.); tomasz.wozniak@igcz.poznan.pl (T.W.); 2 Institute of Bioorganic Chemistry, Polish Academy of Sciences, Noskowskiego 12/14, 61-704 Poznan, Poland; aleksandra.swiercz@cs.put.poznan.pl (A.S.); djanecki@ibch.poznan.pl (D.M.J.); luizahandschuh@ibch.poznan.pl (L.H.); marekf@ibch.poznan.pl (M.F.); 3 Institute of Computing Science, Poznan University of Technology, Piotrowo 2, 60-965 Poznan, Poland; 4 Department of Developmental, Molecular and Chemical Biology, Tufts University Medical School, 136 Harrison Ave, Boston, MA 02111, USA; mkkotecki@gmail.com

**Keywords:** 3′UTR, human infertility, mass spectrometry, post-transcriptional gene regulation, ribonucleoprotein complexes, RNA-binding proteins (RBPs), testicular germ cell tumors

## Abstract

Mammalian Pumilio (PUM) proteins are sequence-specific, RNA-binding proteins (RBPs) with wide-ranging roles. They are involved in germ cell development, which has functional implications in development and fertility. Although human PUM1 and PUM2 are closely related to each other and recognize the same RNA binding motif, there is some evidence for functional diversity. To address that problem, first we used RIP-Seq and RNA-Seq approaches, and identified mRNA pools regulated by PUM1 and PUM2 proteins in the TCam-2 cell line, a human male germ cell model. Second, applying global mass spectrometry-based profiling, we identified distinct PUM1- and PUM2-interacting putative protein cofactors, most of them involved in RNA processing. Third, combinatorial analysis of RIP and RNA-Seq, mass spectrometry, and RNA motif enrichment analysis revealed that PUM1 and PUM2 form partially varied RNP-regulatory networks (RNA regulons), which indicate different roles in human reproduction and testicular tumorigenesis. Altogether, this work proposes that protein paralogues with very similar and evolutionary highly conserved functional domains may play divergent roles in the cell by combining with different sets of protein cofactors. Our findings highlight the versatility of PUM paralogue-based post-transcriptional regulation, offering insight into the mechanisms underlying their diverse biological roles and diseases resulting from their dysfunction.

## 1. Introduction

Post-transcriptional gene regulation (PTGR) is crucial for maintaining cellular proteome homeostasis [1,2], disruption of which can cause severe diseases, such as cancer and infertility [3]. PTGR requires the activity of RNA-binding proteins (RBPs), such as the widely studied pumilio (PUM) proteins, which are founding members of the PUF (pumilio and fem-3 binding factor) family of eukaryotic RBPs. PUM proteins are highly conserved and present in many organisms, from yeast to humans (for review, see [4]). Simultaneous knockout of mouse PUM1 and PUM2 is lethal [5], indicating their crucial role in development. Post-transcriptional regulation by PUMs is mediated by the conserved C-terminal RNA-binding PUF domain, which is composed of eight tandem repeats [6], and binds a specific eight nucleotide sequence 5′-UGUAHAUA-3′ (H represents A, C or U, but not G), called the PUM-binding element (PBE) that is typically located in the 3′ untranslated regions (3′UTR) of target mRNAs. By binding PBEs, PUMs trigger the recruitment of protein cofactors, that together direct selected mRNAs towards post-transcriptional repression or activation (for review see [4]).

Each of the five PUMs in yeast (Puf proteins) contains a PUF domain that is different in structure from the others, contains between six and eight tandem repeats, and binds to a distinct PBE motif. In this way, each Puf co-ordinately controls the fate of multiple mRNAs sharing a specific PBE motif and which have been found to be functionally related [7]. These findings became the basis for the so-called PUM/Puf RNA regulon model [8]. Considering the high structural similarity of PUM1 and PUM2, it is still unresolved whether they form separate regulons in mammals. Although mammalian PUM1 and PUM2 contain nearly identical PUF domains [9] that recognize the same PBE motif (UGUANAUA) [10], there is some evidence for divergent modes of regulation. Examination of interactions between another RBP, ARGONAUTE2 (AGO2), and PUM proteins revealed a substantial fraction of nonoverlapping PUM1 and PUM2 mRNA targets [11]. Therefore, it is possible that PUM1 and PUM2 paralogues are functionally nonredundant and function as distinct RNA regulons. We have recently demonstrated a specific example of functional nonredundancy between PUM1 and PUM2 by showing that while PUM2 induces PBE-dependent repression of the mRNA target SIAH1, PUM1 does so in a PBE-independent manner [12]. Additionally, the regions N-terminal to the PUF domain, which are divergent between PUM1 and PUM2, were reported to contain three unique subregions with autonomous repressive activity that may represent an interface for binding protein cofactors since they were not demonstrated to bind RNA [13]. 

A number of PUM protein cofactors, such as NANOS1, NANOS3, and DAZ family members, are associated with male or female infertility in humans [14,15,16,17,18,19]. Therefore, establishing the mechanisms underlying functional divergence of PUM1 and PUM2, including identification of their protein cofactors, may help in understanding their particular roles in human germ cells as well as human infertility, a problem affecting 15% of couples world-wide [20]. Male infertility in particular impacts 7% of the male population (for review see [21]). Notably, male infertility is a risk factor for developing testis germ cell tumor (TGCT) [22]. Testicular cancers are the most frequently diagnosed malignant tumors in young Caucasian males, and their incidence has increased [23], highlighting the importance of the human male germ cell context in studying PUM1- and PUM2-controlled regulation. However, the available germ cell line which has the most in common with human germ cells among germ cell-mimicking cell lines is TCam-2. This cell line originates from human seminoma, a type of TGCT, and represents male germ cells at an early stage of prenatal development [24]. The identification of PUM mRNA targets and PUM-interacting proteins had not been previously studied in human germ cells (which would help establishing the mechanisms underlying functional divergence of PUM1 and PUM2 in these cells) and therefore may help in understanding the reasons behind infertility in humans. To the best of our knowledge, the identification of PUM mRNA targets in germ cells has only been studied in the *C. elegans* model [25]. Therefore, the purpose of this study was to identify and characterize RNA regulons of PUM1 and PUM2 paralogues in TCam-2 cells, clarify whether these regulons are redundant, and if not, discuss potential functional consequences of their divergence in human reproduction. In this study, by RIP-Seq, RNA sequencing, and mass spectrometry (MS), distinct mRNA pools and interacting proteins were identified for PUM1 and PUM2 in human germ cells, thereby enabling understanding of the functional relevance of PUM to fertility.

## 2. Materials and Methods

### 2.1. RNA Immunoprecipitation and Sequencing

For RIP analysis, TCam-2 cells were grown in 37 °C and 5% CO_2_ in Roswell Park Memorial Institute (RPMI, Life Technologies 61870044, Paisley, UK) 1640 medium supplemented with 10% FBS (GE Healthcare HyClone SH30071, Logan, Utah, USA) and 1% penicillin/streptomycin (Lonza EE17-602E, Germany). RIP-Seq experiments with UV cross-linking were performed using the Magna RIP^TM^ RBP Immunoprecipitation Kit (17-700 Merck, Darmstadt, Germany). Briefly, 100 μL of Magnetic A/G beads were coated with 12 μg anti-PUM1 (S-19, sc-65188 Santa Cruz Biotechnology, Santa Cruz, CA, USA), anti-PUM2 (K-14, sc-31535 Santa Cruz Biotechnology, Santa Cruz, CA, USA) antibody or IgG fraction from nonimmunized goat serum (G9759, Sigma Aldrich, Saint Louis, MO, USA) for 45 min at room temperature (RT) in Magna RIP Wash Buffer. TCam-2 cells were washed twice with ice-cold PBS and subjected to UV cross-linking at 254 nm on a HEROLAB CL-1 Cross-linker for 30 s (0.015 J). For one RIP-Seq reaction, 2–3 × 10^6^ cells were lysed in 500 μL of Magna RIP Lysis Buffer for 30 min with rotation at 4 °C. Lysates were centrifuged (10 min at 10,000× *g*), and the supernatant was mixed with precoated beads suspended in washing buffer supplemented with protease and RNase inhibitors. The RIP reaction was held for 3 h at 4 °C on a rotator in a final volume of 1 mL. Then, magnetic beads were washed five times with Magna RIP Washing Buffer, followed by treatment with proteinase K at 55 °C for 30 min. Total RNA was isolated from magnetic beads using a QIAGEN RNeasy Plus Micro Kit according to the manufacturer’s protocol, and RNA quality was checked on an Agilent Bioanalyzer using an RNA 6000 Nano Kit. RNA with a RIN value >7 was used for further steps. cDNA libraries for RNA-Seq analysis were prepared using Illumina TruSeq RNA Sample Prep V2, and subsequent next-generation sequencing was performed on an Illumina HiSeq 4000 platform by Macrogen INC. Sequencing was performed under the following conditions: Paired-End reads were 100 nt long, and >70 million reads/sample were obtained. RIP-Seq with anti-PUM1, PUM2, and IgG (negative control) were performed in triplicate. For TCam-2 transcriptome analysis, total RNA was isolated from 80% confluent 10 cm^2^ dishes using a QIAGEN RNeasy Plus Micro Kit. RNA quality control and RNA-Seq were performed as described above. An mRNA level that was at least 2-fold enriched (with adjusted *p*-value < 0.05) in anti-PUM1/PUM2 co-IP, in comparison to the negative control (co-IP anti-IgG) and to the TCam-2 transcriptome level, was considered to be bound by PUM1 or PUM2.

### 2.2. Western Blot Analysis

To check for PUM1 and PUM2 binding efficiency, SDS lysates from beads after co-IP were resolved on 8% SDS polyacrylamide gels and transferred to nitrocellulose membranes (BioRad). Membranes were blocked with 5% low-fat milk in TBS buffer supplemented with 0.1% Tween 20 (blocking buffer) at RT for 1 h. Membranes were incubated with primary antibodies at 4 °C overnight in blocking buffer. On the next day, membranes were washed four times in TBS buffer with 0.1% Tween 20 and incubated with horseradish peroxidase (HRP)-conjugated secondary antibodies at RT for 1 h in the same buffer. The following antibodies were used: goat anti-PUM1 (1:1000 Santa Cruz Biotechnology #S-19, sc-65188), goat anti-PUM2 (1:250 Santa Cruz Biotechnology #K-14, sc-31535), rabbit anti-actin beta (ACTB) (1:10,000 Sigma Aldrich, A2066), and HRP-linked anti-goat (1:50,000 Santa Cruz Biotechnology #sc-2020), as well as HRP-linked anti-rabbit (1:25000 Sigma Aldrich A0545). Next, membranes were washed twice in TBS buffer with 0.1% Tween 20, and then twice in TBS buffer. ClarityTM ECL Western Blotting Substrate (BioRad, 170—5061, USA) and the ChemiDoc Touch Imaging System (BioRad) were used for signal development and analysis. To check the silencing efficiency of PUM1 and PUM2, SDS lysates were prepared from cells 72 h post-transfection and analyzed in the same way as lysates from beads.

### 2.3. Bioinformatic Analysis of PUM1- and PUM2-Bound mRNAs

The Paired-End sequence reads obtained from the HiSeq4000 platform were trimmed using the Trimmomatic v0.35 tool with the following parameters: ILLUMINACLIP:TruSeq2:PE MINLEN:50, including quality filtration using SLIDINGWINDOW:10:25, MINLEN:50 parameter. Sequence reads that passed quality filters were mapped to the human reference genome (UCSC hg19) using TOPHAT(2.1.0) [26] with default parameters. Then, reads were counted using CUFFLINKS (2.2.1.0), followed by merging replicates with CUFFMERGE and calculating differential gene expression with CUFFDIFF (2.2.1.5) using a *p*-value and false discovery rate (FDR) of <0.05.

For selection of mRNAs potentially bound to PUM1, PUM2, or both, the following criteria were used: (1) only mRNAs enriched in all three replicates; (2) at least 2-fold enrichment in PUM1 or PUM2 IPs, compared to IgG; and (3) at least 2-fold mRNA enrichment in comparison to TCam-2 transcriptome (all enrichment were with *p*-value and FDR < 0.05). To annotate mRNAs bound by PUM to their cell-specific functions and pathways, we performed GO analysis using BiNGO plug-in (version 3.0.3) [27] on Cytoscape platform (version 3.6.1) with functional annotation of biological process and molecular function, searched against TCam-2 cell line gene expression (FPKM > 0.5) background derived from our RNA-Seq. Heatmaps were created using R (version 3.4.4) (R Core Team (2018). R: A language and environment for statistical computing. R Foundation for Statistical Computing, Vienna, Austria. URL: https://www.R-project.org/) and gplots R library. To identify PBEs in the 3′UTR of each PUM1- or PUM2-bound pool of mRNAs, we used the DREME motif discovery tool (v.4.12.0), which enables the identification of short uninterrupted motifs that were enriched in our sequences compared with shuffled sequences. To search for PBE in whole mRNA PUM targets or their 5′UTR, CDS, and 3′UTR, we used FIMO (v.4.12.0), which enables scanning for individual matches for an input motif aligned to individual sequences (with *p*-value < 0.01). Whole mRNAs and their 3′UTR, 5′UTR, and CDS sequences were downloaded from the RefSeq Genes Genomic Sequence database (Table browser: assembly: Feb. 2009(GRCh37/hg19); track: NCBI RefSeq) [28].

### 2.4. siRNA Silencing of PUM Proteins

TCam-2 cells were transfected with siRNA using PUM1 siRNA (sc-62912 Santa Cruz Biotechnology), PUM2 siRNA (sc-44773) containing three different siRNAs for each PUM gene (their sequences are in Table S1) or control siRNA-A (sc-37007) at the final 40 nM concentration using the NEON transfection/electroporation system (Thermo Fisher Scientific). Transfections were performed in Buffer R using 10 µL NEON tips. Subsequently, after transfection, cells were cultured in antibiotic-free RPMI 1640 (Gibco) supplemented with 10% FBS (HyClone) at 37 °C and 5% CO_2_ for 72 h. To measure mostly the post-transcriptional effect of PUM knockdown, transcription was inhibited. To this end, the cells were treated with 5 µg/mL Actinomycin D (A1410 Sigma Aldrich) for 4 h before lysis. Transfection was performed in three biological replicates. RNA isolation was performed using a QIAGEN RNeasy Plus Micro Kit. RNA quality analysis was performed as described above, RNA with RIN > 9 was used for cDNA library preparation, and subsequent sequencing was performed as described above. The knockdown efficiency of each replicate was analyzed by western blot.

### 2.5. Bioinformatic Identification of mRNAs Under Regulation by PUM Proteins

**Figure 1:**
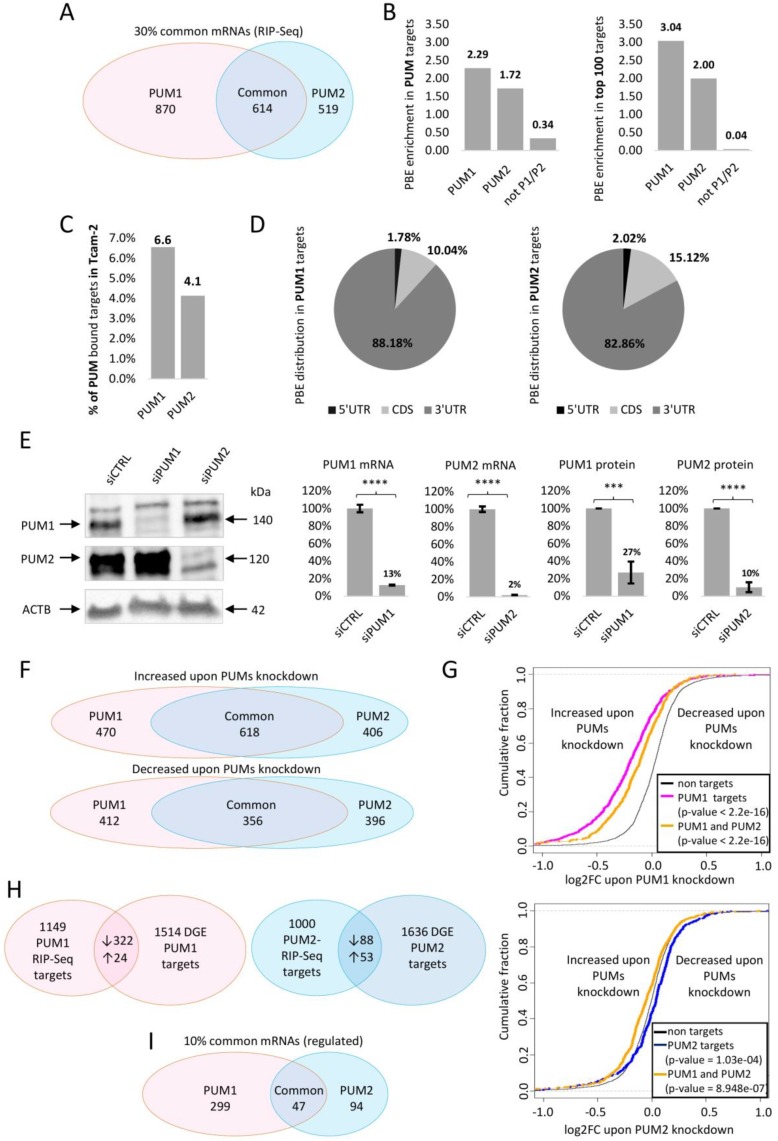
Identification of PUM1- and PUM2-regulated mRNA targets. (**A**) Venn diagram showing the numbers of PUM1-specific, PUM2-specific, and PUM1/PUM2 common mRNA targets from RIP-Seq. (**B**) PUM-binding element (PBE) enrichment in total PUM1- or PUM2-bound mRNAs (left panel) compared to PBE enrichment in the top 100 mRNAs bound to PUM1 or PUM2 (right panel) calculated by using FIMO software, *p* < 0.0001. (**C**) % representation of PUM1- or PUM2-bound mRNAs within the whole TCam-2 mRNA transcriptome. (**D**) Diagrams representing PBE motif distribution within the 5′UTR, CDS, or 3′UTR of PUM1 (left) or PUM2 (right) bound mRNA targets (FIMO analysis with *p* < 0.0001). (**E**) Representative WB showing efficiency of PUM1 and PUM2 siRNA knockdown (left panel) and histograms showing quantitation of mRNA (middle panel) and protein (right panel) knockdown effect from three biological replicates. For quantitative analyses, ACTB was used as a reference for protein analyses; ACTB and GAPDH for mRNA RT-qPCR analyses *** *p* < 0.0005; **** *p* < 0.00005. (**F**) Analysis of mRNAs whose expression was significantly changed upon PUM1 or PUM2 siRNA knockdown. Venn diagram representing the numbers of mRNAs repressed (upper graph) or activated/stabilized (lower graph) by PUM1, PUM2, or both. The Venn diagram represents the number of mRNAs increased (upper graph) or decreased (lower graph) upon siRNA knockdown of PUM1 (pink), PUM2 (blue) or both. (**G**) Cumulative distribution plots of log2FC (fold changes) of all mRNA expression level upon PUM knockdown of targets identified in RIP-Seq PUM1 (upper panel) and PUM2 (lower panel). Changes on the left of the black curve “non targets” control indicate that the targets were repressed, while those on the right of the black curve indicate that the targets were stabilized/activated. (**H**) Venn diagrams showing mRNAs regulated by PUM proteins (as identified by both the RIP-Seq approach and DGE of RNA-Seq upon PUMs knockdown approach); PUM1-regulated (upper panel), PUM2-regulated (lower panel). ↑ activated, ↓ repressed mRNAs (**I**) Venn diagram representing the numbers of mRNAs regulated by PUM1, PUM2, or commonly regulated based on data presented in H.

More than 80 million reads per sample obtained from the Illumina HiSeq 4000 platform were analyzed as described above. If the mRNA level increased by at least 20% (adjusted *p*-value and FDR < 0.05) under 70–90% PUM knockdown compared to negative siRNA control, the mRNA was considered to be under PUM repression. If the mRNA level decreased by at least 20%, it was considered to be significantly activated by PUM. We set the threshold at 20% as sufficient given that these changes were found in three biological replicates (adjusted *p*-value and FDR < 0.05) and the protein silencing efficiency of PUM1 and PUM2 was high, (over 70% and 90%, respectively) (Figure 1E).

Cumulative distribution analysis was performed using log2 fold changes of mRNAs identified in RIP-Seq after PUM1 or PUM2 KD. We used mRNAs not bound in RIP-Seq as controls. A two-sided Kolmogorov–Smitnov test was used to assess statistical significance (using R software version 3.4.4).

### 2.6. RT-qPCR Analysis of mRNA Expression after PUM1 and PUM2 Knockdown

To confirm the targets regulated by PUM1 and PUM2, TCam-2 cells were transfected in three biological replicates with siRNA as described above. RNA from cells was isolated using TRIzol reagent (Gibco) according to the manufacturer’s protocol. Purity and amount of RNA was analyzed by Nanodrop 2000 (ThermoFisher). RNA integrity was determined by Bioanalyzer (Agilent Technologies). Approximately 1 µg of total RNA with RIN > 9 was treated with DNase I (D5307, Sigma-Aldrich) for 20 min at RT and reverse transcribed using the Maxima First-Strand cDNA Synthesis Kit (K1671, ThermoFisher Scientific) according to the manufacturer’s protocol. qPCR was performed on generated cDNA using Jump-Start Taq DNA Polymerase (D4184, Sigma-Aldrich), CFX96 Touch Real-Time PCR Detection System (BioRad) and SYBR Green dye (ThermoFisher Scientific) in three biological replicates with at least five technical replicates of each reaction. The list of primers used for RT-qPCR is shown in Table S2. All changes in mRNA levels upon PUM1 or PUM2 knockdown were normalized to ACTB and GAPDH base on geometric averaging [28]. For all RT-qPCR analyses we have used two-way unpaired *t*-test (α = 0.05) to estimate statistical significance, since all our data was normally distributed, according to a Shapiro–Wilk test (α = 0.05). A *p* value < 0.05 (*) in the *t*-test was considered statistically significant. All our statistical analyses were performed using GraphPad Prism version 8 software.

### 2.7. Mass Spectrometry Analysis after Anti-PUM1 and Anti-PUM2 Immunoprecipitation

Six biological replicates of co-IPs (three performed without RNase A treatment, and another three with 100 mg/mL RNase A) with anti-PUM1, anti-PUM2 antibodies (including anti-IgG negative control) were performed as described above. The same specific antibodies as in RIP were used in MS/co-IP and RIP experiments. MS protein identification analysis was performed by MS Laboratory, IBB PAS, Warsaw. Briefly, proteins were directly digested on the beads and separated by liquid chromatography (LC) followed by MS measurement of peptides and their fragmentation spectra (LC-MS/MS) with a Q Exactive Hybrid Quadrupole-Orbitrap Mass Spectrometer (Thermo Scientific).

The bioinformatic protein identification analysis was performed as described: peak lists obtained from MS/MS spectra were identified using X! Tandem version X! Tandem Vengeance (2015.12.15.2), Andromeda version 1.5.3.4 and MS-GF+ version Beta (v10282). The search was conducted using SearchGUI version 3.2.23 [29].

Protein identification was conducted against a concatenated target/decoy [30] version of the Homo sapiens OX = 9606 (20,316, 99.8%), cRAP (49, 0.2%), the complement of the UniProtKB (version of [2017_06], 20,365 (target) sequences) [31]. The decoy sequences were created by reversing the target sequences in SearchGUI. The identification settings were as follows: trypsin, specific, with a maximum of one missed cleavage of 30.0 ppm as MS1 and 0.1 Da as MS2 tolerances; fixed modifications: carbamidomethylation of C (+57.021464 Da), variable modifications: Oxidation of M (+15.994915 Da), fixed modifications during refinement procedure: carbamidomethylation of C (+57.021464 Da), variable modifications during refinement procedure: acetylation of protein N-term (+42.010565 Da), pyrrolidone from E (−18.010565 Da), pyrrolidone from Q (−17.026549 Da), pyrrolidone from carbamidomethylated C (−17.026549 Da). All specific algorithm settings are listed in the Certificate of Analysis available in the supplementary information.

Peptides and proteins were inferred from the spectrum identification results using PeptideShaker version 1.16.19 [32]. Peptide spectrum matches (PSMs), peptides, and proteins were confirmed at a 1.0% False Discovery Rate (FDR) estimated using the decoy hit distribution. All confirmation thresholds are listed in the Certificate of Analysis available in the supplementary information. Protein identification results for every sample are shown in Table S3. Only proteins inferred with corresponding peptides identified in three independent biological replicates of PUM1 or PUM2 IP and not identified in IgG IP were defined as PUM interactors (Table S4).

### 2.8. Bioinformatic Construction of the PUM RNA Regulon

**Figure 2:**
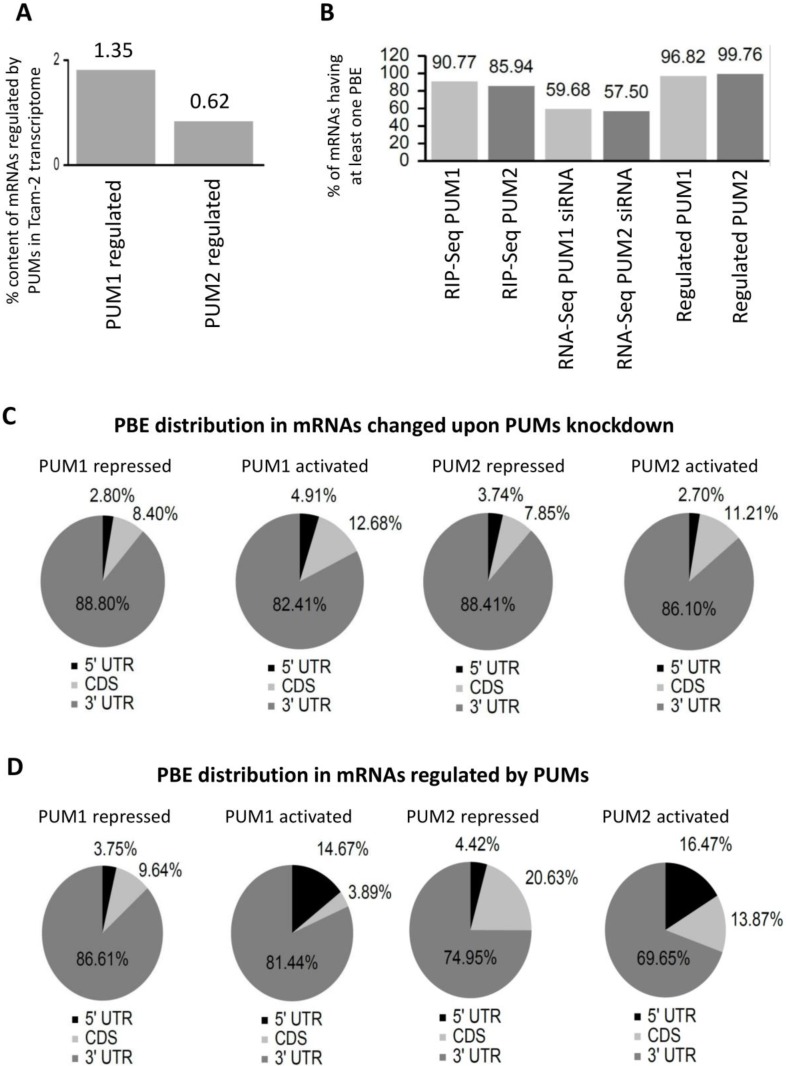
Distribution of PBE motifs in PUM1 and PUM2 targets. (**A**) Content of PUM1- and PUM2-regulated mRNAs in the TCam-2 mRNA transcriptome. (**B**) Content of mRNAs containing at least one PBE in the whole sequence, at three steps of selection: (1) RIP-Seq approach; (2) PUM1 or PUM2 siRNA knockdown; and (3) combined RIP-Seq/RNA-Seq upon PUMs knockdown (regulated). (**C**) PBE distribution in particular regions of mRNAs whose level was changed upon PUM1 (repressed—first, activated—second from the left) and PUM2 (repressed—third, activated—fourth from the left) siRNA knockdown, respectively. (**D**) PBE distribution in particular regions of regulated mRNAs by PUM1 (repressed—first, activated—second from the left) and PUM2 (repressed—third, activated—fourth from the left), respectively.

Binding motifs of putative RBP cofactors of PUM1 and PUM2 were obtained from RBPDB [33] CISBP [34] and POSTAR2 [35] databases (Figure S1A). Motif enrichment analysis was performed on the identified mRNA targets of PUMs (Figure S1, Figure 1B and Figure 2B) by FIMO [36] using a greater-than-average threshold (FIMO analysis with *p*-value < 0.01; mRNAs for GO analysis bigger than average motif enrichment per sequence). mRNA groups regulated by PUM1 or PUM2 with the enrichment of the binding motif putative of RBP cofactors of the respective PUM (Table S5, Figure S1) were determined for each PUM–RBP cofactor pair, in comparison to negative control mRNAs (not bound and not changed under PUM1 and PUM2 silencing). To avoid influence of sequence length, we selected negative sequences, whose average length of 4177 nt (in the range 3000–16,321) was similar to the length of mRNAs regulated by PUM1 (4817 nt, in the range 449–16,862) and PUM2 (5442 nt, in the range 412–16,862). Given that the average size of negative sequences was slightly shorter compared to PUM-regulated targets, we used a coefficient for length correction. GO analysis of PUM-RBP cofactor-regulated mRNAs was performed using ClueGO version 2.5.2 [37]. GO term selection was performed by using only experimentally validated annotation as evidence code, using the selection parameters of minimum genes annotated to term: 3, minimum percentage of genes: 4%. Annotated biological processes with adjusted *p*-values after Bonferroni correction ≤ 0.05 were considered significant. GO analysis results are shown in Table S6. Visualization of the regulon was performed using Cytoscape platform version 3.6.1.

### 2.9. Quantification and Statistical Analysis

Statistical analyses were performed as indicated in the above Materials and Methods subsections. To perform them, we used GraphPad Prism version 8 software or R (version 3.4.4). For the rest, statistics included in pipelines and software mentioned above were used.

In the supplementary figures, Pearson correlation of our TCam-2 and Irie and coworkers’ [38] TCam-2 transcriptome was calculated and visualised using Galaxy platform: deepTools plotCorrelation as described in [39]. Principal Component Analysis (PCA) plots were obtained using Galaxy platform: deepTools plotPCA as described in Jarmoskaite I. et al., 2019 [39].

## 3. Results

### 3.1. Identification of PUM1 and PUM2 mRNA Targets by RIP-Seq

As the first step, to identify human PUM1- and PUM2-bound mRNAs in the TCam-2 cell line, we performed RNA immunoprecipitation (RIP) followed by RNA-Seq. Specificity of antibodies to PUM1 or PUM2 *N*-terminal regions was verified by excluding cross-reactions (Figure S2). Prior to RNA-Seq analysis of PUM-bound mRNAs, we performed RNA-Seq analysis of the TCam-2 transcriptome to use as the second reference in RIP-Seq experiments. The RNA-Seq data was in close agreement with the published TCam-2 transcriptome, with a Pearson correlation R2 value of 0.957 (Figure S3) [38]. PCA plot of each biological replicate of RIP anti-IGG, PUM1 and PUM2, and the transcriptome of TCam-2 is shown on Table S4A.

The RIP-Seq approach allowed us to identify 1484 and 1133 polyadenylated RNAs that were significantly enriched (at least two-fold) in the anti-PUM1 and anti-PUM2 IPs, respectively, compared to the levels found in IgG IPs and the TCam-2 transcriptome (Table S7). Of these, 870 mRNAs were found to specifically bind to PUM1 alone, 519 to PUM2 alone, and 614 (30%) were bound to both PUM1 and PUM2 (Figure 1A, Table S7).

Although it was previously established that each human PUM paralogue specifically recognizes the PBE motif UGUANAUA [10], the PBE motif UGUAHAUW (H stands for A, C, or U, while W stands for A or U) was found in a recent study to be more accurate for PUM1 and PUM2 [40]. Therefore, in this study, mRNAs identified by RIP-Seq were screened for the presence of the UGUAHAUW motif. We found that on average, PUM1-bound mRNAs contained 2.29 UGUAHAUW motifs/sequences, while PUM2-bound mRNAs contained 1.72 (Figure 1B left panel). The same analysis, when performed for the 100 most enriched PUM1 and PUM2 targets, revealed that the motif frequency was higher, with PBE content of 3.04 and 2.00 for PUM1 and PUM2, respectively (Figure 1B right panel). In contrast, in nonspecifically bound mRNAs (those present in immunoprecipitates, but that were not significantly enriched (<2×) in comparison to nonimmune serum and the TCam-2 transcriptome), PBE motif occurrence was significantly lower (0.34/sequence for all RIP-Seq identified and 0.04/sequence for the top 100 targets with a higher occurrence in anti-IgG than in anti-PUM immunoprecipitates) (Figure 1B). We observed that altogether ~6.6% of the TCam-2 transcriptome presented PUM1-bound mRNAs, and ~4.1% presented PUM2-bound mRNAs and almost all of them contained PBEs (Figure 1C and Figure 2B first two bars). We next checked for PBE motif localization within each PUM1 and PUM2 target mRNA to determine the percentage of target mRNAs that harbored PBEs in the 3′UTR or in other locations. We found that PBEs were mostly located in the 3′UTR (88% and 83% for PUM1 and PUM2, respectively), less frequently in CDS (10% and 15% for PUM1 and PUM2, respectively), and rarely in the 5′UTR (1.8% and 2.0% for PUM1 and PUM2, respectively) (Figure 1D).

To address whether PUM1- and PUM2-bound mRNAs selected by the RIP-Seq approach represented similar or different cellular functions, we performed Gene Ontology analysis (Tables S5 and S8). Since we obtained similar numbers of mRNAs bound to PUM1 and PUM2 (1484 and 1133, respectively), we were able to use the BiNGO plug-in in the Cytoscape platform [27] to compare the biological processes and molecular functions of these two groups in an unbiased manner using our TCam-2 transcriptome as a background. We found that while the majority of targets represented overlapping biological processes (BP) and molecular functions (MF), some were related only to PUM1 targets, e.g., chromosome organization, positive regulation of transcription, chromatin modification (BP from Table S5A), transcription regulator activity, GTPase activator activity, and protein binding (MF from Table S5B). Some other functions were related only to PUM2 targets, e.g., cell cycle, organelle organization, M phase (BP from Table S5A), motor activity, helicase activity, and cytoskeletal protein binding (MF from Table S5B).

### 3.2. Differential Gene Expression Analysis upon PUM1 or PUM2 siRNA Knockdown

Since mRNA binding alone does not imply regulation by RBPs, and PUM1-repressed mRNAs have been reported to undergo degradation [41] or activation [40], we next sought to identify those mRNAs whose expression was modified upon PUM1 or PUM2 gene knockdown. At 72 h after TCam-2 cell transfection with PUM1 or PUM2 siRNA (Figure 1E), when silencing efficiency was the highest for both paralogues, RNA was isolated and RNA-Seq analysis was conducted. We refer to this approach as RNA-Seq upon PUMs knockdown from hereon. PCA plots showing each biological replicate of siRNA KD of PUM1, PUM2, and control is shown in Table S4B. Our analysis revealed 1088 genes with higher expression and 768 genes with lower expression upon PUM1 knockdown, and 1024 genes with higher expression and 752 genes with lower expression upon PUM2 gene knockdown (with the adjusted *p* and FDR < 0.05) (Figure 1F, Table S9). RNA-Seq analysis revealed that among these, 470 genes were specifically repressed and 412 genes were specifically activated by PUM1 alone, 406 genes were specifically repressed and 396 genes were specifically activated by PUM2 alone, 618 genes were repressed by both PUM1 and PUM2, and 356 genes were activated by both PUM1 and PUM2 (Figure 1F, Table S9).

To check whether expression of RIP-Seq-identified targets is significantly changed upon PUM1 or PUM2 knockdown, cumulative distribution analysis was performed. It showed significant repression of PUM1 and PUM1/PUM2 common RIP-Seq-identified targets, in comparison to nontargets upon PUM1 knockdown (Figure 1G left panel, pink and orange lines, in comparison to the black line). Upon PUM2 knockdown PUM2 RIP-Seq-identified targets show significant activation, while PUM1/PUM2 common targets significant repression (Figure 1G right panel, blue and orange lines, in comparison to the black line). This analysis confirms validity of our approach.

Additionally, Gene Ontology analysis of mRNAs up- and downregulated upon PUM1 and/or PUM2 siRNA knockdown was performed and is presented in Table S6. This analysis shows that most of the biological processes and molecular functions of these mRNAs overlap.

### 3.3. Selection of PUM1- and PUM2-Regulated mRNA Targets Based on RIP-Seq and PUMs Knockdown Analysis

For the identification of PUM1- and PUM2-regulated mRNA targets, we used the following two selection criteria: #1 binding to PUM1 or PUM2 as detected by RIP-Seq (genes listed in Table S7) and #2 down- or upregulation of mRNA levels upon PUM1 or PUM2 siRNA knockdown and RNA-Seq (genes listed in Table S9). The simultaneous use of both criteria provided us with 346 (322 repressed and 24 activated) PUM1-regulated (Figure 1H upper panel, Table S10) and 141 (88 repressed and 53 activated) PUM2-regulated (Figure 1H lower panel, Table S10) mRNAs. Additionally, by using these two criteria, we found that the number of mRNAs shared by PUM1 and PUM2 was reduced to 10% (47 common mRNAs) (Figure 1I) compared to the 30% seen by RIP-Seq-based selection (Figure 1A). The mRNAs regulated by both PUM1 and PUM2 represented only 1.35% and 0.62% of the TCam-2 transcriptome, respectively (Figure 2A), compared to 6.56% and 4.14% PUM1- or PUM2-bound mRNAs (Figure 1C). To further validate the mRNA pools that we considered to be regulated by PUM1 and PUM2 (Figure 1H,I), we analyzed their PBE-motif content. We found that the number of mRNAs containing at least one PBE reached nearly 100% (96.82% for PUM1 and 99.76% for PUM2) (Figure 2B). This is significantly higher than the PBE content in RIP-Seq- or PUMs knockdown-identified mRNAs (RIP-Seq PUM1 90.77%; RIP-Seq PUM2 85.94%; PUM1 knockdown 59.68%; PUM2 knockdown 57.50%). This result additionally validated our approach. PBE motif distribution in regions of PUM mRNA targets with significant expression changes upon knockdown is shown in Figure 2C. We found that in the case of mRNAs under positive regulation by PUM1 and/or PUM2, PBE motif frequency in the 5′UTR was almost four times higher than in mRNAs pools under PUM1 and/or PUM2 repression (Figure 2D), suggesting that the 5′UTR sequence is more frequently used in the case of mRNAs activated/stabilized by PUMs than in repressed mRNAs. Gene ontology analysis revealed that most of the biological processes and molecular functions of mRNAs regulated by PUM1 and PUM2 are different. While PUM1-regulated targets are involved, e.g., in mitotic cell cycle checkpoint, regulation of transcription, developmental process, chromosome localization (BP from Table S7A), small GTPase regulator activity, and protein binding (MF from Table S7B), PUM2 regulated targets are involved, e.g., in negative regulation of cell division (BP from Table S7A), signal transducer activity, MAP kinase activity, kinase activity, and transferase activity (MF from Table S7B). GO analysis also revealed a minority of molecular functions involving both PUM1- and PUM2-regulated mRNAs, e.g., nucleoside-triphosphatase regulator activity, enzyme regulator activity, and nucleoside binding (MF from Table S7B).

### 3.4. Global Profiling Reveals Many Putative Protein Cofactors Interacting with PUM1 and PUM2 to be RBPs

**Figure 3:**
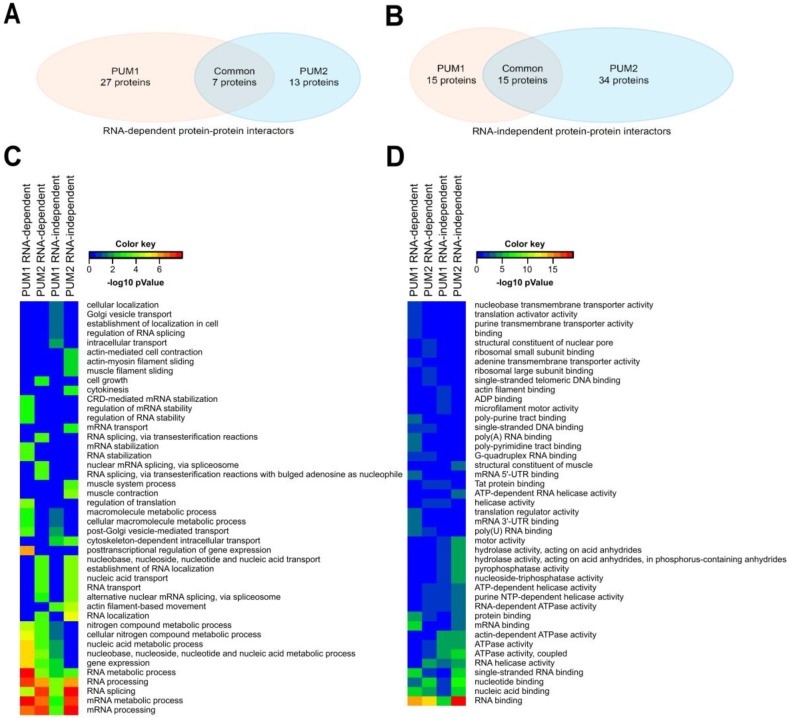
Identification of proteins interacting with PUM1, PUM2, or both, identified by coimmunoprecipitation (co-IP) and mass spectrometry (MS). (**A**) Proteins interacting with PUM1, PUM2 or both via RNA (co-IP without RNase A treatment). (**B**) Proteins interacting with PUM1, PUM2 or both, independently of RNA (co-IP with RNase A treatment). (**C**) Heatmap of BiNGO analysis of TOP20 Biological Processes of PUM1- or PUM2-interacting proteins in RNA dependent or independent manner. **(D)** Heatmap of BiNGO analysis of TOP20 Molecular Functions of PUM1- or PUM2-interacting proteins in RNA dependent or independent manners. The detailed results of the GO analysis are presented in Table S8.

Our result showing that PUM1 and PUM2 share only ~10% of their mRNA targets (Figure 1I) was surprising given that PUM1 and PUM2 recognize the same UGUAHAUW motif [40] and demonstrated remarkably high similarity in binding potential across 12,285 sequences, as illustrated by quantitative analysis of RNA on a massively parallel array (RNA-MaP) [42]. Since N-terminal regions are known to be structurally divergent [9], they might function differently in PUM1 and PUM2, for example, by binding different sets of protein cofactors. We hypothesized that PUM1 and PUM2 discriminate between specific mRNA targets in vivo by interacting with unique protein cofactors. To test that hypothesis, we performed anti-PUM1 and anti-PUM2 co-IP experiments in the presence and absence of RNAse A, followed by mass spectrometry (MS), to identify PUM1- and PUM2-interacting proteins in TCam-2 cells in an RNA-dependent and -independent manner (Table S4). We identified 52 PUM1-interacting and 55 PUM2-interacting proteins, 25 of which were common to both (Table S4). Importantly, we identified 27 PUM1-, 13 PUM2-, and seven PUM1/PUM2-interacting proteins, all of which required the presence of RNA for binding (Figure 3A) and are known RBPs. We also identified 15 PUM1-, 34 PUM2-, and 15 PUM1/PUM2-interacting proteins that interacted in an RNA-independent manner (Figure 3B). Finally, 28 of these PUM-interacting proteins were identified both in the presence and absence of RNA (Table S4).

Moreover, gene ontology analysis of PUM1- and PUM2-interacting proteins revealed that the majority of them are functionally involved in RNA binding, regulation, and processing (as shown at the bottom of Figure 3C,D); however, there are some significant differences between paralogs. Exclusively, PUM1 cofactors (such as CLTLC and CLTA) are involved in post-Golgi vesicle-mediated transport. PUM2 cofactors instead are much more involved in RNA splicing, for example, SFPQ, SRSF1, SRSF7, HNRNPM, and HNRNPA2B1. PUM2 cofactors are more involved in RNA localization (HNRNPA2B1 and HNRNPM—nuclear localization, G3BP1, G3BP2—localization in P-bodies). Taken together, these results support our hypothesis that PUM1 and PUM2 regulate distinct pools of mRNAs by interacting with divergent proteins.

### 3.5. PUM1 and PUM2 Form Separate Regulons in TCam-2 Cells

Interestingly, 54 of the 82 PUM1- and PUM2-interacting proteins identified in this study are known RBPs, and for 26 of them, a specific RNA-binding motif has already been established using Photoactivatable Ribonucleoside-Enhanced Crosslinking and Immunoprecipitation (PAR-CLIP), CLIP-Seq or RNA competing methods [34,43] (Tables S1A and S4). To check whether PUM1- and PUM2-interacting RBPs could potentially cooperate with PUM in the selection of specific mRNA targets for regulation, we first checked whether the binding motifs corresponding to these RBPs co-occur with PBEs in PUM1- and PUM2-regulated mRNA targets. To this end, we performed an analysis of binding motif enrichment for 10 PUM1-specific and eight PUM2-specific RBPs in PUM1 or PUM2-regulated mRNA targets, respectively. We found that RNA binding motifs for six out of ten PUM1-interacting RBPs—IGF2BP3, YBX1, NUDT21, IGF2BP1, PABPC4, and CPSF7, but not FUS, LIN28A, HNRNPK, and CPSF6—were highly enriched in PUM1 regulated mRNAs (Table S1B). In the case of PUM2, we found that RNA binding motifs for six out of eight PUM2-bound RBPs – PTBP1, G3BP2, G3BP1, HNRNPF, FMR1, and SRSF7, but not HNRNPA2B1 and SRSF1—were highly enriched in PUM2-regulated mRNAs (Table S1C). Additionally, we also performed analysis of motif enrichment for seven common PUM1- and PUM2-interacting RBPs in mRNAs regulated by both PUM1 and PUM2 and found that RNA binding motifs for almost all common PUM1 and PUM2 interacting RBPs—SFPQ, FXR1, FXR2, HNRNPA1, MATR3, and PABPC1, but not NCL—were highly enriched in both PUM1- and PUM2-regulated mRNAs (Table S1D). RBPMS and MBNL1 RBPs were used as the negative controls in that analysis. These controls were selected because first, we did not identify them as PUM-binding in our anti-PUM CoIP/MS analysis. Second, they were not identified as PUM-binding RBPs by other authors. Third, they contain a specific RNA binding motif that significantly differs from a random nucleotide distribution [2]. Motif enrichment was evaluated relative to motif enrichment in nonregulated mRNAs set as 1 (Table S1B–D dashed lines). The high enrichment of RBP motifs that we found is an additional indication for these RBPs to be potential putative PUM1 or PUM2 protein cofactors in the regulation of their mRNA targets.

**Figure 4:**
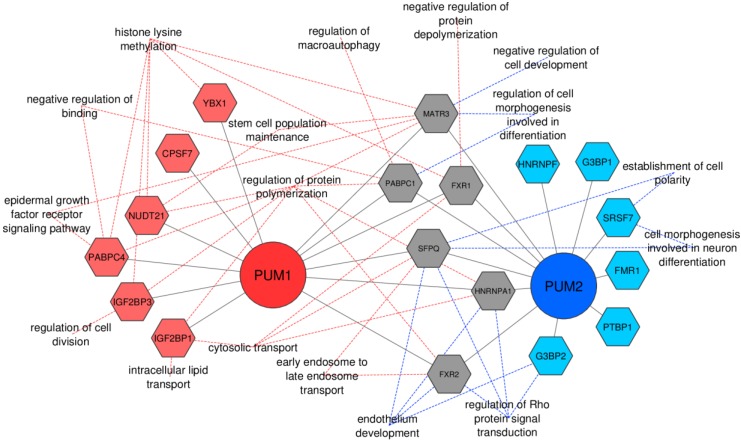
Model of PUM1 and PUM2 RNA regulons in TCam-2 cells. PUM1 and PUM2 are represented by red and blue circles, respectively, while PUM1-, PUM2-, and PUM1/PUM2-interacting RBPs are represented by red, blue, or grey diamonds, respectively. Continuous lines represent interactions of PUM1 and PUM2 with putative RBP cofactors. Dashed lines represent functions (biological processes from ClueGOanalysis) of groups of mRNAs regulated by PUM1 (dashed red lines) and PUM2 (dashed blue lines) with high enrichment (above threshold) of binding motifs for particular RBPs in mRNAs regulated by PUM1 or PUM2.

To further explore potential functional specificities of PUM1 and PUM2, we combined the RIP-Seq (Figure 1A), RNA-Seq upon PUMs knockdown (Figure 1E–G), co-IP/MS (Figure 3), and RNA binding protein motif enrichment (Table S1), and performed a strict GO analysis by using only experimentally validated data as GO evidence code (Table S6). This combined analysis was based on the assumption that an mRNA containing a binding motif for a specific PUM protein cofactor, the frequency of which is significantly higher (above average, and not enriched in negative control (Table S6) than in the control mRNA dataset, is coregulated by that protein cofactor (Figure 4) (Table S5). The main findings are as follows. First, there are separate PUM1 and PUM2 RNA regulons. Second, PUM1 and PUM2 may cooperate with varied components to regulate different pathways. As examples, PUM1 and IGF2BP1 may coregulate mRNA subpools involved in intracellular lipid transport; PUM1 together with PABPC4 and MATR3 may coregulate mRNAs involved in the epidermal growth factor receptor signalling pathway, and PUM1 together with PABPC1 and PABPC4 may coregulate mRNAs involved in negative regulation of binding. On the other hand, PUM2, together with SFPQ and SRSF7, may coregulate establishment of cell polarity and cell morphogenesis involved in neuron differentiation; PUM2, together with G3BP2, HNRNPA1, FXR2, and SFPQ, may coregulate mRNAs involved in regulation of Rho protein signal transduction. PUM2 and MATR3 may coregulate mRNAs involved negative regulation of cell development; PUM1, together with IGF2BP3, may coregulate mRNAs involved in regulation of cell division; PUM1, together with PABPC4, IGF2BP3, YBX1, NUDT21, FXR1, and MATR3, may coregulate mRNAs involved in histone lysine methylation. Third, although PUM1 and PUM2 form separate regulons, they may cooperate in the regulation of some common mRNA targets, which are involved in the same biological processes (Figure 4).

### 3.6. Several mRNAs Highly Expressed in TCam-2 Cells Compared to Somatic Gonadal Tissue are Regulated by PUM Proteins

**Figure 5:**
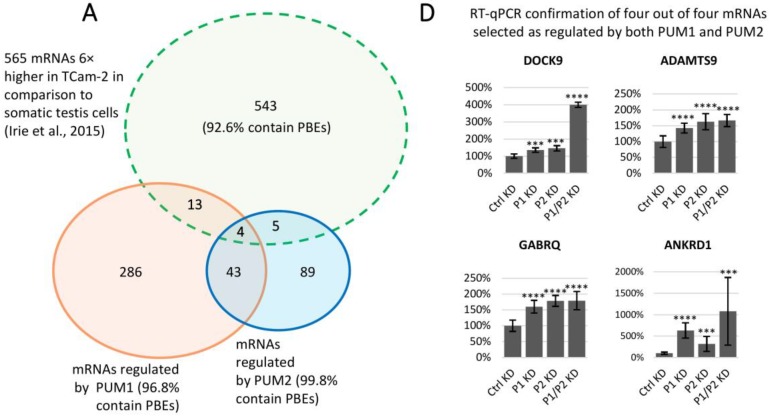

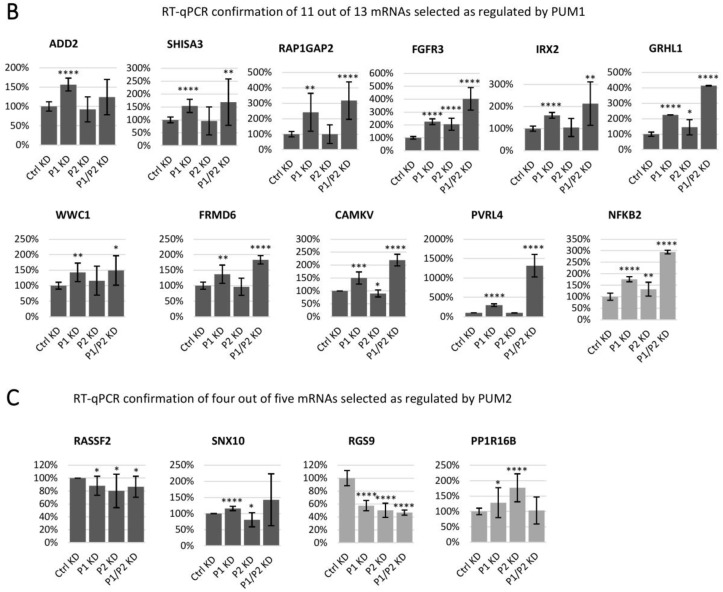
RT-qPCR analysis of mRNAs regulated by PUM proteins that are enriched in TCam-2 cells compared to human somatic testis tissue. (**A**) Venn diagram showing 22 mRNAs identified as regulated by PUM1, PUM2, or both, selected from a pool of 565 mRNAs (92.6% of which was found to have PBE) found to be at least 6-fold enriched in TCam-2 cells, compared to somatic testis cells, as published by [38]. (**B**) RT-qPCR confirmation of 11 out of 13 mRNAs selected as regulated by PUM1. (**C**) RT-qPCR confirmation of four out of five mRNAs selected as regulated by PUM2. **(D)** RT-qPCR confirmation of four out of four mRNAs selected as regulated by both PUM1 and PUM2. Dark grey histograms highlight mRNAs containing at least one classic PBE motif (UGUAHAUW), light grey histograms indicate mRNAs that do not contain any classic PBE, but a degenerated one with UGUA core. For all three repetitions of RT-qPCR, *ACTB* and *GAPDH* served as references. *p < 0.05; **p < 0.005; ***p < 0.0005; ****p < 0.00005.

Of the many cellular processes regulated by PUM proteins, those involved in germ cell development are of particular interest due to implications to understand infertility in humans (for review, see [4]). Therefore, we determined which genes highly and selectively expressed in germ cells are under PUM1 and/or PUM2 regulation. To this end, we first identified genes whose expression in germ cells was at least six times higher than in human testis somatic gonadal tissue by comparing TCam-2 transcriptomic data with the previously published transcriptome of human testis somatic gonadal tissue [38]. This comparison identified 565 genes highly expressed in TCam-2 (Figure 5A), including 22 regulated by PUM proteins. Specifically, we identified 13 genes regulated by PUM1 alone, five genes regulated by PUM2 alone, and four by both.

To confirm that these 22 selected genes are under PUM1 and/or PUM2 regulation, we measured their expression by RT-qPCR in TCam-2 cells untreated or treated with PUM1 siRNA, PUM2 siRNA or both PUM1 and PUM2 siRNA. By this approach, we validated 19 mRNAs to be regulated by PUM proteins (Figure 5 and Table S11) of which 11 were regulated by PUM1 (seven by PUM1 alone, three more significantly by PUM1 than by PUM2, while one was repressed by PUM1 and activated by PUM2) (Figure 5B). Another four mRNAs were regulated by PUM2 (three more significantly regulated by PUM2 than by PUM1, and one was activated by PUM2 and repressed by PUM1) (Figure 5C). The four remaining mRNAs were regulated by both PUM1 and PUM2 (Figure 5D).

Interestingly, regulation of nine among eleven PUM1 and one among four PUM2 targets seems to be the strongest upon PUM1/PUM2 double knockdown when compared to control siRNA knockdown (Figure 5B,C, respectively). The seven genes regulated by PUM1 alone encode SHISA3, RAP1GAP2, IRX2, WWC1, FRMD6, PVRL4, and ADD2 (Table S11). Six of the eleven PUM1-regulated genes are associated with failure or cancer of the male as well as the female reproductive system. The four genes regulated by PUM2 encode RASSF2, SNX10, RGS9, and PPP1R16B (Table S11), which function in prostate tumor suppression, osteoporosis malignancy, nervous system development, and endothelial cell proliferation, respectively. The four genes regulated by both PUM1 and PUM2 encode DOCK9, ADAMTS9, GABRQ, and ANKRD1, which are involved in filopodia formation in cervical cancer, cell cycle regulation and ovary cancer progression, promotion of cell proliferation in hepatocellular carcinoma, and downregulation of apoptosis, respectively. Three of these genes are associated with cancer of the reproductive system (Table S11).

We found that the majority of those PUM1- and PUM2-regulated targets are involved in cancer (16 among 19), including 10 in cancer of the male or female reproductive system. PUM2-regulated PPP1R16B is functionally unique because it is the only PUM target that regulates phosphorylation. RT-qPCR validation of such a high proportion of genes indicates that our approach for PUM target identification was accurate.

As expected, 16 of the 19 validated genes contained at least one classic UGUAHAUW motif (Figure 5B–D dark grey bars), and all of them contain motifs with single nucleotide substitution in last five positions in comparison to classic PBE.

### 3.7. Genes Associated with Male Infertility in Humans and/or Mice are Regulated by PUM Proteins

**Figure 6:**
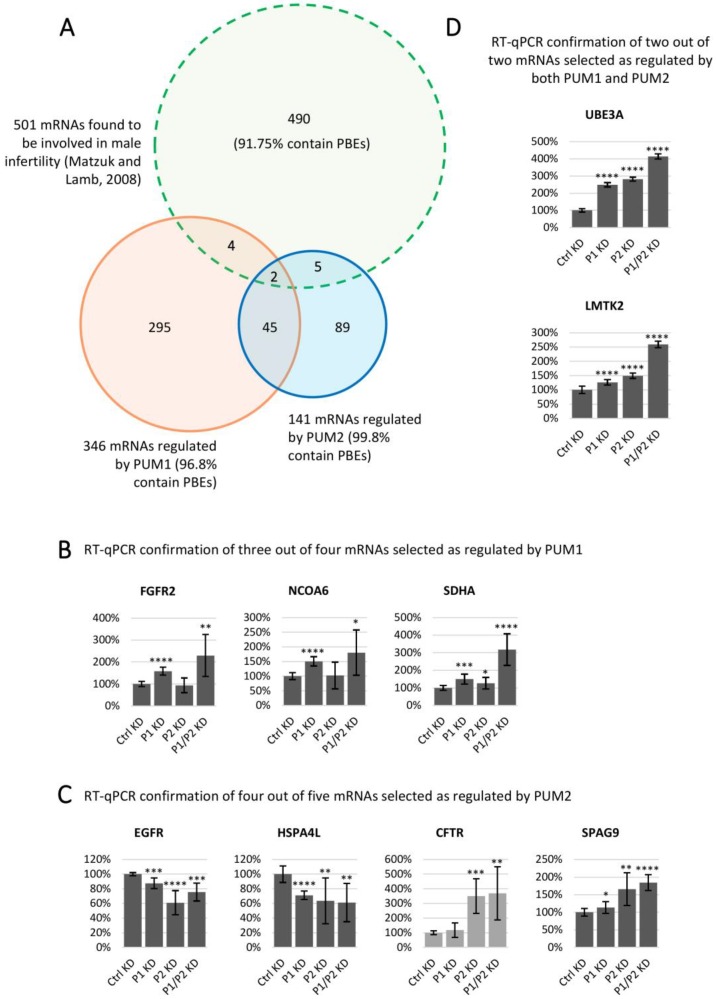
RT-qPCR analysis of mRNAs regulated by PUM1 and/or PUM2, which are important for human and/or mouse male fertility. (**A**) Venn diagram showing 11 mRNAs identified as regulated by PUM1, PUM2, or both PUM1/PUM2 from a pool of 501 mRNAs (91.75% of which was found to have PBE) found to be involved in male infertility [44]. (**B**) RT-qPCR confirmation of three out of four mRNAs selected as regulated by PUM1. (**C**) RT-qPCR confirmation of four out of five mRNAs selected as regulated by PUM2. (**D**) RT-qPCR confirmation of two out of two mRNAs selected as regulated by PUM1 and PUM2. Dark grey histograms indicate mRNAs with at least one classic PBE motif (UGUAHAUW), light grey histograms indicate mRNAs that do not contain any classic PBE, but a degenerated one with UGUA core. For all three biological repetitions of RT-qPCR, *ACTB* and *GAPDH* served as references. * *p* < 0.05; ** *p* < 0.005; *** *p* < 0.0005; **** *p* < 0.00005.

We next sought to determine whether PUM-regulated genes in the male germ cell line, TCam-2, are associated with male infertility. To this end, from a list of 501 genes, which have been validated to cause infertility when mutated or disrupted in humans and/or mice [44], we selected 11 genes that were also found in this study to be regulated by PUMs (Figure 6A and Table S11). Upon PUM1 and PUM2 gene siRNA knockdown followed by RT-qPCR for these 11 genes, we validated nine of them as PUM targets. Of these nine genes, two were regulated by PUM1 (Figure 6B), two were regulated by PUM2 alone (Figure 6C), and five were regulated by both PUM1 and PUM2 (Figure 6D). In the case of seven PUM1- or PUM2-regulated targets, the effects were the strongest upon PUM1/PUM2 double knockdown (Figure 6B,C). We found that eight out of nine of the PUM1 and PUM2 confirmed targets contained at least one classic UGUAHAUW motif (Figure 6B–D), and all of them also contain motifs with single nucleotide substitution in the last five positions in comparison to PBE. Dysfunction of all of the above nine genes have been reported to be associated with male infertility or testis cancer (Table S11).

PUM1 and PUM2 may cooperate with varied components to regulate different targets related to reproduction including infertility, such as NFKB2, FGFR3, FGFR2, and NCOA6, which are PUM1 targets (mRNAs are shown in Table S10 functions and citations are in Table S11). NFKB2, which is involved in aberrant activation of androgen receptor in prostate cancer cells, might be coregulated by FXR2 and HNRNPA1 proteins based on our motif enrichment analysis (Table S1). Interestingly it is a target of FXR2 in CLIP experiments from other cell lines based on a POSTAR2 [35] database search. FGFR3, which has a role in testis tumor development might be coregulated by a different set, SFPQ, FXR2, and HNRNPA1, and all three bind it based on POSTAR2. FGFR2, whose mutations were associated with hypospadias, might be coregulated by SFPQ and HNRNPA1. The last one is known to bind FGFR2. Finally, NCOA6, which is involved in embryo implantation, might be coregulated by a large group of the following proteins: SFPQ, FXR1, FXR2, HNRNPA1, MATR3, PABPC1, IGFBP3, YBX1, NUDT21, IGF2BP1, PABPC4, and CPSF7 (HNRNPA1, IGF2BP1, IGF2BP3, NUDT21, and CPSF7 were shown to bind NCOA6 in several CLIP experiments). The same rule is observed in the case of PUM2 targets involved in reproduction—RASSF2, EGFR, CFTR, and SPAG9 (Tables S10 and S11). RASSF2, which is a tumor-suppressor in the prostate cancer mouse model, might be coregulated by FXR1, FXR2, HNRNPA1, PTBP1, G3BP1, HNRNPF, and SRSF7. PTBP1 was previously found to bind RASSF2 in CLIP. EGFR, the signalling dysfunction of which was associated with human male infertility, might be coregulated by FXR1, FXR2, and HNRNPA1 (all of these three bind EGFR from CLIP experiments), G3BP2, G3BP1, FMR1, and SRSF7. CFTR, mutations of which are associated with male infertility, might be coregulated by FXR1, HNRNPA1, MATR3, PABPC1, G3BP2, FMR1, and SRSF7. Finally, SPAG9, which stimulates prostate cancer cell proliferation, might be coregulated by FXR1, FXR2, HNRNPA1, MATR3, PABPC1, PTBP1, G3BP2, G3BP1, HNRNPF, FMR1, and SRSF7 (FMR1, FXR1, FXR2, HNRNPA1, and PTBP1 bind SPAG9 in several CLIP experiments). CLIP experiments (PAR-CLIP, HITS-CLIP, iCLIP and eCLIP) mentioned in this paragraph were performed in a few cell lines (including HEK293, K562, HepG2) [35]. Such data is not available for TCam-2.

## 4. Discussion

Considering the high structural similarity of PUM1 and PUM2, it is still unresolved whether they form separate RNA regulons in mammals. Here, for the first time, by combining RIP-Seq and RNA-Seq upon PUMs knockdown data, together with co-IP LC/MS identification of putative protein cofactors, RNA binding motif enrichment, and GO analysis (for the first time each group of data originating from the same cells—TCam-2 cells), we obtained a model of partially divergent putative PUM1 and PUM2 RNA regulons (Figure 4). They are reminiscent of previously proposed regulons [8]. Importantly, a global PUM-dependent gene expression regulation was not studied in germ cells, except *C. elegans* [24,25,45].

We found a much higher average representation of PBE-containing mRNAs that were selected as regulated by PUM1 and PUM2 based on combined analysis of RIP-Seq and PUM RNA-Seq upon PUMs knockdown (96.8 and 99.8%, respectively), than in targets selected based on RIP-Seq (90.8 and 85.9%, respectively) or RNA-Seq alone (59.68, 57.50%, respectively), which validates our approach (Figure 2B). It is important to note that several of our RIP-Seq identified targets overlapped with mRNAs previously identified in HeLa [10] and HEK293 [40,46] cells, confirming our results. However, it is also important to bear in mind that PUM-mediated activation or repression, or lack of PUM regulation, may be cell-type-specific [47]. Therefore, we can expect only a partial target overlap when PUM targets from different types of cells are compared.

It is important to note that among PUM-regulated mRNAs, there are also a small number of targets with no PBE (approximately 3% PUM1- and below 1% PUM2-regulated). As mentioned above, PUM proteins may recognize motifs slightly different to the canonical UGUAHAUW [12,42]. Such variant motifs were not evaluated in this study; therefore, putative mRNA targets carrying such motifs were overlooked. It is also important to emphasize that in our approach, PUM-regulated mRNAs whose level remained unchanged (did not undergo degradation or stabilization) were overlooked. PUM2-regulated and not PUM1-regulated mRNA repression with no degradation but rather storage in P-bodies was recently suggested to be quite common in human HEK293 cells [48]. Our result showing a lower number of PUM2 compared to PUM1-regulated targets is in line with that finding.

We found that in mRNAs positively regulated (activated/stabilized) by PUM1 or PUM2, PBE motifs were significantly more frequent in the 5′UTR (14.67% for PUM1 and 16.47% for PUM2) than in mRNAs negatively (repressed) by PUM1 and PUM2 (3.75 and 4.42%, respectively). However, this was not reported in studies on HEK293 cells [40]. Although this observation requires further research, it may suggest that activation of these mRNAs by PUM proteins requires PBE localization in the vicinity of some 5′UTR translational signals.

Interestingly, by using the RIP-Seq approach we identified 30% of PUM1/PUM2-bound common targets. However, the combination of RIP-Seq with siRNA knockdown to identify regulated targets resulted in a decrease of common targets to 10%. We propose that this difference is due to the involvement of distinct regulatory factors for each PUM paralogue. It is worth emphasizing that we identified such regulatory factors—putative PUM-interacting protein cofactors which control different aspects of RNA metabolism (stability, localization, transport, splicing and expression regulation), whose interaction was RNA-mediated as well as protein cofactors whose interaction was RNA-independent. A substantial number of protein cofactors were PUM1- or PUM2-specific in both groups. The first group of RNA-dependent protein cofactors contains only RBPs, which was expected, and confirms our experiments as well as the analysis performed. However, RBPs were also significantly enriched in the second group representing RNA-independent protein–protein interactions. Such RBPs are likely to contain protein–protein interacting domains that bind PUM, as well as RNA-interacting domains that bind RNA. Finally, interactors with no RNA-binding domains might be important for the stabilization of ribonucleoprotein complexes, which are formed upon PUM protein binding specific mRNA targets.

Among the identified PUM putative protein interactors, we found five previously reported human PUM binding proteins, which confirms our results. MATR3 and SEC16A were previously identified in a high-throughput proteomic study in HeLa cells [49]. Another one is G3BP1, which is a stress granule assembly factor [50]. The next one is the fragile X mental retardation protein (FMR1) and its autosomal homologous proteins, FXR1 and FXR2. FMR1 was previously shown to colocalize with PUM2 in rat neuron stress granules [51]. More recently, Zhang and coworkers reported that FMR1 interacts with PUM in the murine brain in an RNA-dependent manner [52]. In our study, FMR1 proteins were identified as both PUM RNA-dependent and independent interactors. Interactions with G3BP1 and FMR1 may suggest that PUM paralogues are components of stress granules not only in mammalian neurons [51], but also in human germ cells. The presence of both PUM paralogues in stress granules suggests their involvement in RNA storage. Interestingly, PUM proteins were also found in P-bodies of HEK293 cells, which, according to a recent report [48], store high numbers of mRNAs.

Based on our results, we propose that cooperation of such protein cofactors (mainly RBPs) with PUM1 or PUM2 might enable regulation of selected groups of RNA targets responsible for a given metabolic pathway in TCam-2 cells. Unfortunately, neither RIP-Seq nor CLIP-Seq data for PUM protein cofactors identified in this study are available for TCam-2 cells. Therefore, we compared our results to eCLIP data from a different cell line, K562 cells, available in ENCODE. We found that the overlap of RNA targets corresponding to a specific PUM–protein cofactor pair in TCam-2 cells with RNA targets of that cofactor in K562 cells was up to 40%. Overlap between TCam-2 PUM1 or PUM2 RIP-Seq targets and eCLIP protein cofactor targets was up to 25% (Table S12). This corroborates our results.

Notably, we found that a number of mRNAs that are enriched in TCam-2 cells compared to somatic gonadal tissue or cause infertility when mutated are under the control of PUM1 or PUM2 RNA regulons, which is in line with their divergent functions. Additionally, each of them consists of subregulons (Figure 4). We propose that identification of germ cell-associated groups of targets that are PUM1- or PUM2-specific might indicate nonredundant roles of PUM paralogues in controlling processes of human reproduction. Notably, the majority of the PUM-regulated genes enriched in TCam-2 cells are genes involved in the development of several types of cancer, mostly of the reproductive system (Table S11). This observation is in concordance with the fact that TCam-2 cells originate from seminoma testis germ cell tumor [24]. It is important to underline, however, that the level of several PUM1- or PUM2-regulated targets was more significantly changed upon PUM1/PUM2 double, than single PUM knockdown. This may reflect a mutual regulation of PUM proteins, as it was previously reported that PUM1 represses PUM2 mRNA and vice versa, since they both contain PBEs in 3′UTR [53]. We have observed this phenomenon of mutual PUM1 and PUM2 repression in TCam-2 cells [54]. Hence, we propose that knockdown of one PUM paralogue activates a feedback, resulting in upregulation of the other PUM. This feedback likely compensates for the lack of repressive activity of the silenced PUM paralogue. Such a feedback mechanism has recently been proposed in a study on PUM regulons that are formed during mouse development, and which was published during the review process of this manuscript [55]. Such a feedback mechanism that enriches the versatility of PUM regulatory mechanisms is an interesting issue to be explored in the future.

Interestingly, PUM RNA regulons overlap at points where PUM1 and PUM2 regulate common targets and interact with common protein cofactors (Tables S4 and S10, respectively). On the other hand, a PUM cofactor may regulate a specific pathway dependent on binding PUM1 or PUM2. For example, FXR2 may regulate endosome transport by binding PUM1 or Rho protein signal transduction by binding PUM2. Likewise, SFPQ may regulate cytosolic or endosome transport by binding PUM1 or endothelium development by binding PUM2.

The majority of the selected PUM targets enriched in TCam-2 cells, compared to gonadal tissue, have been reported to be involved in the regulation of the cell cycle, proliferation, and apoptosis (Table S11), processes that are important for the maintenance of germ-line status and are under precise regulation to ensure fertility. This functional profile is also in line with the recent suggestion that the evolutionarily original role of PUM proteins is regulation of stem cell self-renewal, including germline stem cells renewal [40], which the above-mentioned three processes strongly influence. It is important to keep in mind that the TCam-2 cell line represents a model of germ cells, and does not fully reflect the physiology of human male germ cells.

## 5. Conclusions

As a conclusion, it is worth underlining that despite the same recognition motif, PUM1 and PUM2 proteins regulate partly nonoverlapping groups of mRNAs. Therefore, we propose that PUM1 and PUM2 form RNA regulons with different functions in germ cells. Further studies of post-transcriptional mechanisms of gene expression regulation controlled by PUM proteins in the context of human germ cells are particularly important in light of the increasing problem of male and female infertility occurring worldwide in contemporary populations, as well as the increasing incidence of testis germ cell tumors in young men. In addition, it would be important to study the impact of PUM proteins on stem cell fate, growth, and development, in the context of cancer and neurological disorders. This may provide insight into their diverse roles and enable future therapeutic strategies to target diseases arising from PUM and PUM-target dysfunctions.

## Data Availability

Processed and raw data for RIP-Seq and RNA-Seq experiments described here are available from the Gene Expression Omnibus (accession GSE123016). The mass spectrometry proteomics data have been deposited to the ProteomeXchange Consortium via the PRIDE [56] partner repository with the dataset identifier PXD011948 and 10.6019/PXD011948.

## Supplementary Materials

The following are available online at https://www.mdpi.com/2073-4409/9/4/984/s1, Supplementary Figures are added in separate Word File entitled: “Cells_PUM_Regulon_Supplementary_Figures” and listed: Figure S1: Enrichment of binding motifs for PUM1- or PUM2-interacting RBPs identified in each mRNA regulated by PUM1 or PUM2 in TCam-2 cells; Figure S2: Western blot (WB) analyses showing specificity of PUM1 and PUM2 antibodies, as well as PUM binding efficiency to beads; Figure S3: Heat map of Pearson correlation for comparison of the TCam-2 transcriptome obtained in this study with the TCam-2 transcriptome published by Irie et al., 2015 30; Figure S4: PCA plots showing similarity between the studied biological replicates; Figure S5: Gene Ontology analysis of mRNAs bound to PUM1 and PUM2 as identified by the RIP-Seq approach; Figure S6: Gene Ontology analysis of mRNAs up- and downregulated upon PUM1 and/or PUM2 siRNA knockdown (PUM1 or PUM2 siRNA- Seq); Figure S7: Gene Ontology analysis of PUM1-and PUM2- regulated targets. Supplementary Tables are added in separate ZIP file with Excel files in it and entitled: “Cells_PUM_Regulon_Supplementary_Tables” and listed: Table S1: List of siRNAs used for PUM1 and PUM2 knockdown; Table S2: List of primers for RT-qPCR; Table S3: Mass spectrometry protein identification data; Table S4: PUM1,PUM2 and common protein interactors; Table S5: More than average cofactor motif enrichment in PUM1 and PUM2 regulated mRNAs; Table S6: Gene ontology results related to Figure 4 Regulon model; Table S7: PUM1 and PUM2 RIP-Seq identified mRNA targets; Table S8: GO of RIPSeq siRNASeq Reg mRNAs protein interactors; Table S9: mRNAs activated or repressed upon PUM1 or PUM2 siRNA KD; Table S10: mRNAs regulated by PUM1, PUM2 or PUM1 and PUM2; Table S11: PUM regulated mRNAs OE in TCam-2 cells and infertility Table S12: RIP-Seq PUM targets versus ENCODE CLIP targets of PUM interacting proteins; Supplementary Certificate of Mass Spectrometry Analysis; FASTQC data in zip; Supplementary full western blot for Figure 1E; Detailed Description of Supplementary Tables; References in Supplementary Table S11.
