# Supplementary material for: Characterization of RNP Networks of PUM1 and PUM2 Post-Transcriptional Regulators in TCam-2 Cells, a Human Male Germ Cell Model
Source: Cells. 2020 Apr 16;9(4):984. doi: 10.3390/cells9040984 (PMC7226987; doi:10.3390/cells9040984)
Supplement: Supplementary file 1 [file cells-09-00984-s001.zip › FASTQC data.zip!/QC_after_trimming/RIP/FastQC_IGG-10_1_html.html]

Trimmomatic on Co-IP\_IgG-10\_1\_fastq\_gz \_R1 paired\_ FastQC Report 

FastQC Report

Thu 30 Jan 2020  
Trimmomatic on Co-IP\_IgG-10\_1\_fastq\_gz \_R1 paired\_

## Summary

- Basic Statistics
- Per base sequence quality
- Per tile sequence quality
- Per sequence quality scores
- Per base sequence content
- Per sequence GC content
- Per base N content
- Sequence Length Distribution
- Sequence Duplication Levels
- Overrepresented sequences
- Adapter Content

## Basic Statistics

| Measure | Value |
| --- | --- |
| Filename | Trimmomatic on Co-IP\_IgG-10\_1\_fastq\_gz \_R1 paired\_ |
| File type | Conventional base calls |
| Encoding | Sanger / Illumina 1.9 |
| Total Sequences | 35115545 |
| Sequences flagged as poor quality | 0 |
| Sequence length | 50-101 |
| %GC | 55 |

## Per base sequence quality

## Per tile sequence quality

## Per sequence quality scores

## Per base sequence content

## Per sequence GC content

## Per base N content

## Sequence Length Distribution

## Sequence Duplication Levels

## Overrepresented sequences

| Sequence | Count | Percentage | Possible Source |
| --- | --- | --- | --- |
| CCTCACCCGGCCCGGACACGGACAGGATTGACAGATTGATAGCTCTTTCT | 93386 | 0.26593920157013085 | No Hit |
| CCGCAGGCTCCACTCCTGGTGGTGCCCTTCCGTCAATTCCTTTAAGTTTC | 76342 | 0.217402292916143 | No Hit |
| CTGCCAGTAGCATATGCTTGTCTCAAAGATTAAGCCATGCATGTCTAAGT | 53521 | 0.1524139807598031 | No Hit |
| CTTGTTACGACTTTTACTTCCTCTAGATAGTCAAGTTCGACCGTCTTCTC | 52036 | 0.148185084412046 | No Hit |
| CTCACCCGGCCCGGACACGGACAGGATTGACAGATTGATAGCTCTTTCTC | 48894 | 0.13923748015302057 | No Hit |
| CTCAGACAGGCGTAGCCCCGGGAGGAACCCGGGGCCGCAAGTGCGTTCGA | 47200 | 0.1344134058007643 | No Hit |
| CGGCGCAAGACGGACCAGAGCGAAAGCATTTGCCAAGAATGTTTTCATTA | 45705 | 0.130156032036524 | No Hit |
| CTTAGATGTCCGGGGCTGCACGCGCGCTACACTGACTGGCTCAGCGTGTG | 40346 | 0.11489498454317028 | No Hit |
| CCGGAAGCTGCCCGGCGGGTCATGGGAATAACGCCGCCGCATCGCCGGTC | 40069 | 0.11410616010658528 | No Hit |
| CGGACACGGACAGGATTGACAGATTGATAGCTCTTTCTCGATTCCGTGGG | 40053 | 0.11406059624021214 | No Hit |
| CGGGGCTGCACGCGCGCTACACTGACTGGCTCAGCGTGTGCCTACCCTAC | 40025 | 0.11398085947405914 | No Hit |
| CTCGGCTCGTGCGTCGATGAAGAACGCAGCTAGCTGCGAGAATTAATGTG | 38295 | 0.10905426642246333 | No Hit |
| CTCGGGCCTGCTTTGAACACTCTAATTTTTTCAAAGTAAACGCTTCGGGC | 35986 | 0.10247883095648949 | No Hit |

## Adapter Content

Produced by FastQC (version 0.11.8)
