# Supplementary material for: Characterization of RNP Networks of PUM1 and PUM2 Post-Transcriptional Regulators in TCam-2 Cells, a Human Male Germ Cell Model
Source: Cells. 2020 Apr 16;9(4):984. doi: 10.3390/cells9040984 (PMC7226987; doi:10.3390/cells9040984)
Supplement: Supplementary file 1 [file cells-09-00984-s001.zip › FASTQC data.zip!/QC_after_trimming/RIP/FastQC_IGG-11_1_html.html]

Trimmomatic on Co-IP\_IgG-11\_1\_fastq\_gz \_R1 paired\_ FastQC Report 

FastQC Report

Thu 30 Jan 2020  
Trimmomatic on Co-IP\_IgG-11\_1\_fastq\_gz \_R1 paired\_

## Summary

- Basic Statistics
- Per base sequence quality
- Per tile sequence quality
- Per sequence quality scores
- Per base sequence content
- Per sequence GC content
- Per base N content
- Sequence Length Distribution
- Sequence Duplication Levels
- Overrepresented sequences
- Adapter Content

## Basic Statistics

| Measure | Value |
| --- | --- |
| Filename | Trimmomatic on Co-IP\_IgG-11\_1\_fastq\_gz \_R1 paired\_ |
| File type | Conventional base calls |
| Encoding | Sanger / Illumina 1.9 |
| Total Sequences | 26276731 |
| Sequences flagged as poor quality | 0 |
| Sequence length | 50-101 |
| %GC | 54 |

## Per base sequence quality

## Per tile sequence quality

## Per sequence quality scores

## Per base sequence content

## Per sequence GC content

## Per base N content

## Sequence Length Distribution

## Sequence Duplication Levels

## Overrepresented sequences

| Sequence | Count | Percentage | Possible Source |
| --- | --- | --- | --- |
| CCTCACCCGGCCCGGACACGGACAGGATTGACAGATTGATAGCTCTTTCT | 81263 | 0.309258408133036 | No Hit |
| CCGCAGGCTCCACTCCTGGTGGTGCCCTTCCGTCAATTCCTTTAAGTTTC | 58939 | 0.2243011126460137 | No Hit |
| CTGCCAGTAGCATATGCTTGTCTCAAAGATTAAGCCATGCATGTCTAAGT | 45768 | 0.17417691721241885 | No Hit |
| CTTGTTACGACTTTTACTTCCTCTAGATAGTCAAGTTCGACCGTCTTCTC | 44163 | 0.16806885148689157 | No Hit |
| CTCACCCGGCCCGGACACGGACAGGATTGACAGATTGATAGCTCTTTCTC | 42083 | 0.1601531027584824 | No Hit |
| CTCAGACAGGCGTAGCCCCGGGAGGAACCCGGGGCCGCAAGTGCGTTCGA | 38561 | 0.14674960899816647 | No Hit |
| CTTAGATGTCCGGGGCTGCACGCGCGCTACACTGACTGGCTCAGCGTGTG | 38399 | 0.14613309395297308 | No Hit |
| CTCGGCTCGTGCGTCGATGAAGAACGCAGCTAGCTGCGAGAATTAATGTG | 33407 | 0.12713529700479104 | No Hit |
| CCGGAAGCTGCCCGGCGGGTCATGGGAATAACGCCGCCGCATCGCCGGTC | 32850 | 0.1250155508308853 | No Hit |
| CTTGTCTCAAAGATTAAGCCATGCATGTCTAAGTACGCACGGCCGGTACA | 32061 | 0.1220128942218878 | No Hit |
| CGGGGCTGCACGCGCGCTACACTGACTGGCTCAGCGTGTGCCTACCCTAC | 31790 | 0.12098156349813834 | No Hit |
| CGGCGCAAGACGGACCAGAGCGAAAGCATTTGCCAAGAATGTTTTCATTA | 31457 | 0.11971428257190744 | No Hit |
| GTCGGCATGTATTAGCTCTAGAATTACCACAGTTATCCAAGTAGGAGAGG | 31118 | 0.11842416775511384 | No Hit |
| CTCGGGCCTGCTTTGAACACTCTAATTTTTTCAAAGTAAACGCTTCGGGC | 30720 | 0.11690951968112015 | No Hit |
| CTAACACGTGCGCGAGTCGGGGGCTCGCACGAAAGCCGCCGTGGCGCAAT | 28234 | 0.10744867769130034 | No Hit |
| CGCGTAACTAGTTAGCATGCCAGAGTCTCGTTCGTTATCGGAATTAACCA | 28031 | 0.10667613106059501 | No Hit |
| CCAGAGTCTCGTTCGTTATCGGAATTAACCAGACAAATCGCTCCACCAAC | 27569 | 0.10491792148726567 | No Hit |
| CGGACACGGACAGGATTGACAGATTGATAGCTCTTTCTCGATTCCGTGGG | 27522 | 0.10473905601119105 | No Hit |
| CGCAGGCTCCACTCCTGGTGGTGCCCTTCCGTCAATTCCTTTAAGTTTCA | 26855 | 0.10220068851030215 | No Hit |
| CGCTGAGCCAGTCAGTGTAGCGCGCGTGCAGCCCCGGACATCTAAGGGCA | 26536 | 0.10098668666205092 | No Hit |
| ACGACTTTTACTTCCTCTAGATAGTCAAGTTCGACCGTCTTCTCAGCGCT | 26525 | 0.10094482452935259 | No Hit |

## Adapter Content

Produced by FastQC (version 0.11.8)
