# Supplementary material for: Characterization of RNP Networks of PUM1 and PUM2 Post-Transcriptional Regulators in TCam-2 Cells, a Human Male Germ Cell Model
Source: Cells. 2020 Apr 16;9(4):984. doi: 10.3390/cells9040984 (PMC7226987; doi:10.3390/cells9040984)
Supplement: Supplementary file 1 [file cells-09-00984-s001.zip › FASTQC data.zip!/QC_after_trimming/RIP/FastQC_IGG-11_2_html.html]

Trimmomatic on Co-IP\_IgG-11\_2\_fastq\_gz \_R2 paired\_ FastQC Report 

FastQC Report

Thu 30 Jan 2020  
Trimmomatic on Co-IP\_IgG-11\_2\_fastq\_gz \_R2 paired\_

## Summary

- Basic Statistics
- Per base sequence quality
- Per tile sequence quality
- Per sequence quality scores
- Per base sequence content
- Per sequence GC content
- Per base N content
- Sequence Length Distribution
- Sequence Duplication Levels
- Overrepresented sequences
- Adapter Content

## Basic Statistics

| Measure | Value |
| --- | --- |
| Filename | Trimmomatic on Co-IP\_IgG-11\_2\_fastq\_gz \_R2 paired\_ |
| File type | Conventional base calls |
| Encoding | Sanger / Illumina 1.9 |
| Total Sequences | 26276731 |
| Sequences flagged as poor quality | 0 |
| Sequence length | 50-101 |
| %GC | 54 |

## Per base sequence quality

## Per tile sequence quality

## Per sequence quality scores

## Per base sequence content

## Per sequence GC content

## Per base N content

## Sequence Length Distribution

## Sequence Duplication Levels

## Overrepresented sequences

| Sequence | Count | Percentage | Possible Source |
| --- | --- | --- | --- |
| CCTCACCCGGCCCGGACACGGACAGGATTGACAGATTGATAGCTCTTTCT | 86282 | 0.3283589575887503 | No Hit |
| CCGCAGGCTCCACTCCTGGTGGTGCCCTTCCGTCAATTCCTTTAAGTTTC | 59801 | 0.22758158159019096 | No Hit |
| CTCACCCGGCCCGGACACGGACAGGATTGACAGATTGATAGCTCTTTCTC | 43725 | 0.1664019774758131 | No Hit |
| CTGCCAGTAGCATATGCTTGTCTCAAAGATTAAGCCATGCATGTCTAAGT | 41038 | 0.1561762001521422 | No Hit |
| CTTGTTACGACTTTTACTTCCTCTAGATAGTCAAGTTCGACCGTCTTCTC | 39804 | 0.1514800299930764 | No Hit |
| CTCAGACAGGCGTAGCCCCGGGAGGAACCCGGGGCCGCAAGTGCGTTCGA | 37896 | 0.14421885279413182 | No Hit |
| CTCGGCTCGTGCGTCGATGAAGAACGCAGCTAGCTGCGAGAATTAATGTG | 37499 | 0.14270801036856526 | No Hit |
| CGGCGCAAGACGGACCAGAGCGAAAGCATTTGCCAAGAATGTTTTCATTA | 33047 | 0.12576526357102794 | No Hit |
| CTCGGGCCTGCTTTGAACACTCTAATTTTTTCAAAGTAAACGCTTCGGGC | 33003 | 0.12559781504023465 | No Hit |
| GTCGGCATGTATTAGCTCTAGAATTACCACAGTTATCCAAGTAGGAGAGG | 32503 | 0.12369499082667473 | No Hit |
| CTTAGATGTCCGGGGCTGCACGCGCGCTACACTGACTGGCTCAGCGTGTG | 32440 | 0.12345523497576619 | No Hit |
| CTTGTCTCAAAGATTAAGCCATGCATGTCTAAGTACGCACGGCCGGTACA | 29677 | 0.11294022837163421 | No Hit |
| CGGGGCTGCACGCGCGCTACACTGACTGGCTCAGCGTGTGCCTACCCTAC | 29622 | 0.11273091770814261 | No Hit |
| CGGACACGGACAGGATTGACAGATTGATAGCTCTTTCTCGATTCCGTGGG | 28810 | 0.10964073118532133 | No Hit |
| CGCTGAGCCAGTCAGTGTAGCGCGCGTGCAGCCCCGGACATCTAAGGGCA | 28668 | 0.10910032910867032 | No Hit |
| CGCGTAACTAGTTAGCATGCCAGAGTCTCGTTCGTTATCGGAATTAACCA | 28351 | 0.10789393855727336 | No Hit |
| CGCAGGCTCCACTCCTGGTGGTGCCCTTCCGTCAATTCCTTTAAGTTTCA | 27885 | 0.10612050639023554 | No Hit |
| CCGGAAGCTGCCCGGCGGGTCATGGGAATAACGCCGCCGCATCGCCGGTC | 27308 | 0.10392464724778741 | No Hit |
| CTAACACGTGCGCGAGTCGGGGGCTCGCACGAAAGCCGCCGTGGCGCAAT | 26344 | 0.10025600216404393 | No Hit |
| CCCGCACTTACTGGGAATTCCTCGTTCATGGGGAATAATTGCAATCCCCG | 26289 | 0.10004669150055234 | No Hit |

## Adapter Content

Produced by FastQC (version 0.11.8)
