# Supplementary material for: Characterization of RNP Networks of PUM1 and PUM2 Post-Transcriptional Regulators in TCam-2 Cells, a Human Male Germ Cell Model
Source: Cells. 2020 Apr 16;9(4):984. doi: 10.3390/cells9040984 (PMC7226987; doi:10.3390/cells9040984)
Supplement: Supplementary file 1 [file cells-09-00984-s001.zip › FASTQC data.zip!/QC_after_trimming/RIP/FastQC_IGG-9_2_html.html]

Trimmomatic on Co-IP\_IgG-9\_2\_fastq\_gz \_R2 paired\_ FastQC Report 

FastQC Report

Thu 30 Jan 2020  
Trimmomatic on Co-IP\_IgG-9\_2\_fastq\_gz \_R2 paired\_

## Summary

- Basic Statistics
- Per base sequence quality
- Per tile sequence quality
- Per sequence quality scores
- Per base sequence content
- Per sequence GC content
- Per base N content
- Sequence Length Distribution
- Sequence Duplication Levels
- Overrepresented sequences
- Adapter Content

## Basic Statistics

| Measure | Value |
| --- | --- |
| Filename | Trimmomatic on Co-IP\_IgG-9\_2\_fastq\_gz \_R2 paired\_ |
| File type | Conventional base calls |
| Encoding | Sanger / Illumina 1.9 |
| Total Sequences | 32849991 |
| Sequences flagged as poor quality | 0 |
| Sequence length | 50-101 |
| %GC | 52 |

## Per base sequence quality

## Per tile sequence quality

## Per sequence quality scores

## Per base sequence content

## Per sequence GC content

## Per base N content

## Sequence Length Distribution

## Sequence Duplication Levels

## Overrepresented sequences

| Sequence | Count | Percentage | Possible Source |
| --- | --- | --- | --- |
| CCTCACCCGGCCCGGACACGGACAGGATTGACAGATTGATAGCTCTTTCT | 73386 | 0.2233973214787182 | No Hit |
| CCGCAGGCTCCACTCCTGGTGGTGCCCTTCCGTCAATTCCTTTAAGTTTC | 53124 | 0.1617169392831797 | No Hit |
| CTTAGATGTCCGGGGCTGCACGCGCGCTACACTGACTGGCTCAGCGTGTG | 43689 | 0.13299547022706948 | No Hit |
| CTCACCCGGCCCGGACACGGACAGGATTGACAGATTGATAGCTCTTTCTC | 34047 | 0.10364386401201754 | No Hit |

## Adapter Content

Produced by FastQC (version 0.11.8)
