# Supplementary material for: Characterization of RNP Networks of PUM1 and PUM2 Post-Transcriptional Regulators in TCam-2 Cells, a Human Male Germ Cell Model
Source: Cells. 2020 Apr 16;9(4):984. doi: 10.3390/cells9040984 (PMC7226987; doi:10.3390/cells9040984)
Supplement: Supplementary file 1 [file cells-09-00984-s001.zip › FASTQC data.zip!/QC_after_trimming/RIP/FastQC_PUM1-7_1_html.html]

Trimmomatic on Co-IP\_PUM1-7\_1\_fastq\_gz \_R1 paired\_ FastQC Report 

FastQC Report

Thu 30 Jan 2020  
Trimmomatic on Co-IP\_PUM1-7\_1\_fastq\_gz \_R1 paired\_

## Summary

- Basic Statistics
- Per base sequence quality
- Per tile sequence quality
- Per sequence quality scores
- Per base sequence content
- Per sequence GC content
- Per base N content
- Sequence Length Distribution
- Sequence Duplication Levels
- Overrepresented sequences
- Adapter Content

## Basic Statistics

| Measure | Value |
| --- | --- |
| Filename | Trimmomatic on Co-IP\_PUM1-7\_1\_fastq\_gz \_R1 paired\_ |
| File type | Conventional base calls |
| Encoding | Sanger / Illumina 1.9 |
| Total Sequences | 35216464 |
| Sequences flagged as poor quality | 0 |
| Sequence length | 50-101 |
| %GC | 53 |

## Per base sequence quality

## Per tile sequence quality

## Per sequence quality scores

## Per base sequence content

## Per sequence GC content

## Per base N content

## Sequence Length Distribution

## Sequence Duplication Levels

## Overrepresented sequences

| Sequence | Count | Percentage | Possible Source |
| --- | --- | --- | --- |
| CCTCACCCGGCCCGGACACGGACAGGATTGACAGATTGATAGCTCTTTCT | 180355 | 0.5121326206969559 | No Hit |
| CTCACCCGGCCCGGACACGGACAGGATTGACAGATTGATAGCTCTTTCTC | 118860 | 0.33751259070189443 | No Hit |
| CGCGTAACTAGTTAGCATGCCAGAGTCTCGTTCGTTATCGGAATTAACCA | 95080 | 0.2699873559139839 | No Hit |
| GTCGGCATGTATTAGCTCTAGAATTACCACAGTTATCCAAGTAGGAGAGG | 74580 | 0.21177594661406096 | No Hit |
| CTGCCAGTAGCATATGCTTGTCTCAAAGATTAAGCCATGCATGTCTAAGT | 71308 | 0.202484837773605 | No Hit |
| CTTAGATGTCCGGGGCTGCACGCGCGCTACACTGACTGGCTCAGCGTGTG | 67606 | 0.19197270912832134 | No Hit |
| CCCGTCGGCATGTATTAGCTCTAGAATTACCACAGTTATCCAAGTAGGAG | 64312 | 0.18261912950715325 | No Hit |
| CTGCTGCCTTCCTTGGATGTGGTAGCCGTTTCTCAGGCTCCCTCTCCGGA | 59349 | 0.168526289294689 | No Hit |
| CTCCGACTTTCGTTCTTGATTAATGAAAACATTCTTGGCAAATGCTTTCG | 55316 | 0.15707425935778221 | No Hit |
| CTGCGGTATCCAGGCGGCTCGGGCCTGCTTTGAACACTCTAATTTTTTCA | 54509 | 0.15478271753802425 | No Hit |
| CTTGTCTCAAAGATTAAGCCATGCATGTCTAAGTACGCACGGCCGGTACA | 52562 | 0.1492540534450023 | No Hit |
| CTCTGGTCCGTCTTGCGCCGGTCCAAGAATTTCACCTCTAGCGGCGCAAT | 52048 | 0.14779450884109205 | No Hit |
| CAAAGGGCAGGGACTTAATCAACGCAAGCTTATGACCCGCACTTACTGGG | 51981 | 0.14760425691801426 | No Hit |
| CTCGTTCGTTATCGGAATTAACCAGACAAATCGCTCCACCAACTAAGAAC | 51921 | 0.14743388206152666 | No Hit |
| CTCCAATGGATCCTCGTTAAAGGATTTAAAGTGGACTCATTCCAATTACA | 51842 | 0.14720955516715137 | No Hit |
| CTTGTTACGACTTTTACTTCCTCTAGATAGTCAAGTTCGACCGTCTTCTC | 51343 | 0.14579260427736299 | No Hit |
| CTCGGGCCTGCTTTGAACACTCTAATTTTTTCAAAGTAAACGCTTCGGGC | 49413 | 0.1403122130603459 | No Hit |
| CAAAGATTAAGCCATGCATGTCTAAGTACGCACGGCCGGTACAGTGAAAC | 46310 | 0.13150099339899657 | No Hit |
| CCCGCACTTACTGGGAATTCCTCGTTCATGGGGAATAATTGCAATCCCCG | 45734 | 0.1298653947767158 | No Hit |
| CTAACACGTGCGCGAGTCGGGGGCTCGCACGAAAGCCGCCGTGGCGCAAT | 45496 | 0.1291895745126484 | No Hit |
| CTTCCGTCAATTCCTTTAAGTTTCAGCTTTGCAACCATACTCCCCCCGGA | 44975 | 0.12771015284214793 | No Hit |
| CCGGAAGCTGCCCGGCGGGTCATGGGAATAACGCCGCCGCATCGCCGGTC | 43555 | 0.12367794790527523 | No Hit |
| CTCGTTCATGGGGAATAATTGCAATCCCCGATCCCCATCACGAATGGGGT | 42410 | 0.1204266277273039 | No Hit |
| CAGGGACTTAATCAACGCAAGCTTATGACCCGCACTTACTGGGAATTCCT | 41985 | 0.11921980582718356 | No Hit |
| CTTGGACCGGCGCAAGACGGACCAGAGCGAAAGCATTTGCCAAGAATGTT | 41565 | 0.1180271818317705 | No Hit |
| CCGGTATTTAGCCTTAGATGGAGTTTACCACCCGCTTTGGGCTGCATTCC | 41088 | 0.11667270172269426 | No Hit |
| GTTGGATCTTGGGAGCGGGCGGGCGGTCCGCCGCGAGGCGAGCCACCGCC | 39695 | 0.11271716547124094 | No Hit |
| CTCAGTTCCGAAAACCAACAAAATAGAACCGCGGTCCTATTCCATTATTC | 39551 | 0.11230826581567076 | No Hit |
| CCGACTTTCGTTCTTGATTAATGAAAACATTCTTGGCAAATGCTTTCGCT | 38907 | 0.11047957568937074 | No Hit |
| CTGCTTTGAACACTCTAATTTTTTCAAAGTAAACGCTTCGGGCCCCGCGG | 37869 | 0.10753209067213562 | No Hit |
| CCGTCGGCATGTATTAGCTCTAGAATTACCACAGTTATCCAAGTAGGAGA | 37411 | 0.10623156260094711 | No Hit |
| GTTAGTTTTACCCTACTGATGATGTGTTGTTGCCATGGTAATCCTGCTCA | 35684 | 0.10132760631504628 | No Hit |
| GCCGTTCTTAGTTGGTGGAGCGATTTGTCTGGTTAATTCCGATAACGAAC | 35224 | 0.10002139908197483 | No Hit |

## Adapter Content

Produced by FastQC (version 0.11.8)
