# Supplementary material for: Characterization of RNP Networks of PUM1 and PUM2 Post-Transcriptional Regulators in TCam-2 Cells, a Human Male Germ Cell Model
Source: Cells. 2020 Apr 16;9(4):984. doi: 10.3390/cells9040984 (PMC7226987; doi:10.3390/cells9040984)
Supplement: Supplementary file 1 [file cells-09-00984-s001.zip › FASTQC data.zip!/QC_after_trimming/RIP/FastQC_PUM2-3_1_html.html]

Trimmomatic on Co-IP\_PUM2-3\_1\_fastq\_gz \_R1 paired\_ FastQC Report 

FastQC Report

Thu 30 Jan 2020  
Trimmomatic on Co-IP\_PUM2-3\_1\_fastq\_gz \_R1 paired\_

## Summary

- Basic Statistics
- Per base sequence quality
- Per tile sequence quality
- Per sequence quality scores
- Per base sequence content
- Per sequence GC content
- Per base N content
- Sequence Length Distribution
- Sequence Duplication Levels
- Overrepresented sequences
- Adapter Content

## Basic Statistics

| Measure | Value |
| --- | --- |
| Filename | Trimmomatic on Co-IP\_PUM2-3\_1\_fastq\_gz \_R1 paired\_ |
| File type | Conventional base calls |
| Encoding | Sanger / Illumina 1.9 |
| Total Sequences | 28912180 |
| Sequences flagged as poor quality | 0 |
| Sequence length | 50-101 |
| %GC | 53 |

## Per base sequence quality

## Per tile sequence quality

## Per sequence quality scores

## Per base sequence content

## Per sequence GC content

## Per base N content

## Sequence Length Distribution

## Sequence Duplication Levels

## Overrepresented sequences

| Sequence | Count | Percentage | Possible Source |
| --- | --- | --- | --- |
| CCTCACCCGGCCCGGACACGGACAGGATTGACAGATTGATAGCTCTTTCT | 124517 | 0.4306731626601661 | No Hit |
| CTCACCCGGCCCGGACACGGACAGGATTGACAGATTGATAGCTCTTTCTC | 83219 | 0.287833708838282 | No Hit |
| CCGGTATTTAGCCTTAGATGGAGTTTACCACCCGCTTTGGGCTGCATTCC | 75785 | 0.2621213620003749 | No Hit |
| CCCGTCGGCATGTATTAGCTCTAGAATTACCACAGTTATCCAAGTAGGAG | 68887 | 0.2382629051147302 | No Hit |
| CGCGTAACTAGTTAGCATGCCAGAGTCTCGTTCGTTATCGGAATTAACCA | 65363 | 0.22607427042858752 | No Hit |
| CTGGATAGTAGGTAGGGACAGTGGGAATCTCGTTCATCCATTCATGCGCG | 59183 | 0.2046991959789957 | No Hit |
| CTGCTGCCTTCCTTGGATGTGGTAGCCGTTTCTCAGGCTCCCTCTCCGGA | 58436 | 0.20211550979552562 | No Hit |
| CTGCCAGTAGCATATGCTTGTCTCAAAGATTAAGCCATGCATGTCTAAGT | 57705 | 0.19958716361063053 | No Hit |
| CTCCGACTTTCGTTCTTGATTAATGAAAACATTCTTGGCAAATGCTTTCG | 55852 | 0.1931781000256639 | No Hit |
| GTCGGCATGTATTAGCTCTAGAATTACCACAGTTATCCAAGTAGGAGAGG | 53608 | 0.1854166652255209 | No Hit |
| CTGCCGTATCGTTCCGCCTGGGCGGGATTCTGACTTAGAGGCGTTCAGTC | 51688 | 0.17877586539652146 | No Hit |
| CTTAGATGTCCGGGGCTGCACGCGCGCTACACTGACTGGCTCAGCGTGTG | 50402 | 0.17432791301105624 | No Hit |
| CTAACACGTGCGCGAGTCGGGGGCTCGCACGAAAGCCGCCGTGGCGCAAT | 45963 | 0.15897452215640606 | No Hit |
| GTCGGGTCTGCGAGAGCGCCAGCTATCCTGAGGGAAACTTCGGAGGGAAC | 45496 | 0.1573592859479984 | No Hit |
| CTCCCTTTCGATCGGCCGAGGGCAACGGAGGCCATCGCCCGTCCCTTCGG | 45388 | 0.15698574095761716 | No Hit |
| CTGGGGTCTGATGAGCGTCGGCATCGGGCGCCTTAACCCGGCGTTCGGTT | 42461 | 0.146861979968304 | No Hit |
| CAGAAACCTCCCGTGGAGCAGAAGGGCAAAAGCTCGCTTGATCTTGATTT | 40852 | 0.14129685136160608 | No Hit |
| CTGCTTTGAACACTCTAATTTTTTCAAAGTAAACGCTTCGGGCCCCGCGG | 39675 | 0.137225902716433 | No Hit |
| CTCAGTTCCGAAAACCAACAAAATAGAACCGCGGTCCTATTCCATTATTC | 38609 | 0.13353887531137396 | No Hit |
| CTGCTGTCTATATCAACCAACACCTTTTCTGGGGTCTGATGAGCGTCGGC | 38464 | 0.13303735657428806 | No Hit |
| CTTCCGTCAATTCCTTTAAGTTTCAGCTTTGCAACCATACTCCCCCCGGA | 37070 | 0.1282158591984416 | No Hit |
| CTTGAACTCTCTCTTCAAAGTTCTTTTCAACTTTCCCTTACGGTACTTGT | 36861 | 0.12749298046705576 | No Hit |
| CCCGCACTTACTGGGAATTCCTCGTTCATGGGGAATAATTGCAATCCCCG | 36456 | 0.12609218675312617 | No Hit |
| CTTGTCTCAAAGATTAAGCCATGCATGTCTAAGTACGCACGGCCGGTACA | 36269 | 0.12544540051978093 | No Hit |
| CTTCCGTACGCCACATGTCCCGCGCCCCGCCGCGGGGCGGGGATTCGGCG | 36018 | 0.1245772542921357 | No Hit |
| CCCAGGCATAGTTCACCATCTTTCGGGTCCTAACACGTGCGCTCGTGCTC | 35358 | 0.12229447935091714 | No Hit |
| CAGGGACTTAATCAACGCAAGCTTATGACCCGCACTTACTGGGAATTCCT | 35096 | 0.12138828687425161 | No Hit |
| CAAAGGGCAGGGACTTAATCAACGCAAGCTTATGACCCGCACTTACTGGG | 34909 | 0.12074150064090634 | No Hit |
| CAAAGATTAAGCCATGCATGTCTAAGTACGCACGGCCGGTACAGTGAAAC | 34432 | 0.11909167693338932 | No Hit |
| GTTAGTTTTACCCTACTGATGATGTGTTGTTGCCATGGTAATCCTGCTCA | 34221 | 0.11836188070218157 | No Hit |
| CGCCTGCTGCCTTCCTTGGATGTGGTAGCCGTTTCTCAGGCTCCCTCTCC | 33707 | 0.11658408324795985 | No Hit |
| CTGCGGTATCCAGGCGGCTCGGGCCTGCTTTGAACACTCTAATTTTTTCA | 33163 | 0.11470252329641004 | No Hit |
| CTCCGCCACTCCGGATTCGGGGATCTGAACCCGACTCCCTTTCGATCGGC | 33121 | 0.11455725580015066 | No Hit |
| CTCGTTCGTTATCGGAATTAACCAGACAAATCGCTCCACCAACTAAGAAC | 32488 | 0.11236786710652742 | No Hit |
| CTCCACTTCGGCCTTCAAAGTTCTCGTTTGAATATTTGCTACTACCACCA | 32137 | 0.11115384588778847 | No Hit |
| CTCCCGTCCACTCTCGACTGCCGGCGACGGCCGGGTATGGGCCCGACGCT | 31994 | 0.11065924465052444 | No Hit |
| CTCGGGCCTGCTTTGAACACTCTAATTTTTTCAAAGTAAACGCTTCGGGC | 30992 | 0.10719357723976539 | No Hit |
| GTCAAAGTGAAGAAATTCAATGAAGCGCGGGTAAACGGCGGGAGTAACTA | 30826 | 0.10661942475454982 | No Hit |
| CTGCCTTCCTTGGATGTGGTAGCCGTTTCTCAGGCTCCCTCTCCGGAATC | 30670 | 0.10607985976844361 | No Hit |
| CTTAATCATGGCCTCAGTTCCGAAAACCAACAAAATAGAACCGCGGTCCT | 30627 | 0.1059311335222733 | No Hit |
| CTTTGAACACTCTAATTTTTTCAAAGTAAACGCTTCGGGCCCCGCGGGAC | 30276 | 0.10471711230353437 | No Hit |
| CCGACATCGAAGGATCAAAAAGCGACGTCGCTATGAACGCTTGGCCGCCA | 29921 | 0.10348925608515167 | No Hit |
| CTTCACCGTGCCAGACTAGAGTCAAGCTCAACAGGGTCTTCTTTCCCCGC | 29857 | 0.10326789609085169 | No Hit |
| CTCTGGTCCGTCTTGCGCCGGTCCAAGAATTTCACCTCTAGCGGCGCAAT | 29428 | 0.10178409237905962 | No Hit |
| CCGTCGGCATGTATTAGCTCTAGAATTACCACAGTTATCCAAGTAGGAGA | 29292 | 0.10131370239117217 | No Hit |
| CCCGAAGTTACGGATCCGGCTTGCCGACTTCCCTTACCTACATTGTTCCA | 29194 | 0.10097474489990033 | No Hit |

## Adapter Content

Produced by FastQC (version 0.11.8)
