# Supplementary material for: Characterization of RNP Networks of PUM1 and PUM2 Post-Transcriptional Regulators in TCam-2 Cells, a Human Male Germ Cell Model
Source: Cells. 2020 Apr 16;9(4):984. doi: 10.3390/cells9040984 (PMC7226987; doi:10.3390/cells9040984)
Supplement: Supplementary file 1 [file cells-09-00984-s001.zip › FASTQC data.zip!/QC_after_trimming/RIP/FastQC_PUM2-4_1_html.html]

Trimmomatic on Co-IP\_PUM2-4\_1\_fastq\_gz \_R1 paired\_ FastQC Report 

FastQC Report

Thu 30 Jan 2020  
Trimmomatic on Co-IP\_PUM2-4\_1\_fastq\_gz \_R1 paired\_

## Summary

- Basic Statistics
- Per base sequence quality
- Per tile sequence quality
- Per sequence quality scores
- Per base sequence content
- Per sequence GC content
- Per base N content
- Sequence Length Distribution
- Sequence Duplication Levels
- Overrepresented sequences
- Adapter Content

## Basic Statistics

| Measure | Value |
| --- | --- |
| Filename | Trimmomatic on Co-IP\_PUM2-4\_1\_fastq\_gz \_R1 paired\_ |
| File type | Conventional base calls |
| Encoding | Sanger / Illumina 1.9 |
| Total Sequences | 34374948 |
| Sequences flagged as poor quality | 0 |
| Sequence length | 50-101 |
| %GC | 53 |

## Per base sequence quality

## Per tile sequence quality

## Per sequence quality scores

## Per base sequence content

## Per sequence GC content

## Per base N content

## Sequence Length Distribution

## Sequence Duplication Levels

## Overrepresented sequences

| Sequence | Count | Percentage | Possible Source |
| --- | --- | --- | --- |
| CCTCACCCGGCCCGGACACGGACAGGATTGACAGATTGATAGCTCTTTCT | 119306 | 0.3470725250260742 | No Hit |
| CTGGATAGTAGGTAGGGACAGTGGGAATCTCGTTCATCCATTCATGCGCG | 92182 | 0.26816622384417865 | No Hit |
| CCGGTATTTAGCCTTAGATGGAGTTTACCACCCGCTTTGGGCTGCATTCC | 83818 | 0.2438345506733567 | No Hit |
| CTTGAACTCTCTCTTCAAAGTTCTTTTCAACTTTCCCTTACGGTACTTGT | 80775 | 0.23498217364576085 | No Hit |
| CTCACCCGGCCCGGACACGGACAGGATTGACAGATTGATAGCTCTTTCTC | 79163 | 0.2302927120064298 | No Hit |
| GTCGGGTCTGCGAGAGCGCCAGCTATCCTGAGGGAAACTTCGGAGGGAAC | 76909 | 0.22373561117823362 | No Hit |
| CGCGTAACTAGTTAGCATGCCAGAGTCTCGTTCGTTATCGGAATTAACCA | 76864 | 0.22360470188929452 | No Hit |
| CTTAGATGTCCGGGGCTGCACGCGCGCTACACTGACTGGCTCAGCGTGTG | 76447 | 0.2223916091451251 | No Hit |
| CTGCTGCCTTCCTTGGATGTGGTAGCCGTTTCTCAGGCTCCCTCTCCGGA | 75697 | 0.22020978766280605 | No Hit |
| CCCGTCGGCATGTATTAGCTCTAGAATTACCACAGTTATCCAAGTAGGAG | 75081 | 0.21841778495199468 | No Hit |
| GTCGGCATGTATTAGCTCTAGAATTACCACAGTTATCCAAGTAGGAGAGG | 72726 | 0.2115668654975129 | No Hit |
| CAGAAACCTCCCGTGGAGCAGAAGGGCAAAAGCTCGCTTGATCTTGATTT | 64407 | 0.18736610161563008 | No Hit |
| CAAAGGGCAGGGACTTAATCAACGCAAGCTTATGACCCGCACTTACTGGG | 63267 | 0.18404973296250515 | No Hit |
| CTTGTCTCAAAGATTAAGCCATGCATGTCTAAGTACGCACGGCCGGTACA | 62683 | 0.18235082130160604 | No Hit |
| CTGCCTTCCTTGGATGTGGTAGCCGTTTCTCAGGCTCCCTCTCCGGAATC | 59586 | 0.1733413531272833 | No Hit |
| CAAAGATTAAGCCATGCATGTCTAAGTACGCACGGCCGGTACAGTGAAAC | 59087 | 0.17188971456771365 | No Hit |
| CTGCCGTATCGTTCCGCCTGGGCGGGATTCTGACTTAGAGGCGTTCAGTC | 56957 | 0.1656933415579276 | No Hit |
| CGCCTGCTGCCTTCCTTGGATGTGGTAGCCGTTTCTCAGGCTCCCTCTCC | 51895 | 0.15096750109992893 | No Hit |
| CAGGGACTTAATCAACGCAAGCTTATGACCCGCACTTACTGGGAATTCCT | 49889 | 0.14513185590855293 | No Hit |
| CTCCCTTTCGATCGGCCGAGGGCAACGGAGGCCATCGCCCGTCCCTTCGG | 49840 | 0.14498931023837475 | No Hit |
| CTGGGGTCTGATGAGCGTCGGCATCGGGCGCCTTAACCCGGCGTTCGGTT | 49252 | 0.14327876219623664 | No Hit |
| CTTGGCTGTGGTTTCGCTGGATAGTAGGTAGGGACAGTGGGAATCTCGTT | 49047 | 0.14268239765773608 | No Hit |
| CTTTGAACACTCTAATTTTTTCAAAGTAAACGCTTCGGGCCCCGCGGGAC | 48716 | 0.14171948711020596 | No Hit |
| CTTAATCATGGCCTCAGTTCCGAAAACCAACAAAATAGAACCGCGGTCCT | 46163 | 0.13429256678439194 | No Hit |
| CTCCACTTCGGCCTTCAAAGTTCTCGTTTGAATATTTGCTACTACCACCA | 44940 | 0.130734743220557 | No Hit |
| CTCCGCCACTCCGGATTCGGGGATCTGAACCCGACTCCCTTTCGATCGGC | 44893 | 0.13059801574099836 | No Hit |
| CGAAGCCAGAGGAAACTCTGGTGGAGGTCCGTAGCGGTCCTGACGTGCAA | 42892 | 0.12477691602617116 | No Hit |
| GTTAGTTTTACCCTACTGATGATGTGTTGTTGCCATGGTAATCCTGCTCA | 42806 | 0.1245267338295319 | No Hit |
| CTAACACGTGCGCGAGTCGGGGGCTCGCACGAAAGCCGCCGTGGCGCAAT | 42624 | 0.12399727848315582 | No Hit |
| CCGACTTTCGTTCTTGATTAATGAAAACATTCTTGGCAAATGCTTTCGCT | 42167 | 0.12266782192659607 | No Hit |
| CTCTGGTGGAGGTCCGTAGCGGTCCTGACGTGCAAATCGGTCGTCCGACC | 40520 | 0.11787654195142346 | No Hit |
| CCACTCTCGACTGCCGGCGACGGCCGGGTATGGGCCCGACGCTCCAGCGC | 39993 | 0.11634344872318061 | No Hit |
| CGGGTCTGCGAGAGCGCCAGCTATCCTGAGGGAAACTTCGGAGGGAACCA | 39502 | 0.1149150829260891 | No Hit |
| CGGCCTTCAAAGTTCTCGTTTGAATATTTGCTACTACCACCAAGATCTGC | 39438 | 0.11472890082626452 | No Hit |
| CTTGGACCGGCGCAAGACGGACCAGAGCGAAAGCATTTGCCAAGAATGTT | 39064 | 0.11364089918041477 | No Hit |
| GGAAACTCTGGTGGAGGTCCGTAGCGGTCCTGACGTGCAAATCGGTCGTC | 38846 | 0.11300671640288736 | No Hit |
| CGGGCTTCTTACCCATTTAAAGTTTGAGAATAGGTTGAGATCGTTTCGGC | 38084 | 0.11078998577685122 | No Hit |
| CGCCACTCCGGATTCGGGGATCTGAACCCGACTCCCTTTCGATCGGCCGA | 37860 | 0.11013834842746525 | No Hit |
| CTCGGGCCTGCTTTGAACACTCTAATTTTTTCAAAGTAAACGCTTCGGGC | 37659 | 0.10955362027020375 | No Hit |
| CGCGCCTGCTGCCTTCCTTGGATGTGGTAGCCGTTTCTCAGGCTCCCTCT | 37594 | 0.10936452907506944 | No Hit |
| CTCGTTCGTTATCGGAATTAACCAGACAAATCGCTCCACCAACTAAGAAC | 36737 | 0.10687143439460621 | No Hit |
| GTCAAAGTGAAGAAATTCAATGAAGCGCGGGTAAACGGCGGGAGTAACTA | 36670 | 0.10667652500885239 | No Hit |
| CTCCGACTTTCGTTCTTGATTAATGAAAACATTCTTGGCAAATGCTTTCG | 36408 | 0.10591434203769559 | No Hit |
| GTCTGCGAGAGCGCCAGCTATCCTGAGGGAAACTTCGGAGGGAACCAGCT | 36395 | 0.10587652379866874 | No Hit |
| CCCAGGCATAGTTCACCATCTTTCGGGTCCTAACACGTGCGCTCGTGCTC | 36337 | 0.10570779627070272 | No Hit |
| CTGCTGTCTATATCAACCAACACCTTTTCTGGGGTCTGATGAGCGTCGGC | 36244 | 0.10543725040689515 | No Hit |
| CTTCCGTCAATTCCTTTAAGTTTCAGCTTTGCAACCATACTCCCCCCGGA | 35818 | 0.10419797580493795 | No Hit |
| CGCCCATCTCTCAGGACCGACTGACCCATGTTCAACTGCTGTTCACATGG | 35748 | 0.10399433913325484 | No Hit |
| CTTCCTTGGATGTGGTAGCCGTTTCTCAGGCTCCCTCTCCGGAATCGAAC | 35649 | 0.10370633869758873 | No Hit |
| CTTTTCTGACACCTCCTGCTTAAAACCCAAAAGGTCAGAAGGATCGTGAG | 35452 | 0.10313324692156625 | No Hit |
| CCGACATCGAAGGATCAAAAAGCGACGTCGCTATGAACGCTTGGCCGCCA | 35350 | 0.10283651919997087 | No Hit |
| CCGAAGTTTCCCTCAGGATAGCTGGCGCTCTCGCAGACCCGACGCACCCC | 35034 | 0.10191724508208712 | No Hit |
| CTGAATTTAAGCATATTAGTCAGCGGAGGAAAAGAAACTAACCAGGATTC | 34953 | 0.10168160836199665 | No Hit |
| CTTCACCGTGCCAGACTAGAGTCAAGCTCAACAGGGTCTTCTTTCCCCGC | 34471 | 0.10027942442269294 | No Hit |

## Adapter Content

Produced by FastQC (version 0.11.8)
