# Supplementary material for: Characterization of RNP Networks of PUM1 and PUM2 Post-Transcriptional Regulators in TCam-2 Cells, a Human Male Germ Cell Model
Source: Cells. 2020 Apr 16;9(4):984. doi: 10.3390/cells9040984 (PMC7226987; doi:10.3390/cells9040984)
Supplement: Supplementary file 1 [file cells-09-00984-s001.zip › FASTQC data.zip!/raw_data_QC/RIP/Co-IP_IgG-10_1_fastqc.html]

Co-IP\_IgG-10\_1.fastq.gz FastQC Report 

FastQC Report

Mon 3 Feb 2020  
Co-IP\_IgG-10\_1.fastq.gz

## Summary

- Basic Statistics
- Per base sequence quality
- Per tile sequence quality
- Per sequence quality scores
- Per base sequence content
- Per sequence GC content
- Per base N content
- Sequence Length Distribution
- Sequence Duplication Levels
- Overrepresented sequences
- Adapter Content

## Basic Statistics

| Measure | Value |
| --- | --- |
| Filename | Co-IP\_IgG-10\_1.fastq.gz |
| File type | Conventional base calls |
| Encoding | Sanger / Illumina 1.9 |
| Total Sequences | 39294023 |
| Sequences flagged as poor quality | 0 |
| Sequence length | 101 |
| %GC | 54 |

## Per base sequence quality

## Per tile sequence quality

## Per sequence quality scores

## Per base sequence content

## Per sequence GC content

## Per base N content

## Sequence Length Distribution

## Sequence Duplication Levels

## Overrepresented sequences

| Sequence | Count | Percentage | Possible Source |
| --- | --- | --- | --- |
| CCTCACCCGGCCCGGACACGGACAGGATTGACAGATTGATAGCTCTTTCT | 110527 | 0.2812819649441341 | No Hit |
| CCGCAGGCTCCACTCCTGGTGGTGCCCTTCCGTCAATTCCTTTAAGTTTC | 85194 | 0.21681160007464748 | No Hit |
| GATCGGAAGAGCACACGTCTGAACTCCAGTCACTGACCAATCTCGTATGC | 75819 | 0.19295300967274337 | TruSeq Adapter, Index 4 (100% over 50bp) |
| CTGCCAGTAGCATATGCTTGTCTCAAAGATTAAGCCATGCATGTCTAAGT | 59159 | 0.15055470395586626 | No Hit |
| CTCACCCGGCCCGGACACGGACAGGATTGACAGATTGATAGCTCTTTCTC | 57907 | 0.14736846873632664 | No Hit |
| CTTGTTACGACTTTTACTTCCTCTAGATAGTCAAGTTCGACCGTCTTCTC | 57493 | 0.14631487338417856 | No Hit |
| CGGCGCAAGACGGACCAGAGCGAAAGCATTTGCCAAGAATGTTTTCATTA | 51620 | 0.13136857989827105 | No Hit |
| CTCAGACAGGCGTAGCCCCGGGAGGAACCCGGGGCCGCAAGTGCGTTCGA | 50763 | 0.1291875866209983 | No Hit |
| CGGACACGGACAGGATTGACAGATTGATAGCTCTTTCTCGATTCCGTGGG | 48402 | 0.12317903921418279 | No Hit |
| CGGGGCTGCACGCGCGCTACACTGACTGGCTCAGCGTGTGCCTACCCTAC | 46352 | 0.11796196077963307 | No Hit |
| CTTAGATGTCCGGGGCTGCACGCGCGCTACACTGACTGGCTCAGCGTGTG | 44773 | 0.11394353792687503 | No Hit |
| CCGGAAGCTGCCCGGCGGGTCATGGGAATAACGCCGCCGCATCGCCGGTC | 44105 | 0.1122435338321047 | No Hit |
| CTCGGCTCGTGCGTCGATGAAGAACGCAGCTAGCTGCGAGAATTAATGTG | 40897 | 0.1040794423111118 | No Hit |
| CTCGGGCCTGCTTTGAACACTCTAATTTTTTCAAAGTAAACGCTTCGGGC | 39516 | 0.10056491288764197 | No Hit |
| CTAACACGTGCGCGAGTCGGGGGCTCGCACGAAAGCCGCCGTGGCGCAAT | 39485 | 0.10048602048204633 | No Hit |

## Adapter Content

Produced by FastQC (version 0.11.8)
