# Supplementary material for: Characterization of RNP Networks of PUM1 and PUM2 Post-Transcriptional Regulators in TCam-2 Cells, a Human Male Germ Cell Model
Source: Cells. 2020 Apr 16;9(4):984. doi: 10.3390/cells9040984 (PMC7226987; doi:10.3390/cells9040984)
Supplement: Supplementary file 1 [file cells-09-00984-s001.zip › FASTQC data.zip!/raw_data_QC/RIP/Co-IP_IgG-10_2_fastqc.html]

Co-IP\_IgG-10\_2.fastq.gz FastQC Report 

FastQC Report

Mon 3 Feb 2020  
Co-IP\_IgG-10\_2.fastq.gz

## Summary

- Basic Statistics
- Per base sequence quality
- Per tile sequence quality
- Per sequence quality scores
- Per base sequence content
- Per sequence GC content
- Per base N content
- Sequence Length Distribution
- Sequence Duplication Levels
- Overrepresented sequences
- Adapter Content

## Basic Statistics

| Measure | Value |
| --- | --- |
| Filename | Co-IP\_IgG-10\_2.fastq.gz |
| File type | Conventional base calls |
| Encoding | Sanger / Illumina 1.9 |
| Total Sequences | 39294023 |
| Sequences flagged as poor quality | 0 |
| Sequence length | 101 |
| %GC | 54 |

## Per base sequence quality

## Per tile sequence quality

## Per sequence quality scores

## Per base sequence content

## Per sequence GC content

## Per base N content

## Sequence Length Distribution

## Sequence Duplication Levels

## Overrepresented sequences

| Sequence | Count | Percentage | Possible Source |
| --- | --- | --- | --- |
| CCTCACCCGGCCCGGACACGGACAGGATTGACAGATTGATAGCTCTTTCT | 96908 | 0.24662274972455733 | No Hit |
| CCGCAGGCTCCACTCCTGGTGGTGCCCTTCCGTCAATTCCTTTAAGTTTC | 84554 | 0.21518285363654419 | No Hit |
| GATCGGAAGAGCGTCGTGTAGGGAAAGAGTGTAGATCTCGGTGGTCGCCG | 75494 | 0.19212591187214403 | Illumina Single End PCR Primer 1 (100% over 50bp) |
| CTGCCAGTAGCATATGCTTGTCTCAAAGATTAAGCCATGCATGTCTAAGT | 53276 | 0.1355829613068634 | No Hit |
| CGGCGCAAGACGGACCAGAGCGAAAGCATTTGCCAAGAATGTTTTCATTA | 49346 | 0.12558144021038517 | No Hit |
| CTCACCCGGCCCGGACACGGACAGGATTGACAGATTGATAGCTCTTTCTC | 49015 | 0.12473907291192861 | No Hit |
| CTTGTTACGACTTTTACTTCCTCTAGATAGTCAAGTTCGACCGTCTTCTC | 47263 | 0.12028037953762077 | No Hit |
| CGGACACGGACAGGATTGACAGATTGATAGCTCTTTCTCGATTCCGTGGG | 46698 | 0.11884250182273269 | No Hit |
| CTCGGCTCGTGCGTCGATGAAGAACGCAGCTAGCTGCGAGAATTAATGTG | 45421 | 0.11559264369545466 | No Hit |
| CTCAGACAGGCGTAGCCCCGGGAGGAACCCGGGGCCGCAAGTGCGTTCGA | 43972 | 0.11190505996293634 | No Hit |
| CGGGGCTGCACGCGCGCTACACTGACTGGCTCAGCGTGTGCCTACCCTAC | 41713 | 0.10615609401969353 | No Hit |
| CTTAGATGTCCGGGGCTGCACGCGCGCTACACTGACTGGCTCAGCGTGTG | 40989 | 0.10431357461158915 | No Hit |

## Adapter Content

Produced by FastQC (version 0.11.8)
