# Supplementary material for: Characterization of RNP Networks of PUM1 and PUM2 Post-Transcriptional Regulators in TCam-2 Cells, a Human Male Germ Cell Model
Source: Cells. 2020 Apr 16;9(4):984. doi: 10.3390/cells9040984 (PMC7226987; doi:10.3390/cells9040984)
Supplement: Supplementary file 1 [file cells-09-00984-s001.zip › FASTQC data.zip!/raw_data_QC/RIP/Co-IP_PUM1-5_2_fastqc.html]

Co-IP\_PUM1-5\_2.fastq.gz FastQC Report 

FastQC Report

Mon 3 Feb 2020  
Co-IP\_PUM1-5\_2.fastq.gz

## Summary

- Basic Statistics
- Per base sequence quality
- Per tile sequence quality
- Per sequence quality scores
- Per base sequence content
- Per sequence GC content
- Per base N content
- Sequence Length Distribution
- Sequence Duplication Levels
- Overrepresented sequences
- Adapter Content

## Basic Statistics

| Measure | Value |
| --- | --- |
| Filename | Co-IP\_PUM1-5\_2.fastq.gz |
| File type | Conventional base calls |
| Encoding | Sanger / Illumina 1.9 |
| Total Sequences | 35847251 |
| Sequences flagged as poor quality | 0 |
| Sequence length | 101 |
| %GC | 53 |

## Per base sequence quality

## Per tile sequence quality

## Per sequence quality scores

## Per base sequence content

## Per sequence GC content

## Per base N content

## Sequence Length Distribution

## Sequence Duplication Levels

## Overrepresented sequences

| Sequence | Count | Percentage | Possible Source |
| --- | --- | --- | --- |
| CCTCACCCGGCCCGGACACGGACAGGATTGACAGATTGATAGCTCTTTCT | 151577 | 0.42284134981508065 | No Hit |
| CTCACCCGGCCCGGACACGGACAGGATTGACAGATTGATAGCTCTTTCTC | 95042 | 0.265130511681356 | No Hit |
| CGCGTAACTAGTTAGCATGCCAGAGTCTCGTTCGTTATCGGAATTAACCA | 82822 | 0.23104142630072247 | No Hit |
| AGATCGGAAGAGCGTCGTGTAGGGAAAGAGTGTAGATCTCGGTGGTCGCC | 64935 | 0.18114359731517488 | Illumina Single End PCR Primer 1 (100% over 50bp) |
| GTCGGCATGTATTAGCTCTAGAATTACCACAGTTATCCAAGTAGGAGAGG | 61692 | 0.17209687850262215 | No Hit |
| CTGCCAGTAGCATATGCTTGTCTCAAAGATTAAGCCATGCATGTCTAAGT | 57978 | 0.16173625140739523 | No Hit |
| CTTAGATGTCCGGGGCTGCACGCGCGCTACACTGACTGGCTCAGCGTGTG | 55960 | 0.15610680997547066 | No Hit |
| CAAAGGGCAGGGACTTAATCAACGCAAGCTTATGACCCGCACTTACTGGG | 48652 | 0.13572030948760896 | No Hit |
| CCCGTCGGCATGTATTAGCTCTAGAATTACCACAGTTATCCAAGTAGGAG | 47614 | 0.1328246899601869 | No Hit |
| CCCGCACTTACTGGGAATTCCTCGTTCATGGGGAATAATTGCAATCCCCG | 45117 | 0.12585902333208202 | No Hit |
| CTTGTTACGACTTTTACTTCCTCTAGATAGTCAAGTTCGACCGTCTTCTC | 44143 | 0.12314193911270908 | No Hit |
| CTCCGACTTTCGTTCTTGATTAATGAAAACATTCTTGGCAAATGCTTTCG | 43858 | 0.12234689906905273 | No Hit |
| CTGCTGCCTTCCTTGGATGTGGTAGCCGTTTCTCAGGCTCCCTCTCCGGA | 43579 | 0.121568596710526 | No Hit |
| CTGCGGTATCCAGGCGGCTCGGGCCTGCTTTGAACACTCTAATTTTTTCA | 43574 | 0.12155464863958466 | No Hit |
| CTCTGGTCCGTCTTGCGCCGGTCCAAGAATTTCACCTCTAGCGGCGCAAT | 42390 | 0.1182517454406755 | No Hit |
| CAGGGACTTAATCAACGCAAGCTTATGACCCGCACTTACTGGGAATTCCT | 41764 | 0.1165054469588198 | No Hit |
| CTCGTTCGTTATCGGAATTAACCAGACAAATCGCTCCACCAACTAAGAAC | 41507 | 0.11578851611243496 | No Hit |
| CTTGTCTCAAAGATTAAGCCATGCATGTCTAAGTACGCACGGCCGGTACA | 41422 | 0.11555139890643219 | No Hit |
| CTCGGGCCTGCTTTGAACACTCTAATTTTTTCAAAGTAAACGCTTCGGGC | 40073 | 0.11178820936645882 | No Hit |
| CTCCAATGGATCCTCGTTAAAGGATTTAAAGTGGACTCATTCCAATTACA | 39651 | 0.11061099217900977 | No Hit |
| CTCGTTCATGGGGAATAATTGCAATCCCCGATCCCCATCACGAATGGGGT | 38144 | 0.10640704359729007 | No Hit |
| CCGACTTTCGTTCTTGATTAATGAAAACATTCTTGGCAAATGCTTTCGCT | 38010 | 0.10603323529606216 | No Hit |
| CTAACACGTGCGCGAGTCGGGGGCTCGCACGAAAGCCGCCGTGGCGCAAT | 36988 | 0.1031822495956524 | No Hit |
| GTTGGATCTTGGGAGCGGGCGGGCGGTCCGCCGCGAGGCGAGCCACCGCC | 36714 | 0.10241789530806698 | No Hit |
| CTTCCGTCAATTCCTTTAAGTTTCAGCTTTGCAACCATACTCCCCCCGGA | 36541 | 0.10193529205349665 | No Hit |
| CAAAGATTAAGCCATGCATGTCTAAGTACGCACGGCCGGTACAGTGAAAC | 36173 | 0.10090871403221408 | No Hit |
| CCAGAGTCTCGTTCGTTATCGGAATTAACCAGACAAATCGCTCCACCAAC | 36096 | 0.10069391373971745 | No Hit |

## Adapter Content

Produced by FastQC (version 0.11.8)
