# Supplementary material for: Characterization of RNP Networks of PUM1 and PUM2 Post-Transcriptional Regulators in TCam-2 Cells, a Human Male Germ Cell Model
Source: Cells. 2020 Apr 16;9(4):984. doi: 10.3390/cells9040984 (PMC7226987; doi:10.3390/cells9040984)
Supplement: Supplementary file 1 [file cells-09-00984-s001.zip › FASTQC data.zip!/raw_data_QC/RIP/Co-IP_PUM1-6_1_fastqc.html]

Co-IP\_PUM1-6\_1.fastq.gz FastQC Report 

FastQC Report

Mon 3 Feb 2020  
Co-IP\_PUM1-6\_1.fastq.gz

## Summary

- Basic Statistics
- Per base sequence quality
- Per tile sequence quality
- Per sequence quality scores
- Per base sequence content
- Per sequence GC content
- Per base N content
- Sequence Length Distribution
- Sequence Duplication Levels
- Overrepresented sequences
- Adapter Content

## Basic Statistics

| Measure | Value |
| --- | --- |
| Filename | Co-IP\_PUM1-6\_1.fastq.gz |
| File type | Conventional base calls |
| Encoding | Sanger / Illumina 1.9 |
| Total Sequences | 35843220 |
| Sequences flagged as poor quality | 0 |
| Sequence length | 101 |
| %GC | 53 |

## Per base sequence quality

## Per tile sequence quality

## Per sequence quality scores

## Per base sequence content

## Per sequence GC content

## Per base N content

## Sequence Length Distribution

## Sequence Duplication Levels

## Overrepresented sequences

| Sequence | Count | Percentage | Possible Source |
| --- | --- | --- | --- |
| CCTCACCCGGCCCGGACACGGACAGGATTGACAGATTGATAGCTCTTTCT | 194043 | 0.5413659821857523 | No Hit |
| CTCACCCGGCCCGGACACGGACAGGATTGACAGATTGATAGCTCTTTCTC | 123914 | 0.34571112751588723 | No Hit |
| CGCGTAACTAGTTAGCATGCCAGAGTCTCGTTCGTTATCGGAATTAACCA | 82999 | 0.23156122692101883 | No Hit |
| CTGCCAGTAGCATATGCTTGTCTCAAAGATTAAGCCATGCATGTCTAAGT | 64741 | 0.18062272307008131 | No Hit |
| CTTAGATGTCCGGGGCTGCACGCGCGCTACACTGACTGGCTCAGCGTGTG | 64525 | 0.18002009864069132 | No Hit |
| GTCGGCATGTATTAGCTCTAGAATTACCACAGTTATCCAAGTAGGAGAGG | 60802 | 0.16963319701745547 | No Hit |
| CTGCTGCCTTCCTTGGATGTGGTAGCCGTTTCTCAGGCTCCCTCTCCGGA | 58166 | 0.16227894703656648 | No Hit |
| CCCGTCGGCATGTATTAGCTCTAGAATTACCACAGTTATCCAAGTAGGAG | 55988 | 0.15620248404021736 | No Hit |
| CTGCGGTATCCAGGCGGCTCGGGCCTGCTTTGAACACTCTAATTTTTTCA | 54382 | 0.15172185981058622 | No Hit |
| CTCCGACTTTCGTTCTTGATTAATGAAAACATTCTTGGCAAATGCTTTCG | 53296 | 0.1486919980961532 | No Hit |
| CTTGTCTCAAAGATTAAGCCATGCATGTCTAAGTACGCACGGCCGGTACA | 53207 | 0.1484436945118212 | No Hit |
| CTCTGGTCCGTCTTGCGCCGGTCCAAGAATTTCACCTCTAGCGGCGCAAT | 52107 | 0.1453747738065944 | No Hit |
| CTCCAATGGATCCTCGTTAAAGGATTTAAAGTGGACTCATTCCAATTACA | 52081 | 0.14530223568083447 | No Hit |
| CTTGTTACGACTTTTACTTCCTCTAGATAGTCAAGTTCGACCGTCTTCTC | 50084 | 0.1397307496368909 | No Hit |
| CAAAGGGCAGGGACTTAATCAACGCAAGCTTATGACCCGCACTTACTGGG | 50009 | 0.1395215050433527 | No Hit |
| CTCGGGCCTGCTTTGAACACTCTAATTTTTTCAAAGTAAACGCTTCGGGC | 49074 | 0.1369129224439099 | No Hit |
| CTCGTTCGTTATCGGAATTAACCAGACAAATCGCTCCACCAACTAAGAAC | 47406 | 0.1322593226836205 | No Hit |
| CCCGCACTTACTGGGAATTCCTCGTTCATGGGGAATAATTGCAATCCCCG | 45109 | 0.12585085826552414 | No Hit |
| CAGGGACTTAATCAACGCAAGCTTATGACCCGCACTTACTGGGAATTCCT | 45099 | 0.1258229589863857 | No Hit |
| CTAACACGTGCGCGAGTCGGGGGCTCGCACGAAAGCCGCCGTGGCGCAAT | 44916 | 0.1253124021781525 | No Hit |
| CAAAGATTAAGCCATGCATGTCTAAGTACGCACGGCCGGTACAGTGAAAC | 44229 | 0.12339572170134269 | No Hit |
| CTTCCGTCAATTCCTTTAAGTTTCAGCTTTGCAACCATACTCCCCCCGGA | 43184 | 0.12048024703137722 | No Hit |
| CTTGGACCGGCGCAAGACGGACCAGAGCGAAAGCATTTGCCAAGAATGTT | 41691 | 0.11631488465601025 | No Hit |
| CTCGTTCATGGGGAATAATTGCAATCCCCGATCCCCATCACGAATGGGGT | 41520 | 0.11583780698274318 | No Hit |
| CTCAGTTCCGAAAACCAACAAAATAGAACCGCGGTCCTATTCCATTATTC | 40036 | 0.11169755395860081 | No Hit |
| CTCGGGGGTCGCGTAACTAGTTAGCATGCCAGAGTCTCGTTCGTTATCGG | 38975 | 0.10873744044201386 | No Hit |
| CCGGAAGCTGCCCGGCGGGTCATGGGAATAACGCCGCCGCATCGCCGGTC | 38770 | 0.10816550521967613 | No Hit |
| CCGACTTTCGTTCTTGATTAATGAAAACATTCTTGGCAAATGCTTTCGCT | 37509 | 0.10464740612032067 | No Hit |
| CGCCTGCTGCCTTCCTTGGATGTGGTAGCCGTTTCTCAGGCTCCCTCTCC | 37456 | 0.10449953994088701 | No Hit |
| CCGGTATTTAGCCTTAGATGGAGTTTACCACCCGCTTTGGGCTGCATTCC | 36155 | 0.10086984372497784 | No Hit |

## Adapter Content

Produced by FastQC (version 0.11.8)
