# Supplementary material for: Characterization of RNP Networks of PUM1 and PUM2 Post-Transcriptional Regulators in TCam-2 Cells, a Human Male Germ Cell Model
Source: Cells. 2020 Apr 16;9(4):984. doi: 10.3390/cells9040984 (PMC7226987; doi:10.3390/cells9040984)
Supplement: Supplementary file 1 [file cells-09-00984-s001.zip › FASTQC data.zip!/raw_data_QC/RIP/Co-IP_PUM1-7_2_fastqc.html]

Co-IP\_PUM1-7\_2.fastq.gz FastQC Report 

FastQC Report

Mon 3 Feb 2020  
Co-IP\_PUM1-7\_2.fastq.gz

## Summary

- Basic Statistics
- Per base sequence quality
- Per tile sequence quality
- Per sequence quality scores
- Per base sequence content
- Per sequence GC content
- Per base N content
- Sequence Length Distribution
- Sequence Duplication Levels
- Overrepresented sequences
- Adapter Content

## Basic Statistics

| Measure | Value |
| --- | --- |
| Filename | Co-IP\_PUM1-7\_2.fastq.gz |
| File type | Conventional base calls |
| Encoding | Sanger / Illumina 1.9 |
| Total Sequences | 39312207 |
| Sequences flagged as poor quality | 0 |
| Sequence length | 101 |
| %GC | 53 |

## Per base sequence quality

## Per tile sequence quality

## Per sequence quality scores

## Per base sequence content

## Per sequence GC content

## Per base N content

## Sequence Length Distribution

## Sequence Duplication Levels

## Overrepresented sequences

| Sequence | Count | Percentage | Possible Source |
| --- | --- | --- | --- |
| CCTCACCCGGCCCGGACACGGACAGGATTGACAGATTGATAGCTCTTTCT | 189709 | 0.48257021031660724 | No Hit |
| CTCACCCGGCCCGGACACGGACAGGATTGACAGATTGATAGCTCTTTCTC | 119396 | 0.30371227949628976 | No Hit |
| CGCGTAACTAGTTAGCATGCCAGAGTCTCGTTCGTTATCGGAATTAACCA | 105119 | 0.2673953156585689 | No Hit |
| GTCGGCATGTATTAGCTCTAGAATTACCACAGTTATCCAAGTAGGAGAGG | 76536 | 0.19468761954779085 | No Hit |
| CTGCCAGTAGCATATGCTTGTCTCAAAGATTAAGCCATGCATGTCTAAGT | 75395 | 0.19178521317818661 | No Hit |
| CCCGTCGGCATGTATTAGCTCTAGAATTACCACAGTTATCCAAGTAGGAG | 71051 | 0.18073521031266446 | No Hit |
| CTTAGATGTCCGGGGCTGCACGCGCGCTACACTGACTGGCTCAGCGTGTG | 65053 | 0.16547786289383346 | No Hit |
| CAAAGGGCAGGGACTTAATCAACGCAAGCTTATGACCCGCACTTACTGGG | 60978 | 0.15511212585953263 | No Hit |
| CTGCTGCCTTCCTTGGATGTGGTAGCCGTTTCTCAGGCTCCCTCTCCGGA | 59833 | 0.15219954453333032 | No Hit |
| CTCCAATGGATCCTCGTTAAAGGATTTAAAGTGGACTCATTCCAATTACA | 59159 | 0.15048506434655273 | No Hit |
| CTCCGACTTTCGTTCTTGATTAATGAAAACATTCTTGGCAAATGCTTTCG | 59028 | 0.15015183451796538 | No Hit |
| CCCGCACTTACTGGGAATTCCTCGTTCATGGGGAATAATTGCAATCCCCG | 55032 | 0.13998705287647675 | No Hit |
| CTCGTTCGTTATCGGAATTAACCAGACAAATCGCTCCACCAACTAAGAAC | 54133 | 0.13770023138105678 | No Hit |
| CTTGTTACGACTTTTACTTCCTCTAGATAGTCAAGTTCGACCGTCTTCTC | 51900 | 0.13202006186017487 | No Hit |
| CTGCGGTATCCAGGCGGCTCGGGCCTGCTTTGAACACTCTAATTTTTTCA | 51686 | 0.13147570168217723 | No Hit |
| CTCTGGTCCGTCTTGCGCCGGTCCAAGAATTTCACCTCTAGCGGCGCAAT | 50820 | 0.12927282357869146 | No Hit |
| CTTGTCTCAAAGATTAAGCCATGCATGTCTAAGTACGCACGGCCGGTACA | 50810 | 0.12924738618719625 | No Hit |
| CTCGGGCCTGCTTTGAACACTCTAATTTTTTCAAAGTAAACGCTTCGGGC | 50491 | 0.12843593339849885 | No Hit |
| CAGGGACTTAATCAACGCAAGCTTATGACCCGCACTTACTGGGAATTCCT | 49223 | 0.12521047215690537 | No Hit |
| CTTCCGTCAATTCCTTTAAGTTTCAGCTTTGCAACCATACTCCCCCCGGA | 49000 | 0.12464321832656204 | No Hit |
| CAAAGATTAAGCCATGCATGTCTAAGTACGCACGGCCGGTACAGTGAAAC | 46057 | 0.11715699400951973 | No Hit |
| CTCGTTCATGGGGAATAATTGCAATCCCCGATCCCCATCACGAATGGGGT | 44588 | 0.1134202411988724 | No Hit |
| CCGGTATTTAGCCTTAGATGGAGTTTACCACCCGCTTTGGGCTGCATTCC | 44475 | 0.11313279867497644 | No Hit |
| CTAACACGTGCGCGAGTCGGGGGCTCGCACGAAAGCCGCCGTGGCGCAAT | 44384 | 0.11290131841236999 | No Hit |
| CCGACTTTCGTTCTTGATTAATGAAAACATTCTTGGCAAATGCTTTCGCT | 43646 | 0.11102403892002298 | No Hit |
| GTTGGATCTTGGGAGCGGGCGGGCGGTCCGCCGCGAGGCGAGCCACCGCC | 42879 | 0.10907299099233987 | No Hit |
| CCGGAAGCTGCCCGGCGGGTCATGGGAATAACGCCGCCGCATCGCCGGTC | 42052 | 0.10696931871568544 | No Hit |
| CCAGAGTCTCGTTCGTTATCGGAATTAACCAGACAAATCGCTCCACCAAC | 40880 | 0.10398805643244605 | No Hit |
| CCGTCGGCATGTATTAGCTCTAGAATTACCACAGTTATCCAAGTAGGAGA | 40571 | 0.10320204103524384 | No Hit |
| CTTGGACCGGCGCAAGACGGACCAGAGCGAAAGCATTTGCCAAGAATGTT | 40277 | 0.10245418172528448 | No Hit |

## Adapter Content

Produced by FastQC (version 0.11.8)
