# Supplementary material for: Characterization of RNP Networks of PUM1 and PUM2 Post-Transcriptional Regulators in TCam-2 Cells, a Human Male Germ Cell Model
Source: Cells. 2020 Apr 16;9(4):984. doi: 10.3390/cells9040984 (PMC7226987; doi:10.3390/cells9040984)
Supplement: Supplementary file 1 [file cells-09-00984-s001.zip › FASTQC data.zip!/raw_data_QC/RIP/CO-IP_PUM2-1_1_fastqc.html]

CO-IP\_PUM2-1\_1.fastq.gz FastQC Report 

FastQC Report

Tue 4 Feb 2020  
CO-IP\_PUM2-1\_1.fastq.gz

## Summary

- Basic Statistics
- Per base sequence quality
- Per tile sequence quality
- Per sequence quality scores
- Per base sequence content
- Per sequence GC content
- Per base N content
- Sequence Length Distribution
- Sequence Duplication Levels
- Overrepresented sequences
- Adapter Content

## Basic Statistics

| Measure | Value |
| --- | --- |
| Filename | CO-IP\_PUM2-1\_1.fastq.gz |
| File type | Conventional base calls |
| Encoding | Sanger / Illumina 1.9 |
| Total Sequences | 40474852 |
| Sequences flagged as poor quality | 0 |
| Sequence length | 101 |
| %GC | 52 |

## Per base sequence quality

## Per tile sequence quality

## Per sequence quality scores

## Per base sequence content

## Per sequence GC content

## Per base N content

## Sequence Length Distribution

## Sequence Duplication Levels

## Overrepresented sequences

| Sequence | Count | Percentage | Possible Source |
| --- | --- | --- | --- |
| CCTCACCCGGCCCGGACACGGACAGGATTGACAGATTGATAGCTCTTTCT | 254799 | 0.6295242290200345 | No Hit |
| CTCACCCGGCCCGGACACGGACAGGATTGACAGATTGATAGCTCTTTCTC | 160713 | 0.39706877742258334 | No Hit |
| CGCGTAACTAGTTAGCATGCCAGAGTCTCGTTCGTTATCGGAATTAACCA | 135072 | 0.333718329593892 | No Hit |
| CTGCCAGTAGCATATGCTTGTCTCAAAGATTAAGCCATGCATGTCTAAGT | 115930 | 0.28642476567919256 | No Hit |
| CCCGTCGGCATGTATTAGCTCTAGAATTACCACAGTTATCCAAGTAGGAG | 113139 | 0.2795291258878476 | No Hit |
| CTGCTGCCTTCCTTGGATGTGGTAGCCGTTTCTCAGGCTCCCTCTCCGGA | 100150 | 0.24743759408928784 | No Hit |
| GTCGGCATGTATTAGCTCTAGAATTACCACAGTTATCCAAGTAGGAGAGG | 100099 | 0.2473115899225524 | No Hit |
| CTCCGACTTTCGTTCTTGATTAATGAAAACATTCTTGGCAAATGCTTTCG | 90848 | 0.22445542234471916 | No Hit |
| CTTAGATGTCCGGGGCTGCACGCGCGCTACACTGACTGGCTCAGCGTGTG | 75712 | 0.1870593621935912 | No Hit |
| CTGCGGTATCCAGGCGGCTCGGGCCTGCTTTGAACACTCTAATTTTTTCA | 73513 | 0.18162635900435165 | No Hit |
| CTGCTTTGAACACTCTAATTTTTTCAAAGTAAACGCTTCGGGCCCCGCGG | 68448 | 0.1691124157785679 | No Hit |
| CTTCCGTCAATTCCTTTAAGTTTCAGCTTTGCAACCATACTCCCCCCGGA | 65566 | 0.16199194502304787 | No Hit |
| CTTGTCTCAAAGATTAAGCCATGCATGTCTAAGTACGCACGGCCGGTACA | 65183 | 0.16104567843756415 | No Hit |
| CAAAGATTAAGCCATGCATGTCTAAGTACGCACGGCCGGTACAGTGAAAC | 65058 | 0.16073684469556554 | No Hit |
| CTCTGGTCCGTCTTGCGCCGGTCCAAGAATTTCACCTCTAGCGGCGCAAT | 65000 | 0.16059354583927818 | No Hit |
| CCCGCACTTACTGGGAATTCCTCGTTCATGGGGAATAATTGCAATCCCCG | 62136 | 0.153517547142606 | No Hit |
| CCGTCGGCATGTATTAGCTCTAGAATTACCACAGTTATCCAAGTAGGAGA | 61029 | 0.15078251552346628 | No Hit |
| CAGGGACTTAATCAACGCAAGCTTATGACCCGCACTTACTGGGAATTCCT | 60684 | 0.14993013439555009 | No Hit |
| CTCGTTCGTTATCGGAATTAACCAGACAAATCGCTCCACCAACTAAGAAC | 60632 | 0.1498016595588787 | No Hit |
| CGCCTGCTGCCTTCCTTGGATGTGGTAGCCGTTTCTCAGGCTCCCTCTCC | 60207 | 0.1487516248360834 | No Hit |
| CTCGGGCCTGCTTTGAACACTCTAATTTTTTCAAAGTAAACGCTTCGGGC | 58200 | 0.1437929902745537 | No Hit |
| CTCAGTTCCGAAAACCAACAAAATAGAACCGCGGTCCTATTCCATTATTC | 56695 | 0.1400746320208904 | No Hit |
| CAAAGGGCAGGGACTTAATCAACGCAAGCTTATGACCCGCACTTACTGGG | 56009 | 0.13837975244480202 | No Hit |
| CTCGGGGGTCGCGTAACTAGTTAGCATGCCAGAGTCTCGTTCGTTATCGG | 53663 | 0.13258356077497208 | No Hit |
| CTCCAATGGATCCTCGTTAAAGGATTTAAAGTGGACTCATTCCAATTACA | 52539 | 0.12980652776692056 | No Hit |
| CCGGAAGCTGCCCGGCGGGTCATGGGAATAACGCCGCCGCATCGCCGGTC | 50468 | 0.12468977032948755 | No Hit |
| CCGACTTTCGTTCTTGATTAATGAAAACATTCTTGGCAAATGCTTTCGCT | 49913 | 0.12331854851501373 | No Hit |
| CTCGTTCATGGGGAATAATTGCAATCCCCGATCCCCATCACGAATGGGGT | 49717 | 0.1228342972075599 | No Hit |
| CTTGTTACGACTTTTACTTCCTCTAGATAGTCAAGTTCGACCGTCTTCTC | 47061 | 0.1162721978575734 | No Hit |
| CCTGCTTTGAACACTCTAATTTTTTCAAAGTAAACGCTTCGGGCCCCGCG | 46484 | 0.1148466213045078 | No Hit |
| GTTGGATCTTGGGAGCGGGCGGGCGGTCCGCCGCGAGGCGAGCCACCGCC | 46300 | 0.11439201803628585 | No Hit |
| CGCGCCTGCTGCCTTCCTTGGATGTGGTAGCCGTTTCTCAGGCTCCCTCT | 46142 | 0.1140016521863996 | No Hit |
| CTGCCTTCCTTGGATGTGGTAGCCGTTTCTCAGGCTCCCTCTCCGGAATC | 46090 | 0.11387317734972817 | No Hit |
| CTTAATCATGGCCTCAGTTCCGAAAACCAACAAAATAGAACCGCGGTCCT | 45978 | 0.11359646231689742 | No Hit |
| CTTTGAACACTCTAATTTTTTCAAAGTAAACGCTTCGGGCCCCGCGGGAC | 43784 | 0.10817581247733778 | No Hit |
| CGTCGGCATGTATTAGCTCTAGAATTACCACAGTTATCCAAGTAGGAGAG | 43574 | 0.10765697179078013 | No Hit |
| CCGGTATTTAGCCTTAGATGGAGTTTACCACCCGCTTTGGGCTGCATTCC | 43509 | 0.10749637824494084 | No Hit |
| CTTGGACCGGCGCAAGACGGACCAGAGCGAAAGCATTTGCCAAGAATGTT | 43168 | 0.10665387979676862 | No Hit |
| CTGCAGTTAAAAAGCTCGTAGTTGGATCTTGGGAGCGGGCGGGCGGTCCG | 42926 | 0.10605597767225931 | No Hit |
| CTCGGGCCGATCGCACGCCCCCCGTGGCGGCGACGACCCATTCGAACGTC | 40947 | 0.10116652186893729 | No Hit |

## Adapter Content

Produced by FastQC (version 0.11.8)
