# Supplementary material for: Characterization of RNP Networks of PUM1 and PUM2 Post-Transcriptional Regulators in TCam-2 Cells, a Human Male Germ Cell Model
Source: Cells. 2020 Apr 16;9(4):984. doi: 10.3390/cells9040984 (PMC7226987; doi:10.3390/cells9040984)
Supplement: Supplementary file 1 [file cells-09-00984-s001.zip › FASTQC data.zip!/raw_data_QC/RIP/Co-IP_PUM2-3_1_fastqc.html]

Co-IP\_PUM2-3\_1.fastq.gz FastQC Report 

FastQC Report

Tue 4 Feb 2020  
Co-IP\_PUM2-3\_1.fastq.gz

## Summary

- Basic Statistics
- Per base sequence quality
- Per tile sequence quality
- Per sequence quality scores
- Per base sequence content
- Per sequence GC content
- Per base N content
- Sequence Length Distribution
- Sequence Duplication Levels
- Overrepresented sequences
- Adapter Content

## Basic Statistics

| Measure | Value |
| --- | --- |
| Filename | Co-IP\_PUM2-3\_1.fastq.gz |
| File type | Conventional base calls |
| Encoding | Sanger / Illumina 1.9 |
| Total Sequences | 32747074 |
| Sequences flagged as poor quality | 0 |
| Sequence length | 101 |
| %GC | 53 |

## Per base sequence quality

## Per tile sequence quality

## Per sequence quality scores

## Per base sequence content

## Per sequence GC content

## Per base N content

## Sequence Length Distribution

## Sequence Duplication Levels

## Overrepresented sequences

| Sequence | Count | Percentage | Possible Source |
| --- | --- | --- | --- |
| CCTCACCCGGCCCGGACACGGACAGGATTGACAGATTGATAGCTCTTTCT | 154268 | 0.4710894170269991 | No Hit |
| CTCACCCGGCCCGGACACGGACAGGATTGACAGATTGATAGCTCTTTCTC | 103131 | 0.3149319539205243 | No Hit |
| CCGGTATTTAGCCTTAGATGGAGTTTACCACCCGCTTTGGGCTGCATTCC | 88143 | 0.26916297926342975 | No Hit |
| CCCGTCGGCATGTATTAGCTCTAGAATTACCACAGTTATCCAAGTAGGAG | 75106 | 0.22935178880409288 | No Hit |
| CGCGTAACTAGTTAGCATGCCAGAGTCTCGTTCGTTATCGGAATTAACCA | 72791 | 0.22228245491490323 | No Hit |
| CTGCTGCCTTCCTTGGATGTGGTAGCCGTTTCTCAGGCTCCCTCTCCGGA | 69114 | 0.2110539708066742 | No Hit |
| CTGGATAGTAGGTAGGGACAGTGGGAATCTCGTTCATCCATTCATGCGCG | 66106 | 0.20186841731264296 | No Hit |
| CTGCCAGTAGCATATGCTTGTCTCAAAGATTAAGCCATGCATGTCTAAGT | 64683 | 0.19752299090905037 | No Hit |
| CTCCGACTTTCGTTCTTGATTAATGAAAACATTCTTGGCAAATGCTTTCG | 63664 | 0.19441126251462953 | No Hit |
| CTGCCGTATCGTTCCGCCTGGGCGGGATTCTGACTTAGAGGCGTTCAGTC | 58107 | 0.17744180747263097 | No Hit |
| GTCGGCATGTATTAGCTCTAGAATTACCACAGTTATCCAAGTAGGAGAGG | 57955 | 0.17697764386521983 | No Hit |
| CTTAGATGTCCGGGGCTGCACGCGCGCTACACTGACTGGCTCAGCGTGTG | 56195 | 0.17160311788466964 | No Hit |
| CTAACACGTGCGCGAGTCGGGGGCTCGCACGAAAGCCGCCGTGGCGCAAT | 52798 | 0.16122967200061905 | No Hit |
| GTCGGGTCTGCGAGAGCGCCAGCTATCCTGAGGGAAACTTCGGAGGGAAC | 51850 | 0.15833475687018633 | No Hit |
| CTCCCTTTCGATCGGCCGAGGGCAACGGAGGCCATCGCCCGTCCCTTCGG | 51004 | 0.15575131994999003 | No Hit |
| CTGGGGTCTGATGAGCGTCGGCATCGGGCGCCTTAACCCGGCGTTCGGTT | 47836 | 0.14607717318499966 | No Hit |
| CTCAGTTCCGAAAACCAACAAAATAGAACCGCGGTCCTATTCCATTATTC | 45174 | 0.13794820263941748 | No Hit |
| CTGCTTTGAACACTCTAATTTTTTCAAAGTAAACGCTTCGGGCCCCGCGG | 44847 | 0.1369496401418948 | No Hit |
| CAGAAACCTCCCGTGGAGCAGAAGGGCAAAAGCTCGCTTGATCTTGATTT | 44292 | 0.1352548322332554 | No Hit |
| CTGCTGTCTATATCAACCAACACCTTTTCTGGGGTCTGATGAGCGTCGGC | 44011 | 0.13439674030113347 | No Hit |
| CCCGCACTTACTGGGAATTCCTCGTTCATGGGGAATAATTGCAATCCCCG | 42643 | 0.13021926783443308 | No Hit |
| CTTCCGTCAATTCCTTTAAGTTTCAGCTTTGCAACCATACTCCCCCCGGA | 42281 | 0.12911382555888812 | No Hit |
| CTTGAACTCTCTCTTCAAAGTTCTTTTCAACTTTCCCTTACGGTACTTGT | 42266 | 0.12906801993973568 | No Hit |
| CAGGGACTTAATCAACGCAAGCTTATGACCCGCACTTACTGGGAATTCCT | 41033 | 0.12530279804540703 | No Hit |
| CAAAGGGCAGGGACTTAATCAACGCAAGCTTATGACCCGCACTTACTGGG | 40486 | 0.1236324198003156 | No Hit |
| CGCCTGCTGCCTTCCTTGGATGTGGTAGCCGTTTCTCAGGCTCCCTCTCC | 40339 | 0.12318352473262191 | No Hit |
| CTTGTCTCAAAGATTAAGCCATGCATGTCTAAGTACGCACGGCCGGTACA | 39986 | 0.12210556582856838 | No Hit |
| CTTCCGTACGCCACATGTCCCGCGCCCCGCCGCGGGGCGGGGATTCGGCG | 39444 | 0.12045045612319438 | No Hit |
| CCCAGGCATAGTTCACCATCTTTCGGGTCCTAACACGTGCGCTCGTGCTC | 39403 | 0.12032525409751112 | No Hit |
| CAAAGATTAAGCCATGCATGTCTAAGTACGCACGGCCGGTACAGTGAAAC | 38017 | 0.11609281488782786 | No Hit |
| CTCCGCCACTCCGGATTCGGGGATCTGAACCCGACTCCCTTTCGATCGGC | 37639 | 0.11493851328518695 | No Hit |
| GTTAGTTTTACCCTACTGATGATGTGTTGTTGCCATGGTAATCCTGCTCA | 37515 | 0.11455985350019363 | No Hit |
| CTGCGGTATCCAGGCGGCTCGGGCCTGCTTTGAACACTCTAATTTTTTCA | 37176 | 0.11352464650734903 | No Hit |
| CTCCCGTCCACTCTCGACTGCCGGCGACGGCCGGGTATGGGCCCGACGCT | 36992 | 0.11296276424574606 | No Hit |
| CTGCCTTCCTTGGATGTGGTAGCCGTTTCTCAGGCTCCCTCTCCGGAATC | 35924 | 0.109701404162094 | No Hit |
| CTCGTTCGTTATCGGAATTAACCAGACAAATCGCTCCACCAACTAAGAAC | 35912 | 0.10966475966677205 | No Hit |
| CTTAATCATGGCCTCAGTTCCGAAAACCAACAAAATAGAACCGCGGTCCT | 35786 | 0.10927999246589176 | No Hit |
| CTCGGGCCTGCTTTGAACACTCTAATTTTTTCAAAGTAAACGCTTCGGGC | 34544 | 0.10548728720007168 | No Hit |
| CTCCACTTCGGCCTTCAAAGTTCTCGTTTGAATATTTGCTACTACCACCA | 34533 | 0.10545369641269324 | No Hit |
| CTTTGAACACTCTAATTTTTTCAAAGTAAACGCTTCGGGCCCCGCGGGAC | 34113 | 0.10417113907642557 | No Hit |
| CTCTGGTCCGTCTTGCGCCGGTCCAAGAATTTCACCTCTAGCGGCGCAAT | 33844 | 0.10334969163962558 | No Hit |
| GTCAAAGTGAAGAAATTCAATGAAGCGCGGGTAAACGGCGGGAGTAACTA | 33800 | 0.10321532849011182 | No Hit |
| CTTCACCGTGCCAGACTAGAGTCAAGCTCAACAGGGTCTTCTTTCCCCGC | 33687 | 0.10287025949249695 | No Hit |
| CCGACATCGAAGGATCAAAAAGCGACGTCGCTATGAACGCTTGGCCGCCA | 33193 | 0.1013617277684107 | No Hit |
| CTCGTTCATGGGGAATAATTGCAATCCCCGATCCCCATCACGAATGGGGT | 33182 | 0.10132813698103225 | No Hit |
| CCCGAAGTTACGGATCCGGCTTGCCGACTTCCCTTACCTACATTGTTCCA | 32895 | 0.10045172280124935 | No Hit |

## Adapter Content

Produced by FastQC (version 0.11.8)
