# Supplementary material for: Characterization of RNP Networks of PUM1 and PUM2 Post-Transcriptional Regulators in TCam-2 Cells, a Human Male Germ Cell Model
Source: Cells. 2020 Apr 16;9(4):984. doi: 10.3390/cells9040984 (PMC7226987; doi:10.3390/cells9040984)
Supplement: Supplementary file 1 [file cells-09-00984-s001.zip › FASTQC data.zip!/raw_data_QC/RIP/Co-IP_PUM2-3_2_fastqc.html]

Co-IP\_PUM2-3\_2.fastq.gz FastQC Report 

FastQC Report

Tue 4 Feb 2020  
Co-IP\_PUM2-3\_2.fastq.gz

## Summary

- Basic Statistics
- Per base sequence quality
- Per tile sequence quality
- Per sequence quality scores
- Per base sequence content
- Per sequence GC content
- Per base N content
- Sequence Length Distribution
- Sequence Duplication Levels
- Overrepresented sequences
- Adapter Content

## Basic Statistics

| Measure | Value |
| --- | --- |
| Filename | Co-IP\_PUM2-3\_2.fastq.gz |
| File type | Conventional base calls |
| Encoding | Sanger / Illumina 1.9 |
| Total Sequences | 32747074 |
| Sequences flagged as poor quality | 0 |
| Sequence length | 101 |
| %GC | 53 |

## Per base sequence quality

## Per tile sequence quality

## Per sequence quality scores

## Per base sequence content

## Per sequence GC content

## Per base N content

## Sequence Length Distribution

## Sequence Duplication Levels

## Overrepresented sequences

| Sequence | Count | Percentage | Possible Source |
| --- | --- | --- | --- |
| CCTCACCCGGCCCGGACACGGACAGGATTGACAGATTGATAGCTCTTTCT | 122523 | 0.37414945836076835 | No Hit |
| CGCGTAACTAGTTAGCATGCCAGAGTCTCGTTCGTTATCGGAATTAACCA | 81789 | 0.24975971899046612 | No Hit |
| CTCACCCGGCCCGGACACGGACAGGATTGACAGATTGATAGCTCTTTCTC | 80159 | 0.24478217504257022 | No Hit |
| CCGGTATTTAGCCTTAGATGGAGTTTACCACCCGCTTTGGGCTGCATTCC | 79482 | 0.24271481476482448 | No Hit |
| CCCGTCGGCATGTATTAGCTCTAGAATTACCACAGTTATCCAAGTAGGAG | 74764 | 0.22830742068741774 | No Hit |
| CTGCCGTATCGTTCCGCCTGGGCGGGATTCTGACTTAGAGGCGTTCAGTC | 64981 | 0.19843299587621174 | No Hit |
| CTGGATAGTAGGTAGGGACAGTGGGAATCTCGTTCATCCATTCATGCGCG | 64411 | 0.1966923823484199 | No Hit |
| CTGCTGCCTTCCTTGGATGTGGTAGCCGTTTCTCAGGCTCCCTCTCCGGA | 62165 | 0.1898337543073314 | No Hit |
| CTCCGACTTTCGTTCTTGATTAATGAAAACATTCTTGGCAAATGCTTTCG | 61923 | 0.18909475698500575 | No Hit |
| CTGCCAGTAGCATATGCTTGTCTCAAAGATTAAGCCATGCATGTCTAAGT | 59433 | 0.18149102420570462 | No Hit |
| GTCGGCATGTATTAGCTCTAGAATTACCACAGTTATCCAAGTAGGAGAGG | 54946 | 0.1677890366632451 | No Hit |
| CTTAGATGTCCGGGGCTGCACGCGCGCTACACTGACTGGCTCAGCGTGTG | 49636 | 0.15157384748328964 | No Hit |
| GTCGGGTCTGCGAGAGCGCCAGCTATCCTGAGGGAAACTTCGGAGGGAAC | 46603 | 0.14231195129067103 | No Hit |
| CTCCCTTTCGATCGGCCGAGGGCAACGGAGGCCATCGCCCGTCCCTTCGG | 44766 | 0.13670228979847177 | No Hit |
| CTAACACGTGCGCGAGTCGGGGGCTCGCACGAAAGCCGCCGTGGCGCAAT | 44094 | 0.1346501980604435 | No Hit |
| CAGAAACCTCCCGTGGAGCAGAAGGGCAAAAGCTCGCTTGATCTTGATTT | 42167 | 0.12876570285332975 | No Hit |
| CCCGCACTTACTGGGAATTCCTCGTTCATGGGGAATAATTGCAATCCCCG | 41695 | 0.12732435270400036 | No Hit |
| CAGGGACTTAATCAACGCAAGCTTATGACCCGCACTTACTGGGAATTCCT | 40495 | 0.12365990317180703 | No Hit |
| CTTCCGTCAATTCCTTTAAGTTTCAGCTTTGCAACCATACTCCCCCCGGA | 40340 | 0.12318657844056541 | No Hit |
| CTTGAACTCTCTCTTCAAAGTTCTTTTCAACTTTCCCTTACGGTACTTGT | 39938 | 0.12195898784728065 | No Hit |
| CAAAGGGCAGGGACTTAATCAACGCAAGCTTATGACCCGCACTTACTGGG | 39698 | 0.12122609794084198 | No Hit |
| CTGGGGTCTGATGAGCGTCGGCATCGGGCGCCTTAACCCGGCGTTCGGTT | 39543 | 0.12075277320960034 | No Hit |
| CCCAGGCATAGTTCACCATCTTTCGGGTCCTAACACGTGCGCTCGTGCTC | 38838 | 0.11859990910943675 | No Hit |
| CTGCTGTCTATATCAACCAACACCTTTTCTGGGGTCTGATGAGCGTCGGC | 38754 | 0.11834339764218323 | No Hit |
| CTGCTTTGAACACTCTAATTTTTTCAAAGTAAACGCTTCGGGCCCCGCGG | 38233 | 0.11675241580362264 | No Hit |
| CTCAGTTCCGAAAACCAACAAAATAGAACCGCGGTCCTATTCCATTATTC | 37279 | 0.11383917842552894 | No Hit |
| CTTCCGTACGCCACATGTCCCGCGCCCCGCCGCGGGGCGGGGATTCGGCG | 36484 | 0.11141148061045086 | No Hit |
| CTCGTTCGTTATCGGAATTAACCAGACAAATCGCTCCACCAACTAAGAAC | 35807 | 0.10934412033270514 | No Hit |
| CAAAGATTAAGCCATGCATGTCTAAGTACGCACGGCCGGTACAGTGAAAC | 35292 | 0.1077714607418055 | No Hit |
| CTTGTCTCAAAGATTAAGCCATGCATGTCTAAGTACGCACGGCCGGTACA | 34935 | 0.106681287005978 | No Hit |
| CGCCTGCTGCCTTCCTTGGATGTGGTAGCCGTTTCTCAGGCTCCCTCTCC | 34931 | 0.10666907217420402 | No Hit |
| CTCCGCCACTCCGGATTCGGGGATCTGAACCCGACTCCCTTTCGATCGGC | 34889 | 0.10654081644057725 | No Hit |
| CTCCCGTCCACTCTCGACTGCCGGCGACGGCCGGGTATGGGCCCGACGCT | 34246 | 0.10457728223291034 | No Hit |
| CTGCCTTCCTTGGATGTGGTAGCCGTTTCTCAGGCTCCCTCTCCGGAATC | 33208 | 0.1014075333875631 | No Hit |

## Adapter Content

Produced by FastQC (version 0.11.8)
