# Supplementary material for: Characterization of RNP Networks of PUM1 and PUM2 Post-Transcriptional Regulators in TCam-2 Cells, a Human Male Germ Cell Model
Source: Cells. 2020 Apr 16;9(4):984. doi: 10.3390/cells9040984 (PMC7226987; doi:10.3390/cells9040984)
Supplement: Supplementary file 1 [file cells-09-00984-s001.zip › FASTQC data.zip!/raw_data_QC/RIP/Co-IP_PUM2-4_1_fastqc.html]

Co-IP\_PUM2-4\_1.fastq.gz FastQC Report 

FastQC Report

Tue 4 Feb 2020  
Co-IP\_PUM2-4\_1.fastq.gz

## Summary

- Basic Statistics
- Per base sequence quality
- Per tile sequence quality
- Per sequence quality scores
- Per base sequence content
- Per sequence GC content
- Per base N content
- Sequence Length Distribution
- Sequence Duplication Levels
- Overrepresented sequences
- Adapter Content

## Basic Statistics

| Measure | Value |
| --- | --- |
| Filename | Co-IP\_PUM2-4\_1.fastq.gz |
| File type | Conventional base calls |
| Encoding | Sanger / Illumina 1.9 |
| Total Sequences | 38530840 |
| Sequences flagged as poor quality | 0 |
| Sequence length | 101 |
| %GC | 53 |

## Per base sequence quality

## Per tile sequence quality

## Per sequence quality scores

## Per base sequence content

## Per sequence GC content

## Per base N content

## Sequence Length Distribution

## Sequence Duplication Levels

## Overrepresented sequences

| Sequence | Count | Percentage | Possible Source |
| --- | --- | --- | --- |
| CCTCACCCGGCCCGGACACGGACAGGATTGACAGATTGATAGCTCTTTCT | 146185 | 0.3793973866129054 | No Hit |
| CTGGATAGTAGGTAGGGACAGTGGGAATCTCGTTCATCCATTCATGCGCG | 102127 | 0.2650526175915189 | No Hit |
| CTCACCCGGCCCGGACACGGACAGGATTGACAGATTGATAGCTCTTTCTC | 97065 | 0.2519150893154678 | No Hit |
| CCGGTATTTAGCCTTAGATGGAGTTTACCACCCGCTTTGGGCTGCATTCC | 95649 | 0.24824011103832672 | No Hit |
| CTTGAACTCTCTCTTCAAAGTTCTTTTCAACTTTCCCTTACGGTACTTGT | 90332 | 0.23444077523355317 | No Hit |
| CTGCTGCCTTCCTTGGATGTGGTAGCCGTTTCTCAGGCTCCCTCTCCGGA | 87745 | 0.2277266729715729 | No Hit |
| GTCGGGTCTGCGAGAGCGCCAGCTATCCTGAGGGAAACTTCGGAGGGAAC | 86272 | 0.223903761246835 | No Hit |
| CGCGTAACTAGTTAGCATGCCAGAGTCTCGTTCGTTATCGGAATTAACCA | 84704 | 0.21983429377610245 | No Hit |
| CTTAGATGTCCGGGGCTGCACGCGCGCTACACTGACTGGCTCAGCGTGTG | 84586 | 0.2195280455863407 | No Hit |
| CCCGTCGGCATGTATTAGCTCTAGAATTACCACAGTTATCCAAGTAGGAG | 81809 | 0.21232083183237116 | No Hit |
| GTCGGCATGTATTAGCTCTAGAATTACCACAGTTATCCAAGTAGGAGAGG | 78282 | 0.203167125346865 | No Hit |
| CAAAGGGCAGGGACTTAATCAACGCAAGCTTATGACCCGCACTTACTGGG | 72388 | 0.1878702878006293 | No Hit |
| CAGAAACCTCCCGTGGAGCAGAAGGGCAAAAGCTCGCTTGATCTTGATTT | 69105 | 0.17934984028378306 | No Hit |
| CTGCCTTCCTTGGATGTGGTAGCCGTTTCTCAGGCTCCCTCTCCGGAATC | 68466 | 0.1776914284765139 | No Hit |
| CTTGTCTCAAAGATTAAGCCATGCATGTCTAAGTACGCACGGCCGGTACA | 68400 | 0.17752013711613865 | No Hit |
| CAAAGATTAAGCCATGCATGTCTAAGTACGCACGGCCGGTACAGTGAAAC | 64896 | 0.16842612307439964 | No Hit |
| CTGCCGTATCGTTCCGCCTGGGCGGGATTCTGACTTAGAGGCGTTCAGTC | 63553 | 0.16494060342312808 | No Hit |
| CGCCTGCTGCCTTCCTTGGATGTGGTAGCCGTTTCTCAGGCTCCCTCTCC | 60882 | 0.1580084939752157 | No Hit |
| CAGGGACTTAATCAACGCAAGCTTATGACCCGCACTTACTGGGAATTCCT | 56733 | 0.14724049618435517 | No Hit |
| CTCCCTTTCGATCGGCCGAGGGCAACGGAGGCCATCGCCCGTCCCTTCGG | 55107 | 0.14302049994238383 | No Hit |
| CTGGGGTCTGATGAGCGTCGGCATCGGGCGCCTTAACCCGGCGTTCGGTT | 54355 | 0.1410688165635631 | No Hit |
| CTTTGAACACTCTAATTTTTTCAAAGTAAACGCTTCGGGCCCCGCGGGAC | 54130 | 0.14048486874410213 | No Hit |
| CTTGGCTGTGGTTTCGCTGGATAGTAGGTAGGGACAGTGGGAATCTCGTT | 53541 | 0.13895622311893538 | No Hit |
| CTTAATCATGGCCTCAGTTCCGAAAACCAACAAAATAGAACCGCGGTCCT | 53176 | 0.13800892998958755 | No Hit |
| CTCCGCCACTCCGGATTCGGGGATCTGAACCCGACTCCCTTTCGATCGGC | 50230 | 0.13036310654011177 | No Hit |
| CGGGGGTGCGTCGGGTCTGCGAGAGCGCCAGCTATCCTGAGGGAAACTTC | 48546 | 0.12599258152690157 | No Hit |
| CTAACACGTGCGCGAGTCGGGGGCTCGCACGAAAGCCGCCGTGGCGCAAT | 48537 | 0.12596922361412313 | No Hit |
| CGAAGCCAGAGGAAACTCTGGTGGAGGTCCGTAGCGGTCCTGACGTGCAA | 48357 | 0.12550206535855435 | No Hit |
| CCGACTTTCGTTCTTGATTAATGAAAACATTCTTGGCAAATGCTTTCGCT | 48310 | 0.12538008514737803 | No Hit |
| CTCCACTTCGGCCTTCAAAGTTCTCGTTTGAATATTTGCTACTACCACCA | 48139 | 0.1249362848045877 | No Hit |
| GATCGGAAGAGCACACGTCTGAACTCCAGTCACGCCAATATCTCGTATGC | 46959 | 0.12187380290697011 | TruSeq Adapter, Index 6 (100% over 50bp) |
| GTTAGTTTTACCCTACTGATGATGTGTTGTTGCCATGGTAATCCTGCTCA | 46913 | 0.12175441801943587 | No Hit |
| CCACTCTCGACTGCCGGCGACGGCCGGGTATGGGCCCGACGCTCCAGCGC | 45037 | 0.1168855908669523 | No Hit |
| CGGGTCTGCGAGAGCGCCAGCTATCCTGAGGGAAACTTCGGAGGGAACCA | 45027 | 0.11685963763053181 | No Hit |
| CTCTGGTGGAGGTCCGTAGCGGTCCTGACGTGCAAATCGGTCGTCCGACC | 45011 | 0.11681811245225902 | No Hit |
| CGCGCCTGCTGCCTTCCTTGGATGTGGTAGCCGTTTCTCAGGCTCCCTCT | 44104 | 0.11446415390892076 | No Hit |
| GGAAACTCTGGTGGAGGTCCGTAGCGGTCCTGACGTGCAAATCGGTCGTC | 43082 | 0.11181173314674686 | No Hit |
| CGGGCTTCTTACCCATTTAAAGTTTGAGAATAGGTTGAGATCGTTTCGGC | 42838 | 0.11117847417808696 | No Hit |
| CTTGGACCGGCGCAAGACGGACCAGAGCGAAAGCATTTGCCAAGAATGTT | 42765 | 0.11098901555221741 | No Hit |
| CGCCACTCCGGATTCGGGGATCTGAACCCGACTCCCTTTCGATCGGCCGA | 42436 | 0.11013515407398333 | No Hit |
| CGGCCTTCAAAGTTCTCGTTTGAATATTTGCTACTACCACCAAGATCTGC | 41998 | 0.10899840231876595 | No Hit |
| CTCGGGCCTGCTTTGAACACTCTAATTTTTTCAAAGTAAACGCTTCGGGC | 41420 | 0.10749830525366175 | No Hit |
| CTCCGACTTTCGTTCTTGATTAATGAAAACATTCTTGGCAAATGCTTTCG | 41273 | 0.10711679267828057 | No Hit |
| GTCTGCGAGAGCGCCAGCTATCCTGAGGGAAACTTCGGAGGGAACCAGCT | 41026 | 0.1064757477386945 | No Hit |
| CTGCTGTCTATATCAACCAACACCTTTTCTGGGGTCTGATGAGCGTCGGC | 40852 | 0.10602416142497802 | No Hit |
| CTTCCTTGGATGTGGTAGCCGTTTCTCAGGCTCCCTCTCCGGAATCGAAC | 40743 | 0.1057412711479947 | No Hit |
| CCCAGGCATAGTTCACCATCTTTCGGGTCCTAACACGTGCGCTCGTGCTC | 40327 | 0.1046616165129024 | No Hit |
| GTCAAAGTGAAGAAATTCAATGAAGCGCGGGTAAACGGCGGGAGTAACTA | 39984 | 0.10377142050367964 | No Hit |
| CTTCCGTCAATTCCTTTAAGTTTCAGCTTTGCAACCATACTCCCCCCGGA | 39847 | 0.10341586116471896 | No Hit |
| CTCGTTCGTTATCGGAATTAACCAGACAAATCGCTCCACCAACTAAGAAC | 39843 | 0.10340547987015077 | No Hit |
| CTTTTCTGACACCTCCTGCTTAAAACCCAAAAGGTCAGAAGGATCGTGAG | 39584 | 0.10273329104686013 | No Hit |
| CGCCCATCTCTCAGGACCGACTGACCCATGTTCAACTGCTGTTCACATGG | 39292 | 0.10197545654338187 | No Hit |
| CCGAAGTTTCCCTCAGGATAGCTGGCGCTCTCGCAGACCCGACGCACCCC | 39267 | 0.10191057345233065 | No Hit |
| CCGACATCGAAGGATCAAAAAGCGACGTCGCTATGAACGCTTGGCCGCCA | 39113 | 0.10151089361145514 | No Hit |
| CTGAATTTAAGCATATTAGTCAGCGGAGGAAAAGAAACTAACCAGGATTC | 38683 | 0.10039490444537416 | No Hit |
| GTCGGCATCGGGCGCCTTAACCCGGCGTTCGGTTCATCCCGCAGCGCCAG | 38638 | 0.10027811488148196 | No Hit |

## Adapter Content

Produced by FastQC (version 0.11.8)
