# Supplementary material for: Characterization of RNP Networks of PUM1 and PUM2 Post-Transcriptional Regulators in TCam-2 Cells, a Human Male Germ Cell Model
Source: Cells. 2020 Apr 16;9(4):984. doi: 10.3390/cells9040984 (PMC7226987; doi:10.3390/cells9040984)
Supplement: Supplementary file 1 [file cells-09-00984-s001.zip › FASTQC data.zip!/raw_data_QC/RIP/Co-IP_PUM2-4_2_fastqc.html]

Co-IP\_PUM2-4\_2.fastq.gz FastQC Report 

FastQC Report

Tue 4 Feb 2020  
Co-IP\_PUM2-4\_2.fastq.gz

## Summary

- Basic Statistics
- Per base sequence quality
- Per tile sequence quality
- Per sequence quality scores
- Per base sequence content
- Per sequence GC content
- Per base N content
- Sequence Length Distribution
- Sequence Duplication Levels
- Overrepresented sequences
- Adapter Content

## Basic Statistics

| Measure | Value |
| --- | --- |
| Filename | Co-IP\_PUM2-4\_2.fastq.gz |
| File type | Conventional base calls |
| Encoding | Sanger / Illumina 1.9 |
| Total Sequences | 38530840 |
| Sequences flagged as poor quality | 0 |
| Sequence length | 101 |
| %GC | 53 |

## Per base sequence quality

## Per tile sequence quality

## Per sequence quality scores

## Per base sequence content

## Per sequence GC content

## Per base N content

## Sequence Length Distribution

## Sequence Duplication Levels

## Overrepresented sequences

| Sequence | Count | Percentage | Possible Source |
| --- | --- | --- | --- |
| CCTCACCCGGCCCGGACACGGACAGGATTGACAGATTGATAGCTCTTTCT | 124343 | 0.3227103276232753 | No Hit |
| CTGGATAGTAGGTAGGGACAGTGGGAATCTCGTTCATCCATTCATGCGCG | 94427 | 0.24506862554774306 | No Hit |
| CGCGTAACTAGTTAGCATGCCAGAGTCTCGTTCGTTATCGGAATTAACCA | 91291 | 0.23692969060627797 | No Hit |
| CCGGTATTTAGCCTTAGATGGAGTTTACCACCCGCTTTGGGCTGCATTCC | 90531 | 0.23495724463832088 | No Hit |
| CTTGAACTCTCTCTTCAAAGTTCTTTTCAACTTTCCCTTACGGTACTTGT | 88794 | 0.2304491674720821 | No Hit |
| CCCGTCGGCATGTATTAGCTCTAGAATTACCACAGTTATCCAAGTAGGAG | 84473 | 0.21923477401478922 | No Hit |
| GTCGGGTCTGCGAGAGCGCCAGCTATCCTGAGGGAAACTTCGGAGGGAAC | 80411 | 0.20869256938078692 | No Hit |
| CTCACCCGGCCCGGACACGGACAGGATTGACAGATTGATAGCTCTTTCTC | 78661 | 0.2041507530072015 | No Hit |
| CTGCTGCCTTCCTTGGATGTGGTAGCCGTTTCTCAGGCTCCCTCTCCGGA | 76305 | 0.1980361705065345 | No Hit |
| GTCGGCATGTATTAGCTCTAGAATTACCACAGTTATCCAAGTAGGAGAGG | 75442 | 0.1957964062034464 | No Hit |
| CTTAGATGTCCGGGGCTGCACGCGCGCTACACTGACTGGCTCAGCGTGTG | 74511 | 0.19338015989269894 | No Hit |
| CAAAGGGCAGGGACTTAATCAACGCAAGCTTATGACCCGCACTTACTGGG | 73523 | 0.19081598013435472 | No Hit |
| CTGCCGTATCGTTCCGCCTGGGCGGGATTCTGACTTAGAGGCGTTCAGTC | 70962 | 0.1841693562870677 | No Hit |
| CAGAAACCTCCCGTGGAGCAGAAGGGCAAAAGCTCGCTTGATCTTGATTT | 65618 | 0.17029994674395887 | No Hit |
| CTGCCTTCCTTGGATGTGGTAGCCGTTTCTCAGGCTCCCTCTCCGGAATC | 62216 | 0.1614706557137088 | No Hit |
| CAAAGATTAAGCCATGCATGTCTAAGTACGCACGGCCGGTACAGTGAAAC | 58929 | 0.15293982690229435 | No Hit |
| CTTGTCTCAAAGATTAAGCCATGCATGTCTAAGTACGCACGGCCGGTACA | 58912 | 0.15289570640037953 | No Hit |
| CAGGGACTTAATCAACGCAAGCTTATGACCCGCACTTACTGGGAATTCCT | 57069 | 0.14811252492808358 | No Hit |
| CTCCCTTTCGATCGGCCGAGGGCAACGGAGGCCATCGCCCGTCCCTTCGG | 51368 | 0.13331658484476333 | No Hit |
| CGCCTGCTGCCTTCCTTGGATGTGGTAGCCGTTTCTCAGGCTCCCTCTCC | 51278 | 0.13308300571697892 | No Hit |
| GATCGGAAGAGCGTCGTGTAGGGAAAGAGTGTAGATCTCGGTGGTCGCCG | 49627 | 0.12879812638395632 | Illumina Single End PCR Primer 1 (100% over 50bp) |
| CTCCGCCACTCCGGATTCGGGGATCTGAACCCGACTCCCTTTCGATCGGC | 48607 | 0.12615089626906653 | No Hit |
| CTTGGCTGTGGTTTCGCTGGATAGTAGGTAGGGACAGTGGGAATCTCGTT | 47140 | 0.12234355648618093 | No Hit |
| CCGACTTTCGTTCTTGATTAATGAAAACATTCTTGGCAAATGCTTTCGCT | 46932 | 0.1218037291686348 | No Hit |
| CTCCACTTCGGCCTTCAAAGTTCTCGTTTGAATATTTGCTACTACCACCA | 45867 | 0.1190397094898528 | No Hit |
| CTGGGGTCTGATGAGCGTCGGCATCGGGCGCCTTAACCCGGCGTTCGGTT | 44656 | 0.11589677255933169 | No Hit |
| CCACTCTCGACTGCCGGCGACGGCCGGGTATGGGCCCGACGCTCCAGCGC | 44273 | 0.114902763604427 | No Hit |
| CTTAATCATGGCCTCAGTTCCGAAAACCAACAAAATAGAACCGCGGTCCT | 43628 | 0.11322877985530551 | No Hit |
| CGGGTCTGCGAGAGCGCCAGCTATCCTGAGGGAAACTTCGGAGGGAACCA | 42619 | 0.11061009830047827 | No Hit |
| CGAAGCCAGAGGAAACTCTGGTGGAGGTCCGTAGCGGTCCTGACGTGCAA | 42405 | 0.11005469904107983 | No Hit |
| CTTTGAACACTCTAATTTTTTCAAAGTAAACGCTTCGGGCCCCGCGGGAC | 42194 | 0.10950708575260752 | No Hit |
| CTAACACGTGCGCGAGTCGGGGGCTCGCACGAAAGCCGCCGTGGCGCAAT | 42128 | 0.1093357943922323 | No Hit |
| GTTAGTTTTACCCTACTGATGATGTGTTGTTGCCATGGTAATCCTGCTCA | 42087 | 0.10922938612290829 | No Hit |
| CGCCACTCCGGATTCGGGGATCTGAACCCGACTCCCTTTCGATCGGCCGA | 42070 | 0.10918526562099347 | No Hit |
| CTCTGGTGGAGGTCCGTAGCGGTCCTGACGTGCAAATCGGTCGTCCGACC | 39919 | 0.10360272446694647 | No Hit |
| GGAAACTCTGGTGGAGGTCCGTAGCGGTCCTGACGTGCAAATCGGTCGTC | 39359 | 0.10214934322739914 | No Hit |
| CTTTTCTGACACCTCCTGCTTAAAACCCAAAAGGTCAGAAGGATCGTGAG | 39315 | 0.10203514898714901 | No Hit |
| CTTCCGTCAATTCCTTTAAGTTTCAGCTTTGCAACCATACTCCCCCCGGA | 39131 | 0.10155760943701203 | No Hit |
| CCGAAGTTTCCCTCAGGATAGCTGGCGCTCTCGCAGACCCGACGCACCCC | 38945 | 0.10107487923959094 | No Hit |
| GTCTGCGAGAGCGCCAGCTATCCTGAGGGAAACTTCGGAGGGAACCAGCT | 38847 | 0.10082053752267016 | No Hit |

## Adapter Content

Produced by FastQC (version 0.11.8)
