# Supplementary material for: Characterization of RNP Networks of PUM1 and PUM2 Post-Transcriptional Regulators in TCam-2 Cells, a Human Male Germ Cell Model
Source: Cells. 2020 Apr 16;9(4):984. doi: 10.3390/cells9040984 (PMC7226987; doi:10.3390/cells9040984)
Supplement: Supplementary file 1 [file cells-09-00984-s001.zip › FASTQC data.zip!/raw_data_QC/siRNA/siPUM1-3_1_fastqc.html]

siPUM1-3\_1.fastq.gz FastQC Report 

FastQC Report

Mon 3 Feb 2020  
siPUM1-3\_1.fastq.gz

## Summary

- Basic Statistics
- Per base sequence quality
- Per tile sequence quality
- Per sequence quality scores
- Per base sequence content
- Per sequence GC content
- Per base N content
- Sequence Length Distribution
- Sequence Duplication Levels
- Overrepresented sequences
- Adapter Content

## Basic Statistics

| Measure | Value |
| --- | --- |
| Filename | siPUM1-3\_1.fastq.gz |
| File type | Conventional base calls |
| Encoding | Sanger / Illumina 1.9 |
| Total Sequences | 58944636 |
| Sequences flagged as poor quality | 0 |
| Sequence length | 101 |
| %GC | 52 |

## Per base sequence quality

## Per tile sequence quality

## Per sequence quality scores

## Per base sequence content

## Per sequence GC content

## Per base N content

## Sequence Length Distribution

## Sequence Duplication Levels

## Overrepresented sequences

| Sequence | Count | Percentage | Possible Source |
| --- | --- | --- | --- |
| GATCGGAAGAGCACACGTCTGAACTCCAGTCACTGACCAATCTCGTATGC | 528750 | 0.8970281876030247 | TruSeq Adapter, Index 4 (100% over 50bp) |
| AGATCGGAAGAGCACACGTCTGAACTCCAGTCACTGACCAATCTCGTATG | 71547 | 0.12138000139656474 | TruSeq Adapter, Index 4 (100% over 49bp) |

## Adapter Content

Produced by FastQC (version 0.11.8)
