# Supplementary material for: Characterization of RNP Networks of PUM1 and PUM2 Post-Transcriptional Regulators in TCam-2 Cells, a Human Male Germ Cell Model
Source: Cells. 2020 Apr 16;9(4):984. doi: 10.3390/cells9040984 (PMC7226987; doi:10.3390/cells9040984)
Supplement: Supplementary file 1 [file cells-09-00984-s001.zip › FINAL Supplementary Files.zip!/FINAL Supplementary Files/Detailed description of Supplementary Tables.pdf]

Table S1 List of siRNAs used for PUM1 and PUM2 knockdown

This table contains triplets of double stranded siRNA sequences used for PUM1 and PUM2 knockdown experiments.

Table S2 List of primers for RT-qPCR

This table contains names, primers sequences, primer lengths, amplicon lengths, annealing temperatures and FPKM values of the genes tested by RT-qPCR.

Table S3 Mass spectrometry protein identification data

This table, contains data obtained by mass spectrometry analysis from three biological replicates (each biological replicate being presented in a separate sheet) of our Co-IP/MS analysis with or without RNase-A treatment. Each sheet contains 23 columns. Please note, that not all columns are visible on the first screen.

Table S4 PUM1, PUM2 and common protein interactors

This table contains all proteins identified applying Co-IP/MS analysis. The 1st sheet contains list of proteins found to interact with PUMs independently of RNA presence (RNA independent binding). The 2nd sheet contains a list of proteins found to interact with PUMs via RNA (RNA dependent binding). The 3rd sheet contains a list of all PUM interacting proteins. FPKM values evaluated in TCam-2 cells for each mRNA encoding those proteins is highlighted (yellow brackets). The 4th sheet, contains only PUM interacting RBPs together with their RNA-binding motifs (motif assignment to RBPs). This data was used for building our regulon model.

Table S5 More than average cofactor motif enrichment in PUM1 and PUM2 regulated mRNAs

This table contains list of mRNAs that reached the threshold of more than average motif enrichment of particular RBP (listed in first grey highlighted line) bound to PUM1 or PUM2 (or both) in the mRNAs we have shown as regulated by only PUM1 or PUM2. In different sheets we have listed different mRNAs which reached the threshold, to possibly being regulated by a particular RBP with PUM (e.g. SFPQ/PUM1 or FXR1/PUM1 from 1st sheet).

Table S6 Gene ontology results related to Figure 4 Regulon model

This table presents Gene ontology (GO) results that have been used for building PUM1 and PUM2 RNA Regulon model shown on Figure 4. These GO results are based exclusively on experimentally confirmed interactions (ClueGo analysis in Cytoscape). Each sheet contains details of that GO analyses and shows the biological processes of mRNAs being possibly co-regulated by PUM1-RBP(A) or PUM2-RBP(B) etc.

Table S7 PUM1 and PUM2 RIP-Seq identified mRNA targets

The 1st sheet (RIP-Seq mRNA targets) of this table contains list of mRNAs identified as PUM1- and/or PUM2-bound. PUM bound mRNAs are considered those the level of which was at least 2 times enriched in RIP-PUM1 or RIP-PUM2 in comparison to the negative control (RIP-IgG), and 2 times enriched in comparison to its level in TCam-2 transcriptome. The sheets 2nd-5th contain details of our RIP-Seq analysis made using Cuffdiff software/pipeline with pre-set Excel macros to select only mRNAs above the threshold. This type of presentation gives opportunity to the user to perform its own analyses by setting different parameters which are indicated in heading of each column.

Table S8 GO of RIPSeq siRNASeq Reg mRNAs protein interactors

This table contains details of Gene Ontology (GO) analyses using BiNGO plug-in in Cytoscape software. The consecutive sheets show GO tables of mRNAs identified by RIP-Seq, siRNA-Seq (mRNAs the level of which was changed upon PUMs siRNA knockdowns), regulated by PUMs mRNAs as well as PUM protein interactors which are bound in RNA dependent or independent manner. GO analyses are shown for Biological Processes (BP) and Molecular Functions (MF) of studied mRNA pools. Pathways and values which were visualized in yellow are shown on heat maps on **Figure 3**, **Supp Figure S5**, **Supp Figure S6** and **Supp Figure S7** are highlighted in yellow.

Table S9 mRNAs activated or repressed upon PUM1 or PUM2 siRNA KD

In this table, we have listed mRNAs up-regulated (called repressed) or down-regulated (called activated) upon PUM1 or PUM2 siRNA knockdown in the 1st and 2nd sheet. In the 3rd and 4th sheets, we have shown detailed Cuffdiff DGE analyses upon PUM1 KD (3rd sheet) and PUM2 KD (4th sheet) with pre-set Excel macros. The reader will be able to play around with the data from these analyses by setting different parameters listed in the 1st line of each table.

Table S10 mRNAs regulated by PUM1, PUM2 or PUM1 and PUM2

This table contains list of mRNAs we consider regulated by PUMs, as they were selected by both approaches: 1/ RIP-Seq and 2/ siRNA PUM knockdown. The 1st sheet contains the list of repressed mRNAs, while the 2nd one list of mRNAs activated by PUM1 and PUM2.

Table S11 PUM regulated mRNAs OE in TCam-2 cells and infertility

This table contains list of mRNAs studied by RT-qPCR (for Figure 5 and 6). The 1st sheet (called PUM targets OE in TCam-2) contains list of 19 mRNAs regulated by PUM1 or PUM2, with indicated presence or absence of PBE, function and reference for that function. These (PUM targets OE in TCam-2) mRNAs were selected based on their expression level being at least 6x higher in TCam-2 cells compared to the human testicular somatic tissue. The human testicular somatic tissue transcriptome was published by Irie et al.,2015. The 2nd sheet contains 3 lists of mRNAs: 1/ PUM targets OE in TCam-2, 2/ PUM1 and 3/ PUM2-regulated. On the right there is Venn diagram showing overlap of those three pools. The 3rd sheet (called PUM targets and infertility) contains PUM1 or/and PUM2-regulated 9 mRNAs encoding genes which when mutated caused male infertility (according to Matzuk et al., 2008). Their PBE content, function and reference are provided. The 4th sheet contains same type of data as the 2nd. The venn diagram presents the overlap between 501 mouse and human infertility genes (columns C,D and E) and PUM1 and PUM2 regulated mRNAs in TCam-2 (Matzuk et al.,2008).

Table S12 RIP-Seq PUM targets versus ENCODE CLIP targets of PUM interacting proteins

This table describes comparison between our RIP-Seq identified PUM1- and PUM2-bound mRNAs from TCam-2 cells with CLIP-identified PUM co-factor mRNA targets from ENCODE data. The 1st sheet (ENCODE K562 eCLIP) presents % of overlapping mRNA targets between PUM1 or PUM2 and FMR1, FXR1, FXR2, HNRNPA1, MATR3, PABPC4, PTBP1, SRSF1, or SRSF7 interactors based on K562 cell line. The 2nd sheet (TCam-2 RIP-Seq vs ENCODE eCLIP) presents comparison between PUM1 and PUM2 targets from our TCam-2 RIP-Seq analyses with ENCODE eCLIP data for FMR1, FXR1, FXR2, HNRNPA1, MATR3, PABPC4, PTBP1 and SRSF1, and SRSF7 protein mRNA targets from HEPG2 and K562 cell lines.
