# Supplementary material for: Characterization of RNP Networks of PUM1 and PUM2 Post-Transcriptional Regulators in TCam-2 Cells, a Human Male Germ Cell Model
Source: Cells. 2020 Apr 16;9(4):984. doi: 10.3390/cells9040984 (PMC7226987; doi:10.3390/cells9040984)
Supplement: Supplementary file 1 [file cells-09-00984-s001.zip › FINAL Supplementary Files.zip!/FINAL Supplementary Files/References in Supplementary file Table S11.pdf]

References to supplementary Table S11

1. Ruiz-Lafuente, N.; Minguela, A.; Parrado, A. DOCK9 induces membrane ruffles and Rac1 activity in cancer HeLa epithelial cells. *Biochem Biophys Rep* **2018**, *14*, 178-181, doi:10.1016/j.bbrep.2018.05.004.
2. Wang, H.; Fu, Z.; Dai, C.; Cao, J.; Liu, X.; Xu, J.; Lv, M.; Gu, Y.; Zhang, J.; Hua, X., et al. LncRNAs expression profiling in normal ovary, benign ovarian cyst and malignant epithelial ovarian cancer. *Sci Rep* **2016**, *6*, 38983, doi:10.1038/srep38983.
3. Li, Y.H.; Liu, Y.; Li, Y.D.; Liu, Y.H.; Li, F.; Ju, Q.; Xie, P.L.; Li, G.C. GABA stimulates human hepatocellular carcinoma growth through overexpressed GABAA receptor theta subunit. *World J Gastroenterol* **2012**, *18*, 2704-2711, doi:10.3748/wjg.v18.i21.2704.
4. Lei, Y.; Henderson, B.R.; Emmanuel, C.; Harnett, P.R.; deFazio, A. Inhibition of ANKRD1 sensitizes human ovarian cancer cells to endoplasmic reticulum stress-induced apoptosis. *Oncogene* **2015**, *34*, 485-495, doi:10.1038/onc.2013.566.
5. Tsai, M.H.; Chen, W.C.; Yu, S.L.; Chen, C.C.; Jao, T.M.; Huang, C.Y.; Tzeng, S.T.; Yen, S.J.; Yang, Y.C. DNA Hypermethylation of SHISA3 in Colorectal Cancer: An Independent Predictor of Poor Prognosis. *Ann Surg Oncol* **2015**, *22 Suppl 3*, S1481-1489, doi:10.1245/s10434-015-4593-1.
6. Schultess, J.; Danielewski, O.; Smolenski, A.P. Rap1GAP2 is a new GTPase-activating protein of Rap1 expressed in human platelets. *Blood* **2005**, *105*, 3185-3192, doi:10.1182/blood-2004-09-3605.
7. Nadiminty, N.; Lou, W.; Sun, M.; Chen, J.; Yue, J.; Kung, H.J.; Evans, C.P.; Zhou, Q.; Gao, A.C. Aberrant activation of the androgen receptor by NF-kappaB2/p52 in prostate cancer cells. *Cancer Res* **2010**, *70*, 3309-3319, doi:10.1158/0008-5472.CAN-09-3703.
8. Pisanic, T.R., 2nd; Cope, L.M.; Lin, S.F.; Yen, T.T.; Athamanolap, P.; Asaka, R.; Nakayama, K.; Fader, A.N.; Wang, T.H.; Shih, I.M., et al. Methylomic Analysis of Ovarian Cancers Identifies Tumor-Specific Alterations Readily Detectable in Early Precursor Lesions. *Clin Cancer Res* **2018**, *24*, 6536-6547, doi:10.1158/1078-0432.CCR-18-1199.
9. Goriely, A.; Hansen, R.M.; Taylor, I.B.; Olesen, I.A.; Jacobsen, G.K.; McGowan, S.J.; Pfeifer, S.P.; McVean, G.A.; Rajpert-De Meyts, E.; Wilkie, A.O. Activating mutations in FGFR3 and HRAS reveal a shared genetic origin for congenital disorders and testicular tumors. *Nat Genet* **2009**, *41*, 1247-1252, doi:10.1038/ng.470.
10. Kim, S.; Yang, J.W.; Kim, C.; Kim, M.G. Impact of suppression of tumorigenicity 14 (ST14)/serine protease 14 (Prss14) expression analysis on the prognosis and management of estrogen receptor negative breast cancer. *Oncotarget* **2016**, *7*, 34643-34663, doi:10.18632/oncotarget.9155.
11. Liu, X.; Li, C.; Zhang, R.; Xiao, W.; Niu, X.; Ye, X.; Li, Z.; Guo, Y.; Tan, J.; Li, Y. The EZH2-H3K27me3-DNMT1 complex orchestrates epigenetic silencing of the wwc1 gene, a Hippo/YAP pathway upstream effector, in breast cancer epithelial cells. *Cell Signal* **2018**, *51*, 243-256, doi:10.1016/j.cellsig.2018.08.011.
12. Visser-Grieve, S.; Hao, Y.; Yang, X. Human homolog of Drosophila expanded, hEx, functions as a putative tumor suppressor in human cancer cell lines independently of the Hippo pathway. *Oncogene* **2012**, *31*, 1189-1195, doi:10.1038/onc.2011.318.
13. Liang, Z.; Zhan, Y.; Shen, Y.; Wong, C.C.; Yates, J.R., 3rd; Plattner, F.; Lai, K.O.; Ip, N.Y. The pseudokinase CaMKv is required for the activity-dependent maintenance of dendritic spines. *Nat Commun* **2016**, *7*, 13282, doi:10.1038/ncomms13282.
14. Boylan, K.L.; Buchanan, P.C.; Manion, R.D.; Shukla, D.M.; Braumberger, K.; Bruggemeyer, C.; Skubitz, A.P. The expression of Nectin-4 on the surface of ovarian cancer cells alters their ability to adhere, migrate, aggregate, and proliferate. *Oncotarget* **2017**, *8*, 9717-9738, doi:10.18632/oncotarget.14206.

15. Kiang, K.M.; Leung, G.K. A Review on Adducin from Functional to Pathological Mechanisms: Future Direction in Cancer. *Biomed Res Int* **2018**, *2018*, 3465929, doi:10.1155/2018/3465929.
16. Yi, Y.; Nandana, S.; Case, T.; Nelson, C.; Radmilovic, T.; Matusik, R.J.; Tsuchiya, K.D. Candidate metastasis suppressor genes uncovered by array comparative genomic hybridization in a mouse allograft model of prostate cancer. *Mol Cytogenet* **2009**, *2*, 18, doi:10.1186/1755-8166-2-18.
17. Aker, M.; Rouvinski, A.; Hashavia, S.; Ta-Shma, A.; Shaag, A.; Zenvirt, S.; Israel, S.; Weintraub, M.; Taraboulos, A.; Bar-Shavit, Z., et al. An SNX10 mutation causes malignant osteopetrosis of infancy. *J Med Genet* **2012**, *49*, 221-226, doi:10.1136/jmedgenet-2011-100520.
18. Martemyanov, K.A.; Arshavsky, V.Y. Biology and functions of the RGS9 isoforms. *Prog Mol Biol Transl Sci* **2009**, *86*, 205-227, doi:10.1016/S1877-1173(09)86007-9.
19. Obeidat, M.; Li, L.; Ballermann, B.J. TIMAP promotes angiogenesis by suppressing PTEN-mediated Akt inhibition in human glomerular endothelial cells. *Am J Physiol Renal Physiol* **2014**, *307*, F623-633, doi:10.1152/ajprenal.00070.2014.
20. Wong, E.Y.; Tse, J.Y.; Yao, K.M.; Tam, P.C.; Yeung, W.S. VCY2 protein interacts with the HECT domain of ubiquitin-protein ligase E3A. *Biochem Biophys Res Commun* **2002**, *296*, 1104-1111, doi:10.1016/s0006-291x(02)02040-5.
21. Kawa, S.; Ito, C.; Toyama, Y.; Maekawa, M.; Tezuka, T.; Nakamura, T.; Nakazawa, T.; Yokoyama, K.; Yoshida, N.; Toshimori, K., et al. Azoospermia in mice with targeted disruption of the Brek/Lmtk2 (brain-enriched kinase/lemur tyrosine kinase 2) gene. *Proc Natl Acad Sci U S A* **2006**, *103*, 19344-19349, doi:10.1073/pnas.0603603103.
22. Beleza-Meireles, A.; Lundberg, F.; Lagerstedt, K.; Zhou, X.; Omrani, D.; Frisen, L.; Nordenskjold, A. FGFR2, FGF8, FGF10 and BMP7 as candidate genes for hypospadias. *Eur J Hum Genet* **2007**, *15*, 405-410, doi:10.1038/sj.ejhg.5201777.
23. Kawagoe, J.; Li, Q.; Mussi, P.; Liao, L.; Lydon, J.P.; DeMayo, F.J.; Xu, J. Nuclear receptor coactivator-6 attenuates uterine estrogen sensitivity to permit embryo implantation. *Dev Cell* **2012**, *23*, 858-865, doi:10.1016/j.devcel.2012.09.002.
24. Bonache, S.; Martinez, J.; Fernandez, M.; Bassas, L.; Larriba, S. Single nucleotide polymorphisms in succinate dehydrogenase subunits and citrate synthase genes: association results for impaired spermatogenesis. *Int J Androl* **2007**, *30*, 144-152, doi:10.1111/j.1365-2605.2006.00730.x.
25. Sun, T.; Xin, Z.; Jin, Z.; Wu, Y.; Gong, Y. Effect of TGF-beta/Smad signaling on sertoli cell and possible mechanism related to complete sertoli cell-only syndrome. *Mol Cell Biochem* **2008**, *319*, 1-7, doi:10.1007/s11010-008-9869-3.
26. Held, T.; Paprotta, I.; Khulan, J.; Hemmerlein, B.; Binder, L.; Wolf, S.; Schubert, S.; Meinhardt, A.; Engel, W.; Adham, I.M. Hspa4l-deficient mice display increased incidence of male infertility and hydronephrosis development. *Mol Cell Biol* **2006**, *26*, 8099-8108, doi:10.1128/MCB.01332-06.
27. Black, L.D.; Nudell, D.M.; Cha, I.; Cherry, A.M.; Turek, P.J. Compound genetic factors as a cause of male infertility: case report. *Hum Reprod* **2000**, *15*, 449-451, doi:10.1093/humrep/15.2.449.
