# Supplementary material for: Characterization of RNP Networks of PUM1 and PUM2 Post-Transcriptional Regulators in TCam-2 Cells, a Human Male Germ Cell Model
Source: Cells. 2020 Apr 16;9(4):984. doi: 10.3390/cells9040984 (PMC7226987; doi:10.3390/cells9040984)
Supplement: Supplementary file 1 [file cells-09-00984-s001.zip › FINAL Supplementary Files.zip!/FINAL Supplementary Files/Supplementary Certificate of Mass Spectrometry Analysis.pdf]

Certificate of analysis for protein identification by mass spectrometry

Database Search Parameters

1: Precursor Tolerance Unit: ppm

2: Precursor Ion m/z Tolerance: 30.0

3: Fragment Ion Tolerance Unit: Da

4: Fragment Ion m/z Tolerance: 0.1

5: Cleavage: Enzyme

6: Enzyme: Trypsin

7: Missed Cleavages: 1

8: Specificity: Specific

9: Database: uniprot-human-reviewed-june-2017-crap.fasta_concatenated_target_decoy.fasta

10: Forward Ion: b

11: Rewind Ion: y

12: Fixed Modifications: Carbamidomethylation of C

13: Variable Modifications: Oxidation of M

14: Refinement Variable Modifications: Acetylation of protein N-term, Pyrolidone from E, Pyrolidone from Q, Pyrolidone from carbamidomethylated C

15: Refinement Fixed Modifications: Carbamidomethylation of C

Input Filters

1: Minimal Peptide Length: 8

2: Maximal Peptide Length: 30

3: Precursor m/z Tolerance: 30.0

4: Precursor m/z Tolerance Unit: Yes

5: Unrecognized Modifications Discarded: Yes

Spectrum Counting Parameters

1: Method: NSAF

2: Validated Matches Only: No

Annotation Settings

1: Intensity Limit: 0.75

2: Automatic Annotation: Yes

3: Selected Ions: y, b

4: Neutral Losses: H_2O, NH_3, CH_4OS

5: Neutral Losses Sequence Dependence: Yes

6: Fragment Ion m/z Tolerance: 0.1
