# Supplementary material for: Characterization of RNP Networks of PUM1 and PUM2 Post-Transcriptional Regulators in TCam-2 Cells, a Human Male Germ Cell Model
Source: Cells. 2020 Apr 16;9(4):984. doi: 10.3390/cells9040984 (PMC7226987; doi:10.3390/cells9040984)
Supplement: Supplementary file 1 [file cells-09-00984-s001.zip › FINAL Supplementary Files.zip!/FINAL Supplementary Files/Supplementary full western blot for Figure 1E.pdf]

Whole blots for Figure 1E

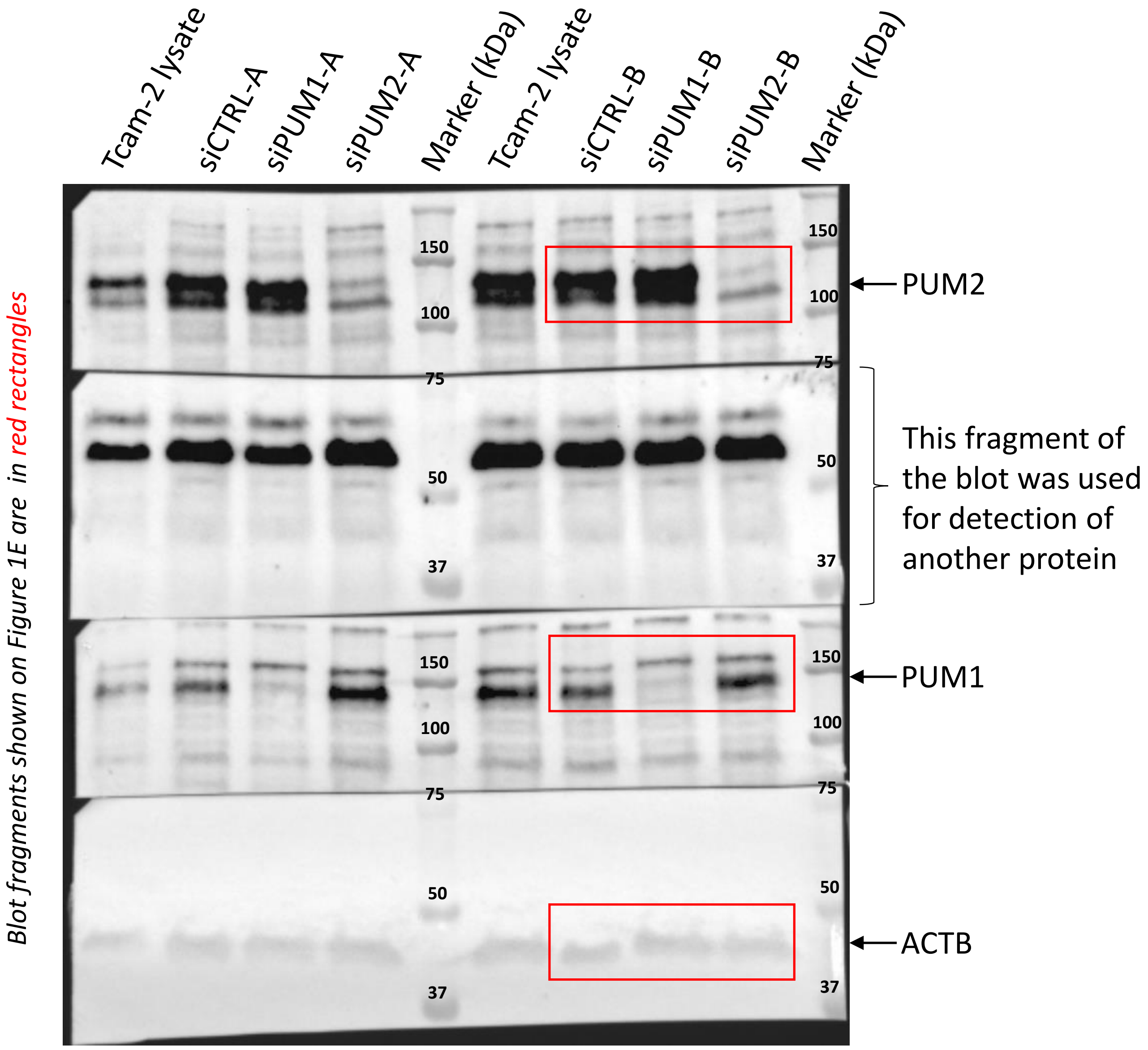

80sec exposition
