# Supplementary material for: Characterization of RNP Networks of PUM1 and PUM2 Post-Transcriptional Regulators in TCam-2 Cells, a Human Male Germ Cell Model
Source: Cells. 2020 Apr 16;9(4):984. doi: 10.3390/cells9040984 (PMC7226987; doi:10.3390/cells9040984)
Supplement: Supplementary file 1 [file cells-09-00984-s001.zip › FINAL Supplementary Files.zip!/FINAL Supplementary Files/Supplementary_Figures FINAL REV.pdf]

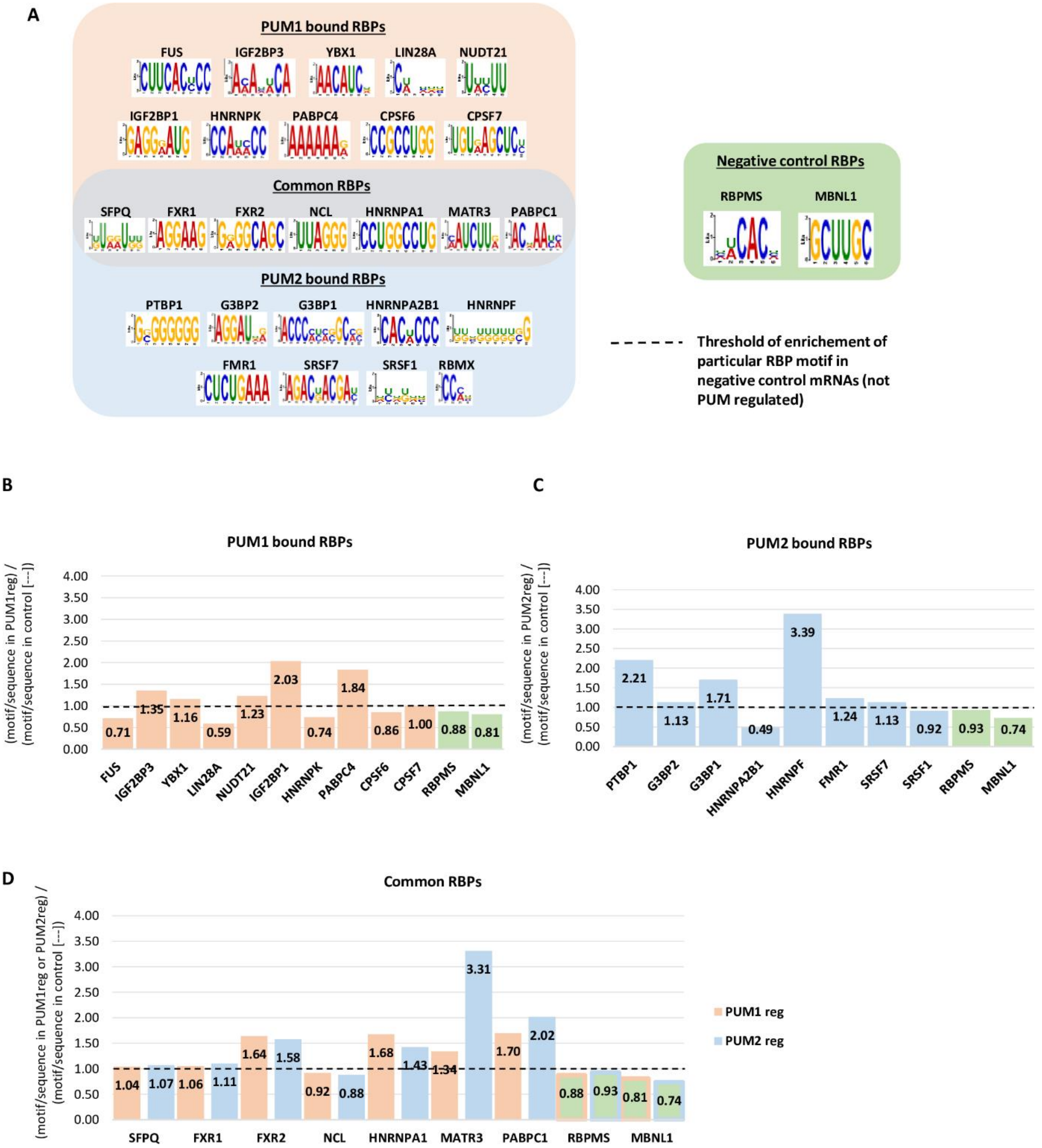

Figure S1: Enrichment of binding motifs for PUM1- or PUM2-interacting RBPs identified in each mRNA regulated by PUM1 or PUM2 in TCam-2 cells. The entire sequence of each mRNA was searched for motifs shown in **(A).** Each enrichment analysis was performed using FIMO software with a threshold set at P-value<0.01. Motif enrichment values of particular RBPs in mRNA from the TCam-2 transcriptome not regulated by PUMs were set at 1, and this was the baseline control (shown with dashed line). **(A)** Representative motifs corresponding to RBPs (used in this analysis) and interacting with PUM1 and/or PUM2 were generated using MEME software. **(B)** Histograms representing the enrichment of binding motifs of PUM1-interacting RBPs present in mRNAs regulated by PUM1 are in pink, and in negative controls (RBPMS and MBNL1), not interacting with PUMs, present in mRNAs regulated by PUM1 are in green. **(C)** Histograms representing enrichment of binding motifs of PUM2-interacting RBPs present in mRNAs regulated by PUM2 are in blue, and in negative controls (RBPMS and MBNL1), not interacting with PUMs, in mRNAs regulated by PUM2 are in green. **(D)** Enrichment of binding motifs of RBPs interacting with both, PUM1 and PUM2, in mRNAs regulated by both, PUM1 (pink) and PUM2 (blue), and in negative controls in mRNAs regulated by both, PUM1 (green with pink contour) and PUM2 (green with blue contour).

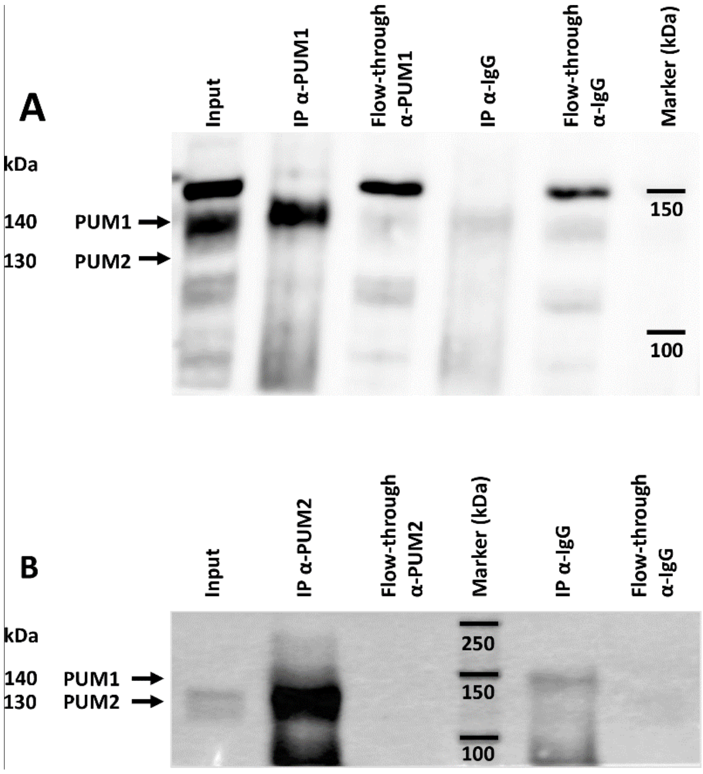

Figure S2: Western blot (WB) analyses showing specificity of PUM1 and PUM2 antibodies, as well as PUM binding efficiency to beads. (A) WB detection of PUM1 on beads after co-immunoprecipitation (co-IP) using anti-PUM1 antibody. **(B)** WB detection of PUM2 on beads after Co-IP using anti-PUM2 antibody.

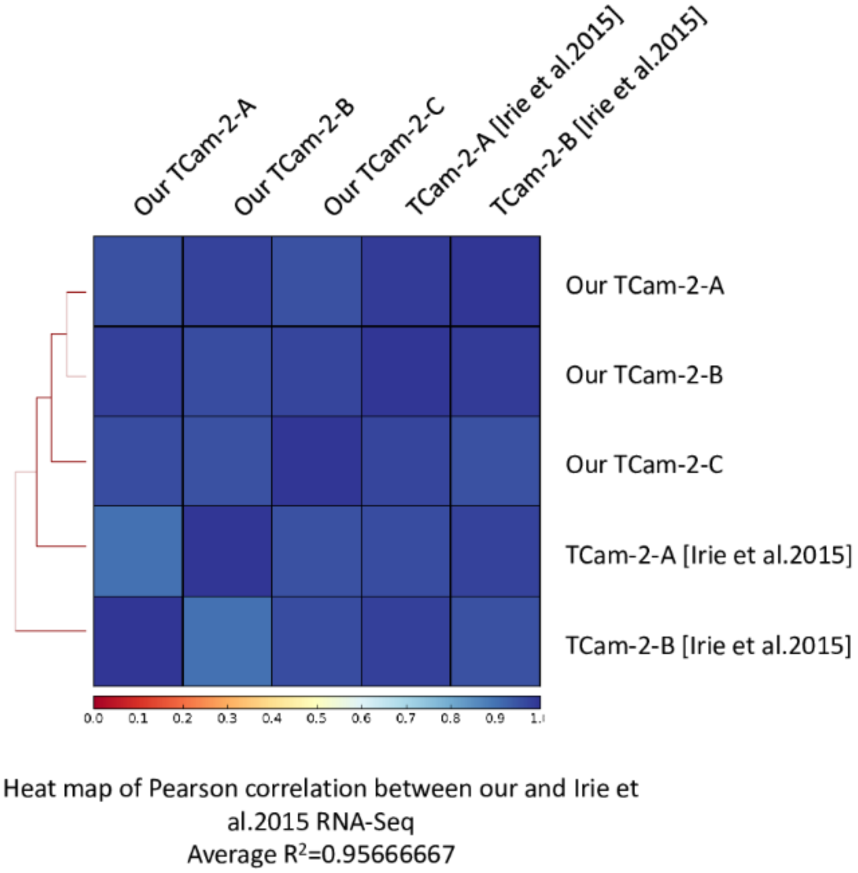

Figure S3: Heat map of Pearson correlation for comparison of the TCam-2 transcriptome obtained in this study with the TCam-2 transcriptome published by Irie et al.,2015 [30].

A

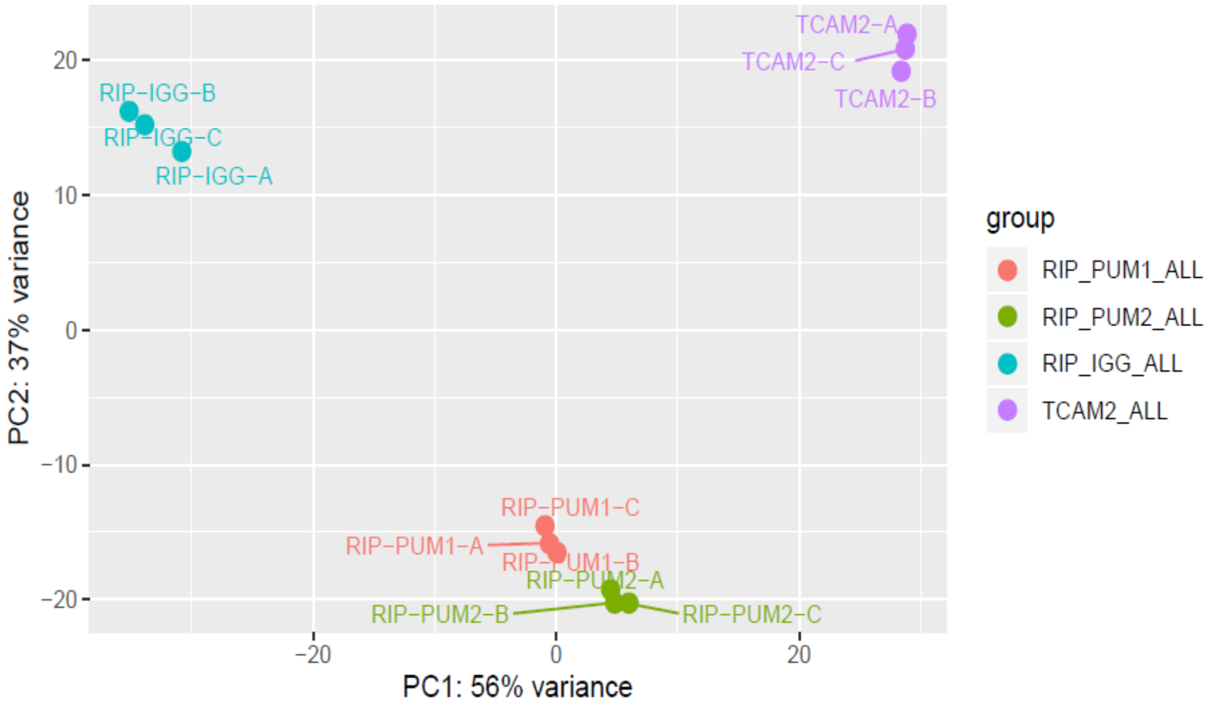

B

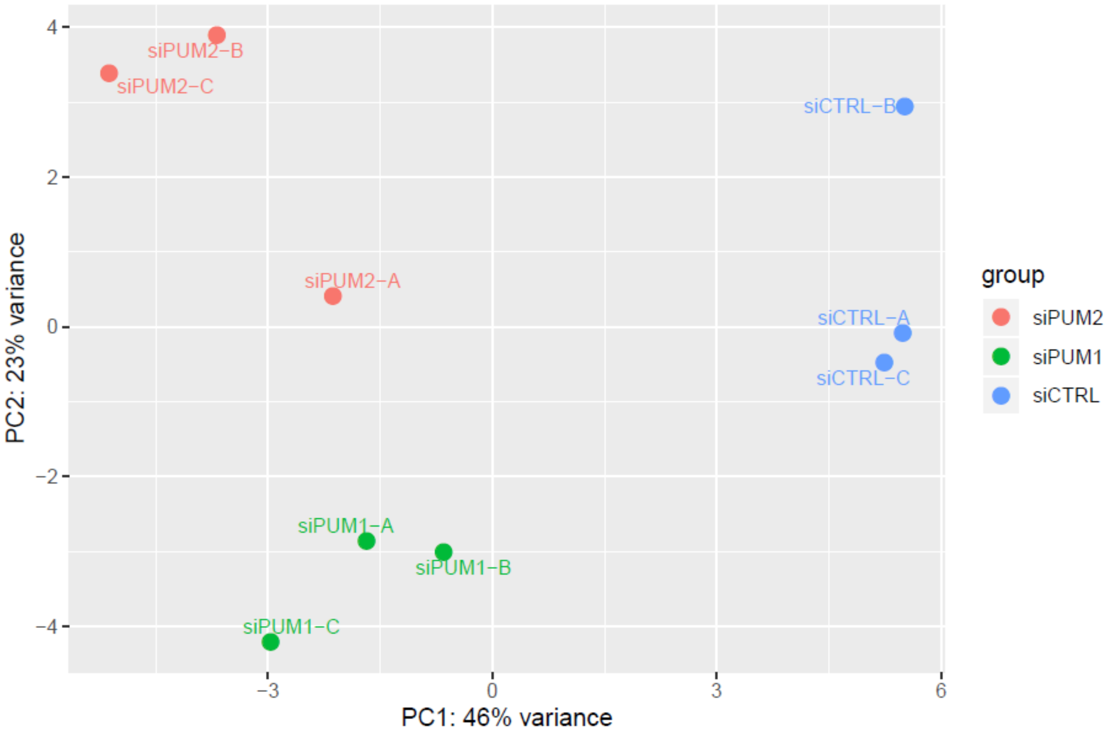

Figure S4: PCA plots showing similarity between the studied biological replicates of RIP anti –PUM1, -PUM2, -IgG and Tcam-2 transcriptome **(A)** as well as in the biological replicates of siRNA knockdowns of PUM1 (siPUM1) and PUM2 (siPUM2) in comparison to control (siCTRL) **(B).**

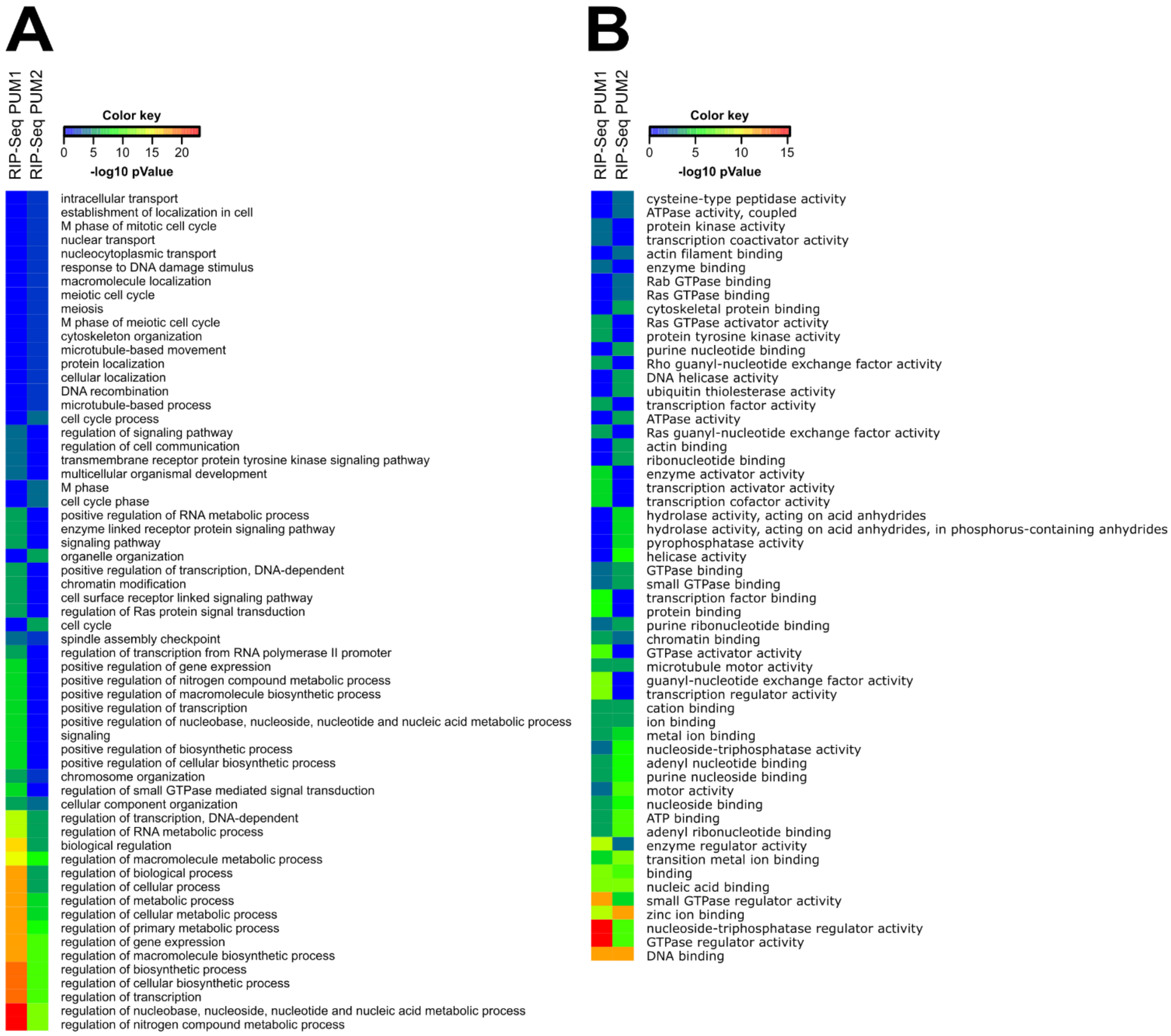

Figure S5: Gene Ontology analysis of mRNAs bound to PUM1 and PUM2 as identified by the RIP-Seq approach. (A) Heatmap of BiNGO analysis of TOP40 Biological Processes of mRNAs selected as bound to PUM1 and PUM2. **(B)** Heatmap of BiNGO analysis of TOP40 Molecular Functions of mRNAs selected as bound to PUM1 and PUM2. The detailed results of the GO analysis are presented in **Supp TableS8**.

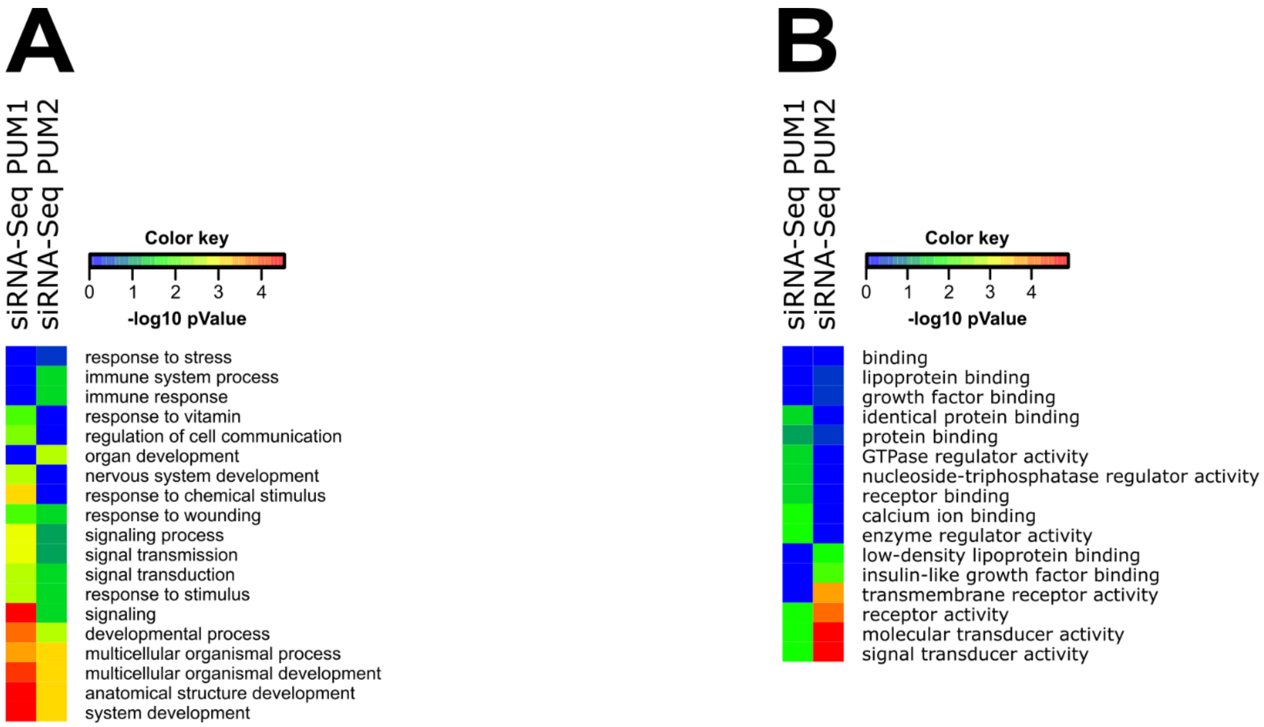

Figure S6: Gene Ontology analysis of mRNAs up-and downregulated upon PUM1 and/or PUM2 siRNA knockdown (PUM1 or PUM2 siRNA- Seq). (A) Heatmap of BiNGO analysis of TOP15 Biological Processes of mRNAs selected as up- and downregulated upon PUM1 or PUM2 siRNA knockdown. **(B)** Heatmap of BiNGO analysis of TOP10 Molecular Functions of mRNAs selected as up- and downregulated upon PUM1 or PUM2 siRNA. The detailed results of the GO analysis are presented in **Supp Table S8**.

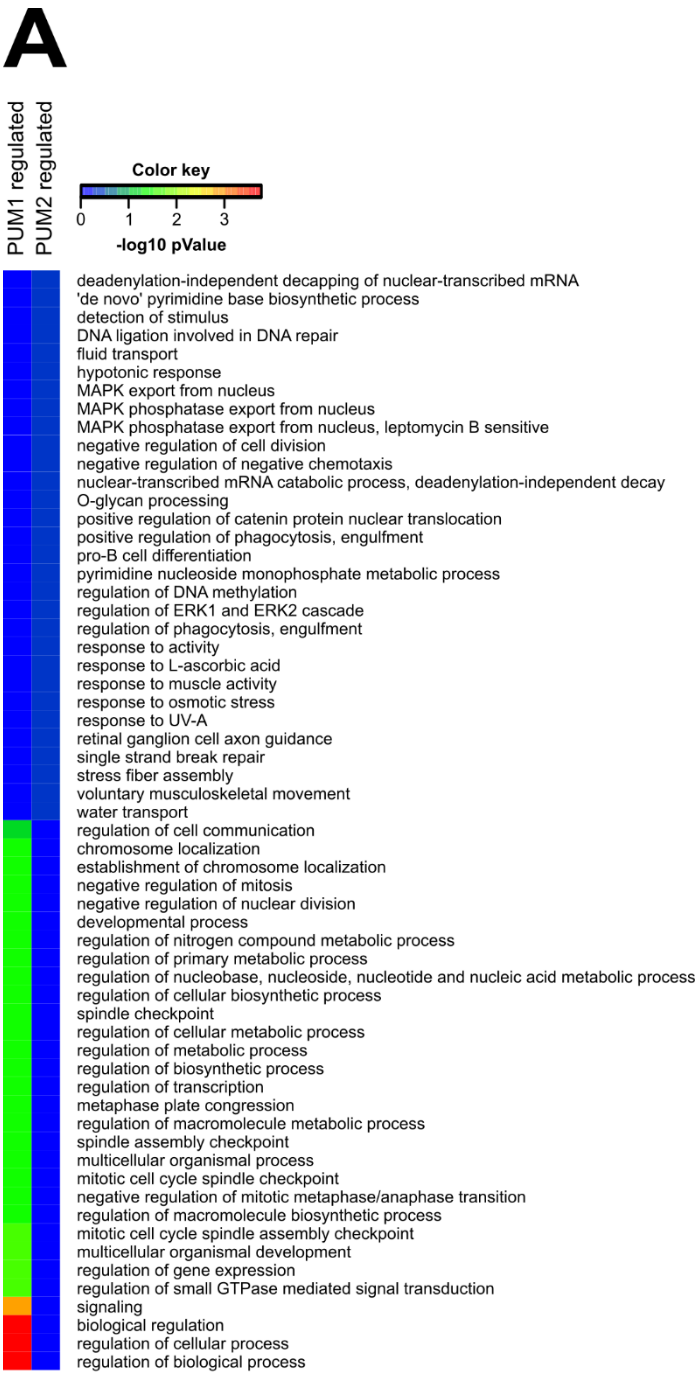

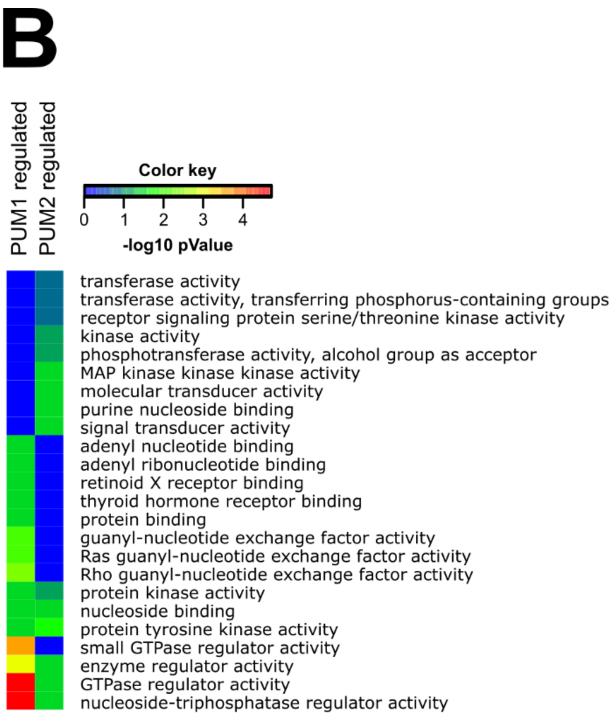

Figure S7: Gene Ontology analysis of PUM1-and PUM2- regulated targets. (A) Heatmap of BiNGO analysis of TOP30 Biological Processes of mRNAs regulated by PUM1 and PUM2. **(B)** Heatmap of BiNGO analysis of TOP15 Molecular Functions of mRNAs regulated by PUM1 and PUM2. The detailed results of the GO analysis are presented in **Supp Table S8**.
